# A post-translational regulatory map of chronic antigen-driven human T cell dysfunction

**DOI:** 10.64898/2026.03.04.709614

**Published:** 2026-03-06

**Authors:** Hiroyuki Kojima, Charlotte R. Wayne, Luis F. Somarribas Patterson, Henry Sanford, Tzu-Jou Chen, Ya-Hui Lin, Joshua D. Schoenfeld, Lisa H. F. McGary, Yan-Ting Chen, Korbinian N. Kropp, Beatrice Zhang, Jahan Rahman, Tiffany L. Zhang, Nathalie Ropek, Cameron Roberts, Yuxi Ai, Kartikeya M. Menon, A. Ari Hakimi, Jiankun Lyu, Christopher A. Klebanoff, Omar Abdel-Wahab, Santosha A. Vardhana, Ekaterina V. Vinogradova

**Affiliations:** 1 These authors contributed equally; 2 Department of Chemical Immunology and Proteomics, The Rockefeller University, New York, New York, 10065, USA; 3 Immuno-Oncology Program, Memorial Sloan Kettering Cancer Center, New York, New York, 10065, USA; 4 These authors contributed equally; 5 Department of Medicine, Memorial Sloan Kettering Cancer Center, New York, New York, 10065, USA; 6 Gerstner Sloan Kettering Graduate School of Biomedical Sciences, New York, New York, 10065, USA; 7 Tri-Institutional MD-PhD Program, Memorial Sloan Kettering Cancer Center, New York, New York, 10065, USA; 8 Molecular Pharmacology Program, Sloan Kettering Institute, Memorial Sloan Kettering Cancer Center, New York, New York, 10065, USA; 9 Department of Biostatistics and Epidemiology, Memorial Sloan Kettering Cancer Center, New York, New York, 10065, USA; 10 Tri-Institutional PhD Program in Chemical Biology, New York, New York, 10021, USA; 11 The Evnin Family Laboratory of Computational Molecular Discovery, The Rockefeller University, New York, NY 10065, USA; 12 Immunogenomics and Precision Oncology Platform, Memorial Sloan Kettering Cancer Center, New York, NY 10065, USA; 13 Urology Service, Department of Surgery, Memorial Sloan Kettering Cancer Center, New York, NY 10065, USA; 14 Center for Cellular Immunotherapy, MSKCC, New York, New York, 10065, USA; 15 Parker Institute for Cancer Immunotherapy, New York, New York, 10065, USA; 16 Lead contact

**Keywords:** T cell exhaustion, chemical proteomics, metabolomics, mitochondria, redox, cysteine reactivity

## Abstract

T cells exposed to persistent antigen in the context of chronic viral infections or cancer lose self-renewal and cytotoxic capacity. Several transcriptional, epigenetic, and metabolic drivers of this process have been identified. However, the post-transcriptional regulatory mechanisms influencing the proteome of dysfunctional T cells are not well understood. Here we present a time-resolved molecular landscape of human T cells during the development of chronic antigen-driven dysfunction. Persistent T cell receptor stimulation significantly remodeled the proteome, including changes in canonical T cell exhaustion-associated proteins and proteins related to mitochondrial function, redox homeostasis, nucleotide metabolism, and cell-cycle progression. Dysfunctional T cells displayed activation of stress response pathways that were recapitulated *in vivo*; targeting these pathways altered the cytotoxic capacity of T cells during persistent tumor exposure. Our comprehensive proteomic resource reveals unique post-transcriptional changes in dysfunctional T cells and lays the groundwork for novel cysteine-directed therapeutics to enhance cancer immunotherapy.

## INTRODUCTION

Persistent T cell receptor (TCR) stimulation due to chronic antigen exposure is both necessary and sufficient to establish T cell dysfunction^1–4^. This concept was first suggested by the presence of tumor-infiltrating lymphocytes (TILs) that were unable to control tumor growth described by Hellstrom in 1968^5^. This finding challenged the idea that tumors could not be sensed by the host immune system, raising a fundamental biological question of why TILs fail to eliminate developing cancers. In the sixty years since this observation was first made, it has become increasingly appreciated that both CD4^+^ and CD8^+^ T cells responding to tumor cell-derived antigens are rendered dysfunctional upon tumor infiltration^6–9^, largely due to chronic TCR stimulation. Chronic TCR stimulation promotes the activity of transcription factors that drive expression of genes associated with T cell dysfunction, including TOX, NFAT, and TCF-1^10,11^, as well as sequential and progressive remodeling of the chromatin landscape^12–14^. The functionality of T cells can be recovered by withdrawing antigen early during chronic TCR stimulation, but T cells ultimately progress to an irreversible “scarred” state in which antigen withdrawal is insufficient to recover function^12,15^. Identifying early consequences of chronic TCR stimulation is therefore critical to preventing irreversible T cell dysfunction.

The dysfunctional T cell state has been linked to alterations in T cell metabolism. Either reduced availability of nutrients required for cell growth and proliferation or accumulation of metabolites that are toxic to lymphocytes can impact the persistence and function of intratumoral T cells^16–19^. Chronic antigenic stimulation is sufficient to compromise mitochondrial oxidative phosphorylation and drive a terminally dysfunctional state in mouse T cells^4,20,21^. Loss of mitochondrial ATP production during chronic TCR stimulation is likely to disrupt ATP-dependent cellular processes including protein synthesis and folding, which are among the most bioenergetically demanding processes in eukaryotic cells^22^. Moreover, loss of mitochondrial membrane potential, which has been described in dysfunctional T cells^21,23^, can lead to accumulation of reactive oxygen species (ROS), which can alter protein function by oxidizing proteinaceous thiols. However, the relationship between loss of mitochondrial capacity and dysfunction has not been extensively examined in human T cells.

In this study, we build a multidimensional atlas of time-resolved molecular signatures in human T cells undergoing chronic TCR stimulation and progressive T cell dysfunction. This atlas integrates transcriptional, metabolomic, proteomic, and chemical proteomic characterization of activated, acutely, and chronically stimulated T cell states. Among these approaches, we employed advanced cysteine-targeting chemical proteomic workflows – activity-based protein profiling with sample multiplexing using tandem mass tags (TMT-ABPP)^24,25^ – to investigate the evolving functional proteomic landscapes of dysfunctional human T cells. We recently deployed this approach to characterize reactivity changes following acute TCR stimulation, demonstrating that cysteine can serve as a “sensor” for structural and functional protein changes under different conditions, including shifts in cellular redox homeostasis^25^. In the current study, we demonstrate extensive remodeling of both the T cell proteome and cysteine reactivity landscape during chronic TCR stimulation. This integrated analysis combined with extensive functional follow up studies enabled identification of novel mechanistic links between metabolic phenotypes and functional alterations in dysfunctional T cells. Using this unique dataset, we uncovered previously undescribed regulatory mechanisms by which human T cells adapt to chronic antigen stimulation, which have been further validated in primary human tumor-infiltrating lymphocytes from kidney cancer patients. Together, these findings demonstrate the value of functional proteomic characterization in identifying novel therapeutic targets to reverse chronic antigen-driven human T cell dysfunction.

## RESULTS

### Persistent TCR stimulation drives human T cell dysfunction *in vitro*.

**Figure 1.**
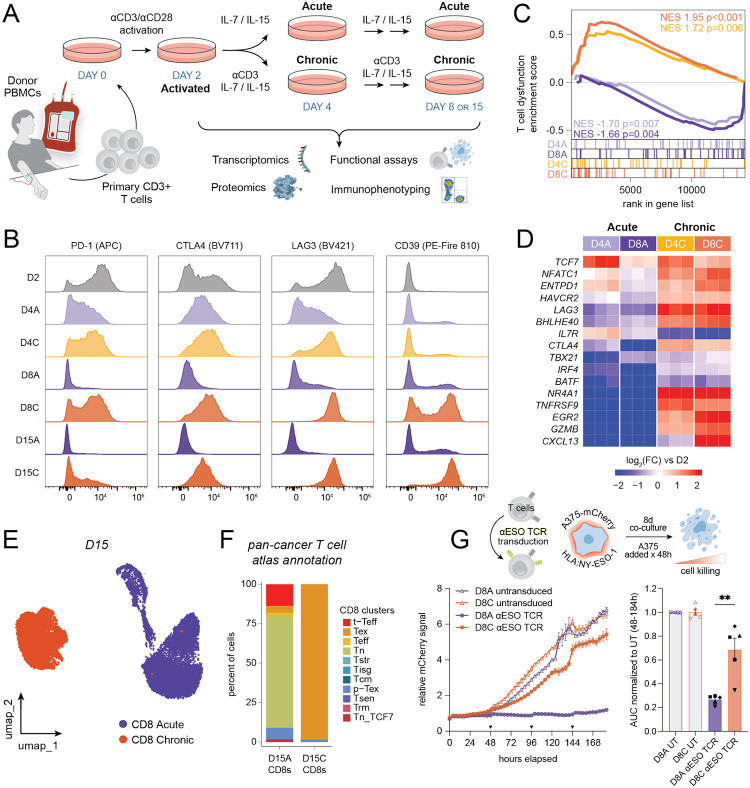
Robust *in vitro* platform for generating dysfunctional human T cells following chronic TCR stimulation. (A) Experimental setup for generation and multi-omic profiling of primary human T cells undergoing cytokine-mediated expansion with or without chronic TCR stimulation. (B) Expression of activation and exhaustion markers by flow cytometry on CD8^+^ T cells from days 2, 4, 8, and 15 of “acute” (A) and “chronic” (C) TCR stimulation conditions. Histograms are normalized to mode. (C) Gene Set Enrichment Analysis (GSEA) on a list of 43 genes in the “chronic activation program” associated with pan-cancer T cell dysfunction^1^. Each condition is compared to D2. NES – normalized enrichment score. (D) Heatmap displaying a selection of chronic activation program genes differentially changing in D4 and D8 T cells cultured with or without chronic stimulation relative to activated T cells (D2). (E) Uniform Manifold Approximation and Projection (UMAP) of single-cell transcriptomes from D15A and D15C CD8^+^ T cells. (F) Annotation of acute and chronic CD8^+^ T cell phenotypes (D15A and D15C) based on label transfer of single-cell transciptomic data from Chu et al.^2^ (G) Antigen-specific cancer cell killing by D8A and D8C T cells transduced with NY-ESO-1-specific TCR. On the left, a representative cell viability curve of NY-ESO-1-expressing A375 melanoma cells engineered to express mCherry following 184 hours of co-culture with T cells, normalized to 0 h condition. Inverted triangles on x axis indicate timepoints when A375 melanoma cells where added to the co-culture. On the right, area under the curve (AUC) of relative mCherry signal over 48–184 hours for n = 5 independent donors, normalized to untransduced (UT) D8A. Statistical comparison by two-tailed paired t test (** p < 0.01). Donor-matched conditions are indicated by differently shaped data points.

We first sought out to develop an *in vitro* system that would allow us to systematically generate dysfunctional human T cells following chronic TCR stimulation together with both activated and acutely stimulated T cells, which were used as key comparison groups, as they represent complementary states with differences in both effector function and metabolic activity. CD3^+^ T cells were isolated by negative selection from peripheral blood mononuclear cells (PBMCs) from human donors, activated with αCD3 and αCD28 antibodies for 2 days (“activated” state, D2), and then split into two pools that were expanded for 2, 6, and 13 days either in the presence of IL-7 and IL-15 cytokines (“acute” state, D4A, D8A, and D15A) or in the presence of IL-7 and IL-15 along with continued TCR stimulation with αCD3 antibody (“chronic” state, D4C, D8C, and D15C) (Figure 1A). We then performed comprehensive characterization of these T cell states using transcriptional, metabolomic, and proteomic platforms. Our previous work using mouse T cells suggested that persistent stimulation over the course of 8 days was sufficient to generate dysfunctional T cells^4^. To confirm that chronic TCR stimulation induced a dysfunctional human T cell program, we performed high-dimensional spectral flow cytometry on T cells cultured for varying amounts of time since the initial stimulation. Indeed, culturing of activated human T cells in media containing IL-7 and IL-15 together with persistent TCR stimulation (“Chronic”) was sufficient to suppress T cell proliferation (Figures S1A, S1B) and increase expression of multiple exhaustion markers, including inhibitory receptors (PD-1, LAG3, CTLA4, CD39) and transcription factors (TOX, T-Bet/TBX21) associated with human T cell dysfunction (Figures 1B, S1C–S1E). By contrast, T cells cultured with IL-7 and IL-15 without additional TCR stimulation (“Acute”) exhibited a progressive decline in expression of inhibitory receptors associated with T cell dysfunction (PD-1, CTLA4, LAG3), but not activation (HLA-DR, CD38) (Figures 1B, S1C–S1E). Chronic TCR stimulation was also sufficient to reduce intracellular accumulation of inflammatory cytokines including TNF, IFNγ, and IL-2 following restimulation with phorbol myristate acetate and ionomycin (PMA-I) (Figures S1C–S1E).

To determine whether chronic antigenic stimulation was sufficient to more broadly alter human T cell differentiation, we performed bulk RNA-sequencing of primary human T cells cultured for 2–8 days as described in Figure 1A (Data S1). Principal component analysis (PCA) demonstrated that the transcriptome of acute and chronic T cells diverged within 48 hours of chronic TCR stimulation (Figure S1F). Chronic stimulation led to progressive upregulation of genes uniquely associated with T cell dysfunction (“chronic activation score”) from a recently published single-cell atlas of tumor-infiltrating CD8^+^ T cells across seven cancer types^2^, whereas culturing cells in IL-7 and IL-15 alone led to a progressive loss of enrichment in this gene set (Figure 1C). Chronic stimulation increased expression of transcription factors associated with T cell dysfunction, including *NFATC1*, *BHLHE40*, *NR4A1*, and *EGR2*^26–28^, as well as paracrine factors such as *GZMB* and *CXCL13* (Figure 1D). We corroborated these findings by performing single-cell RNA sequencing of cells expanded in the absence or presence of chronic stimulation for 15 days and comparing single-cell transcriptomes with T cells isolated from 324 patients across 16 tumor types (Figure 1E)^7^. While acute T cell transcriptomes mapped to a variety of cell states, nearly all chronically stimulated CD8^+^ T cells most closely mapped to exhausted CD8^+^ T cells from patient tumors (Figures 1F, S1G).

To directly measure the impact of chronic TCR stimulation on T cell effector capacity, we adapted an established, antigen-specific serial cytotoxicity assay. In this system, human T cells transduced with the affinity-enhanced, NY-ESO-1 cancer testis antigen-specific, HLA-A*02:01-restricted transgenic TCR (1G4-LY^29^) were acutely or chronically stimulated as described in Figure 1A and then co-cultured with NY-ESO-1^+^/HLA-A*02:01^+^ expressing cancer cell lines A375 and SK37 for an additional eight days, with replenishment of cancer cells every 2 days (Figures 1G, S1H). Re-stimulated acute T cells (D8A) killed target cells in a TCR-specific fashion, while chronically stimulated T cells (D8C) were markedly impaired in killing capacity (Figures 1G, S1H). Collectively, these results demonstrate that chronic TCR stimulation *in vitro* recapitulates multiple hallmarks of *in vivo* T cell exhaustion, including reduced proliferative capacity, increased inhibitory receptor expression, decreased production of effector cytokines, and reduced cytotoxicity.

### Proteomic analysis identifies transcriptome-independent changes in protein expression during chronic TCR stimulation.

**Figure 2.**
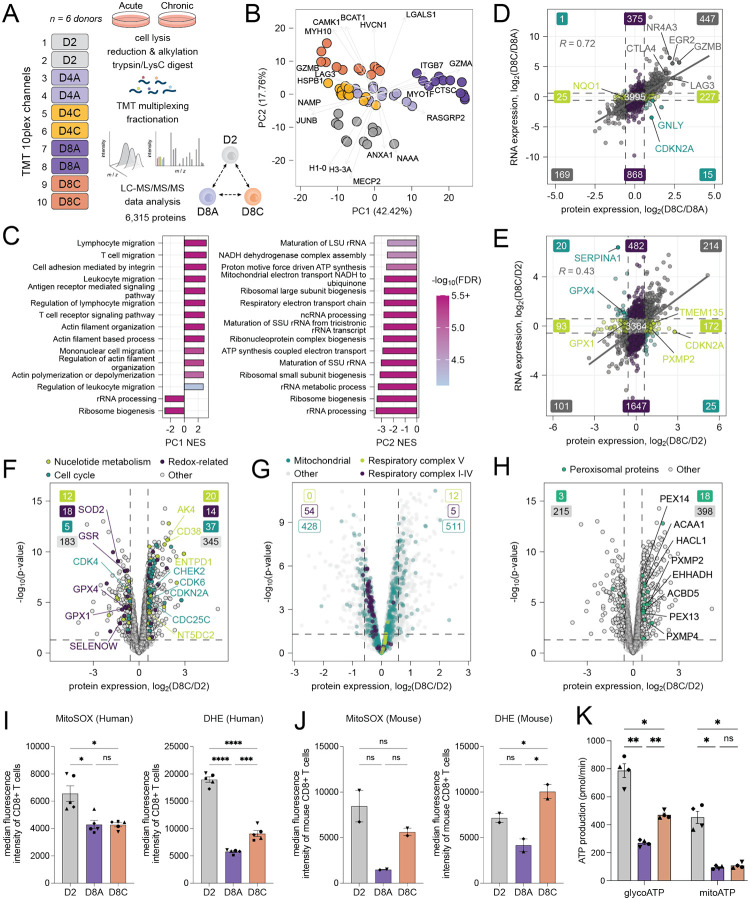
Transcriptional and proteomic changes at early and late stages of *in vitro* T cell dysfunction. (A) Experimental schematic of TMT-multiplexed unenriched proteomics of primary human T cells cultured with and without chronic TCR stimulation at three time points (Data S2-1). See STAR Methods for more details. (B) PCA of unenriched proteomic data from n = 6 donors. Protein ratio values were log_2_ transformed and only proteins quantified in all experiments (3,615) were used. The five highest and five lowest loadings from PC1 and PC2 were plotted. (C) GSEA of PCA loading scores against the Gene Ontology Biological Process database (Data S2-2). Top 15 gene sets based on normalized enrichment scores (NES) are plotted for PC1 (left) and PC2 (right). (D-E) Log_2_ fold change of protein expression (x-axis) and RNA expression (y-axis) between D8C and D8A (D) and D8C and D2 (E) T cells. Comparison restricted to genes quantified in both proteomic and transcriptomic datasets were used for the comparison (Data S2-3). Pearson’s correlation coefficient and regression line are shown. Dashed lines represent cutoffs of fold-change > 1.5. Transcriptomic data from n = 3 donors and proteomic data from n = 6 donors. (F) Volcano plot showing log_2_ fold changes of protein expression between D8C and D2 T cells. Genes related to nucleotide metabolism, redox regulation, and cell cycle are labeled based on Gene Ontology Biological Process annotation (STAR Methods). Dashed lines represent cutoffs of p-value < 0.05 and fold-change > 1.5. Data are from n = 6 donors. Filled number labels indicate number of proteins within each group passing p-value and fold-change cutoffs. (G-H) Volcano plots showing log_2_ fold changes of protein expression between D8C and D2 T cells. Mitochondrial, respiratory chain subunits I-V, and peroxisomal proteins are labeled based on Gene Ontology Cellular Component annotation (STAR Methods). Dashed lines represent cutoffs of p-value < 0.05 and fold-change > 1.5. Data are from n = 6 donors. Empty number labels indicate number of proteins within each group changing up or down regardless of magnitude. (I) Quantification of oxidative stress markers in human CD8^+^ T cells by flow cytometry using two ROS-reactive dyes: mitochondrial superoxide indicator (mitoSOX) and dihydroethidium (DHE; pan-cellular superoxides). Statistical comparison by one-way ANOVA with Šídák’s multiple comparisons test (ns, p > 0.05; * p < 0.05; *** p < 0.001; **** p < 0.0001). Donor-matched conditions are indicated by differently shaped data points. Data are from n = 5 donors. (J) Quantification of oxidative stress markers in mouse CD8^+^ T cells by flow cytometry using mitoSOX and DHE dyes^3^. Statistical comparison by one-way ANOVA (MitoSOX) and repeated measures mixed-effects analysis (DHE) with Šídák’s multiple comparisons test (ns p > 0.05; * p < 0.05). Subject-matched conditions are indicated by differently shaped data points. Data are from n = 2 mice. (K) Calculated mitochondrial and glycolytic ATP production based on extracellular flux analysis of D2 (gray), D8A (purple), and D8C (orange) human T cells. Statistical comparison by one-way repeated measures ANOVA with Tukey’s multiple comparisons test (ns p > 0.05; * p < 0.05; ** p < 0.01). Donor-matched conditions are indicated by differently shaped data points; data are presented as mean ± SEM. Data are from n = 4 donors.

We performed complementary mass spectrometry-based proteomics experiments to examine protein-level changes and their correlation with gene expression assessed by RNA-sequencing during persistent TCR stimulation (Figure 2A; Data S2-1). Using isobaric tandem mass tag (TMT) multiplexing and normalization by protein content, we directly compared protein abundances in activated, acutely, and chronically stimulated T cells at early (D4) and late (D8) timepoints. PCA revealed clear separation among activated (D2), acutely (D8A), and chronically stimulated (D8C) T cell populations after 8 days in culture, with partial separation observed at earlier timepoints (D4A and D4C) (Figure 2B). Gene ontology (GO) enrichment analysis of proteins contributing to the first two principal components revealed TCR-proximal signal transduction components and actin cytoskeletal components (PC1) and proteins involved in ribosomal synthesis and mitochondrial electron transport (PC2) (Figure 2C; Data S2-2). Hallmark exhaustion markers were consistently upregulated at both transcriptomic and proteomic levels in chronically stimulated cells, including transcription factors (EGR2, NR4A3)^30,31^, inhibitory checkpoint receptors (LAG3, CTLA4), and cytotoxic effectors (GZMB) (Figures 2D, S2A).

However, a substantial number of proteins exhibited changes only at the proteomic level, suggesting the existence of post-transcriptional regulatory mechanisms in T cell dysfunction (Figures 2E, S2B)^32,33^. Among these, were proteins involved in mitochondrial ROS detoxification (GPX1, GPX4), peroxisomal function (PXMP2), and cell-cycle progression (CDKN2A/p16^34^). Some of these changes were also observed in acute T cells (Figure S2B). Broadly characterizing these pathways, we found that chronically stimulated T cells displayed upregulation of many key cell cycle-related proteins (CHEK2, CDKN2A, CDK6), while CDK4, a major regulator of the G1/S transition^35^, was downregulated (Figure 2F). Additionally, proteins involved in nucleotide metabolism (AK4) and breakdown (CD38^36,37^, ENTPD1/CD39^38^) were also upregulated, consistent with previous reports implicating some of these enzymes in T cell exhaustion (Figure 2F). Some of these changes were already present at the earlier timepoint (D4C), suggesting an early onset of this phenotype (Figure S2C). Notably, while we observed similar changes in the regulators of G1/S transition in acute T cells (CDKN2A, CDK4), fewer cell-cycle genes were upregulated in this condition, suggesting potential differences in their regulation (Figure S2D).

### Differences in mitochondrial stress response between human and mouse T cells.

Our observation of decreased mitochondrial and redox-associated proteins in chronically stimulated cells (Figure 2F) led us to more comprehensively interrogate expression of these proteins with and without chronic TCR stimulation. Acute cells (D8A) showed a broad downregulation of mitochondrial proteins compared to activated D2 cells, consistent with reduced metabolic demand following T cell activation (Figure S2E). In contrast, differential abundance of mitochondrial proteins was heterogeneous in chronically stimulated D8C cells compared with D2, suggesting more specific changes in mitochondrial function beyond bioenergetic demand (Figure 2G). In line with the results of pathway analysis of PCA loadings, we observed decreased expression of multiple electron transport chain (ETC) components in chronically stimulated cells, including complexes I-IV, but not complex V (ATP synthase) (Figure 2G).

Additionally, we found that proteins localized to the peroxisome were increased in chronically stimulated cells, but not in acute cells, compared with activated cells (Figures 2H, S2F). Peroxisomes have well established roles in lipid synthesis and metabolism as well as NADH and redox homeostasis^39,40^. Taken together with the reduced expression of both mitochondrial ETC proteins and mitochondrial ROS detoxifying enzymes, the increased expression of peroxisome proteins suggested alterations in redox homeostasis during chronic TCR stimulation.

Alterations in redox homeostasis have been previously noted in chronically stimulated mouse T cells, which are sensitive to oxidative stress and require the reducing agent beta-mercaptoethanol (BME) in growth media to support proliferation. This redox sensitivity is exacerbated by chronic TCR stimulation, leading to accumulation of mitochondrial ROS and subsequent mitochondrial dysfunction, which can be reversed by the antioxidant *N*-acetylcysteine (NAC)^4^. While we observed a modest but significant increase in total cellular superoxide content in chronically stimulated versus acute human T cells at D8, we did not observe a parallel increase in the mitochondria-specific redox-sensitive dye MitoSOX (Figures 2I, S2G, S2H). Instead, we found that steady state levels of MitoSOX and DHE oxidation were highest after 2 days of T cell activation and gradually declined during both subsequent expansion to an acute state and chronic TCR stimulation. In contrast, chronically activated mouse T cells displayed a previously described^4^ increase in both cellular and mitochondrial markers of redox stress (Figure 2J). In combination with the observed decrease in expression of both ETC proteins and ROS detoxification proteins observed during chronic TCR stimulation, these results indicate that the bioenergetic demand of chronic TCR stimulation does not result in mitochondrial ROS accumulation in human T cells in this experimental paradigm.

To validate the impact of chronic TCR stimulation on mitochondrial oxidative phosphorylation in human T cells, we performed extracellular flux analysis on human T cells following either 2 days of T cell activation or after an additional 6 days of expansion in cytokine alone or with the addition of persistent TCR stimulation. Similar to mouse T cells, chronic antigen stimulation led to reduced mitochondrial oxygen consumption and mitochondrial ATP production compared to D2 cells (Figure S2I). In contrast, D8C cells maintained a higher level of extracellular acidification and glycolytic ATP production compared with D8A cells, indicating that suppressed mitochondrial oxidative phosphorylation in D8C occurs despite ongoing high levels of ATP demand (Figure S2J). Taken together, these results are consistent with reduced mitochondrial oxidative phosphorylation and ATP production during chronic TCR stimulation in the absence of a substantial accumulation of total cellular ROS (Figures 2K, S2K).

### Functional proteomics to broadly characterize cysteine reactivity across T cell states.

The combination of our observed extracellular flux analysis and intracellular ROS measurements indicated that, in contrast to murine T cells, chronically stimulated human T cells limit mitochondrial NADH oxidation to prevent accumulation of mitochondrial ROS but at the cost of reduced mitochondrial ATP production and nucleotide synthesis. How these and other metabolic alterations contribute to the functional defects observed in chronically stimulated human T cells is not clear, highlighting the need for complementary platforms to achieve an integrated characterization of functional, proteomic, and metabolomic changes underlying primary human T cell dysfunction following TCR stimulation.

**Figure 3.**
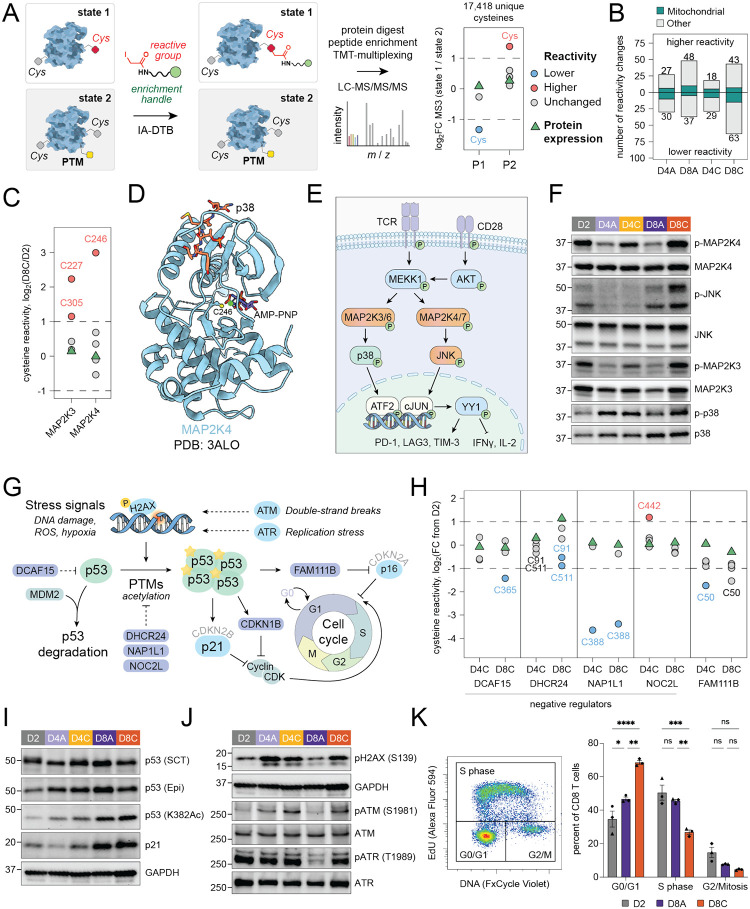
Reactivity profiling as a complementary platform for characterization of post-translational changes in T cell dysfunction. (A) Schematic of reactivity profiling experiments using a cysteine-reactive chemical probe, iodoacetamide-desthiobiotin (IA-DTB) (Data S2-4). PTM – post-translational modification. See STAR Methods for more details. (B) Bar graph of reactivity changes across cysteine reactivity profiling in n = 5 donors. Mitochondrial proteins (based on Gene Ontology annotation) are shown in teal. Higher/lower reactivity was determined by comparison to whole proteome or median cysteine reactivity. (C) Cysteine reactivity log_2_ fold change values between D8C and D2 T cells for MAP2K3 and MAP2K4. Protein expression log_2_ fold change values are shown as green triangles. Cysteine reactivity data is from n = 5 donors and protein expression data is from n = 6 donors. (D) Crystal structure of autoinhibited non-phosphorylated MAP2K4 in complex with AMP-PNP and p38 peptide (PDB: 3ALO^4^) with differentially reactive cysteine C246 labeled near AMP-binding site. (E) Schematic of MAPK pathways driven by chronic αCD3/αCD28 stimulation. Proteins with more phosphorylation in D8C than in D2 T cells are shown in orange. (F) Western blot analysis of phosphorylation levels of MAP2K4 (Ser257), JNK (Thr183/Tyr185), MAP2K3 (Ser189), and p38 (Thr180/Tyr182) relative to protein expression. (G) Schematic of DNA damage response and p53 pathways. Proteins with cysteine reactivity changes are shown in periwinkle. (H) Cysteine reactivity log_2_ fold change values in D4C and D8C T cells relative to D2 cells for proteins in the p53 pathway. Protein expression log_2_ fold change values are shown as green triangles. Cysteine reactivity data is from n = 5 donors and protein expression data is from n = 6 donors. (I) Western blot analysis of protein expression of p53 signaling pathway. Expression of p53 isoforms was verified using two different antibodies from Santa Cruz Biotechnology (DO-1) and EpiCypher. Calculated molecular weights of p53 canonical isoform and beta-isoform are 53 and 47 kDa, respectively. GAPDH included as loading control for top p53 membrane only. (J) Activation of DNA damage and replication stress pathways. Western blot analysis of protein expression levels of pH2AX (S139) and phosphorylation of ATM (Ser1981) and ATR (Thr1989). (K) Analysis of cell cycle progression in D2, D8A, and D8C T cells using EdU incorporation assay (30-minute incubation). Bar graph showing percentage of cells in G0/G1, S, and G2/M phases in each condition. Statistical comparison by two-way repeated measures ANOVA with Tukey’s multiple comparisons test of D2 versus D8A and D8C conditions (ns, p > 0.05; * p < 0.05; ** p < 0.01; *** p < 0.001; **** p < 0.0001). Donor-matched conditions are indicated by differently shaped data points. Representative gating plot for flow cytometry is shown.

We recently developed a chemical proteomic platform termed TMT-ABPP, which uses broadly reactive iodoacetamide probes to globally assess cell state-dependent changes in cysteine reactivity^24,25^. This method offers a new approach to characterize structural and functional proteomic landscapes across different T cell states, enabled by sample multiplexing using tandem mass tags (TMT). Cysteine is a highly nucleophilic amino acid that exhibits reactivity towards electrophilic chemical probes. Cysteine reactivity can be influenced by multiple factors, including direct and indirect post-translational modifications and protein interaction networks with proteins, nucleic acids, and metabolites^25,41^. We hypothesized that TMT-ABPP could provide a global strategy to identify post-translationally regulated proteins critical to T cell dysfunction but overlooked by traditional gene and protein expression analyses. Therefore, we mapped cysteine reactivity changes in activated (D2), acutely (D4A, D8A), and chronically stimulated (D4C, D8C) T cells using the same 10-plex TMT layout as the unenriched proteomics experiments. Cell lysates were treated with a broad-spectrum cysteine-reactive iodoacetamide-desthiobiotin (IA-DTB) probe, followed by trypsin and Lys-C digestion. Desthiobiotinylated cysteine-containing peptides were enriched using streptavidin beads, labeled with TMT reagents for sample multiplexing, combined, and analyzed via LC/LC-MS/MS/MS (Figure 3A). We identified reactivity changes using a cutoff of >2-fold signal intensity differences normalized to activated cells in proteins with at least two quantified cysteine-containing peptides or with a single quantified peptide and matching unenriched proteomics data (Figures S3A, S3B; Data S2-4, S2–5).

Reactivity profiling revealed more than 150 reactivity changes across different conditions, with the largest number observed in chronically stimulated D8C cells (Figure 3B). Our analysis highlighted reactivity changes in diverse protein classes relevant to T cell biology and several related to cell metabolism, including protein translation, actin binding, lipid biosynthesis, ETC activity, protein homeostasis, redox regulation, and stress response (Figures 3C, S3C). Among these, we observed state-dependent reactivity differences in stress-associated kinases MAP2K4 and MAP2K3, which are upstream of JNK and p38 stress-induced pathways (Figures 3C–3E, S3D). These cysteines, MAP2K4_C246 and MAP2K3_C227, are located in the nucleotide-binding domains of these kinases (Figure 3D)^42^. MAP2K4 has been previously shown to exhibit phosphorylation- and nucleotide binding-dependent reactivity changes in mitotic HeLa cells that correlate with its activation^41^. We confirmed activation of the MAP2K4-JNK and MAP2K3-p38 pathways in chronically stimulated (D8C), but not acute (D8A), T cells by Western blot phosphorylation analysis (Figures 3F, S3E). Indeed, it was previously shown that chronic T cell stimulation leads to upregulation of the p38 stress pathway, contributing to a dysfunctional phenotype through the YY1 transcription factor^43^. Conversely, inhibition of p38α enhances T cell expansion, expression of stemness markers, redox balance, and the antitumor efficacy of T cell-based immunotherapy^44^. These findings highlight the potential of TMT-ABPP to identify therapeutically relevant functional changes in protein landscapes, independent of gene or protein expression.

### DNA damage response and p53 pathway in chronically stimulated T cells.

Stress-induced mitogen activated protein kinase (MAPK) signaling pathways can lead to activation of p53, a multifunctional transcription factor that regulates cell cycle, DNA damage response, apoptosis, and ferroptosis (Figure 3G)^45–47^. Our reactivity profiling revealed significant changes in several negative regulators of p53, including DCAF15^48^, NOC2L^49^, NAP1L1^50^, and DHCR24^51,52^, suggesting that they might act as a negative feedback mechanism to suppress p53 pathway activation during chronic stimulation (Figure 3H). NAP1L1 and NOC2L suppress p53 acetylation through different mechanisms^49,50^. The reactivity change in NAP1L1 was observed at cysteine 388 within its C-terminal CaaX motif, a known farnesylation site that can influence NAP1L1 subcellular localization and interactions^53^. DHCR24 suppresses p53 activation by both stabilizing its interaction with MDM2 and preventing acetylation^51^. DHCR24 exhibited increased expression in chronically stimulated cells, along with two distinct reactivity changes mapped to peptides containing a predicted and a validated functional phosphorylation sites (DHCR24_C91^54^ and DHCR24_C511^55^, respectively; Figure 3H). Phosphorylation of DHCR24_Y507 increases its interaction with p53, reducing p53 transcriptional activity^51^. Supporting this, we detected increased expression of DHCR24 by Western blot analysis and a distinct shift in DHCR24 band migration in D8C cells (Figure S3F).

To explore the activation status of p53, we evaluated p53 protein expression, p53 K382 acetylation levels, and expression of p21, a canonical marker of p53 activation involved in cell-cycle regulation^56,57^. These analyses confirmed activation of the p53 pathway in both day 8 chronically and acutely stimulated T cells, although the total levels of p53 protein were slightly lower in chronically stimulated T cells (Figures 3I, S3G). Previous studies showed that p53 can undergo alternative splicing of exon-9β in p53, resulting in the production of the p53 beta-isoform, which contains a distinct *C-*terminal sequence and has been implicated in T cell replicative senescence^58,59,60^. However, we did not detect the alternatively spliced isoform in any of the conditions (Figure 3I).

DNA damage can be caused by replication stress from stalled replication forks. These are known to occur downstream of several factors in proliferating cells, including a nucleotide pool that is insufficient to meet the demands of rapid proliferation^61^. To test whether the metabolic consequences of chronic stimulation might cause replication stress and activation of the DNA damage response, we evaluated activation of ATR and ATM, as well as expression of γH2AX (pSer139) – a sensitive marker of DNA double-strand breaks – in activated (D2), acutely (D4A, D8A), and chronically stimulated (D4C, D8C) cells at early (D4) and late (D8) timepoints (Figures 3J, S3H, S3I)^45^. We observed activation of replication stress kinase ATR in both activated and chronically stimulated T cells, suggesting that the nucleotide pools may be insufficient to meet cellular demand in these conditions. We also detected ATM activation at the D4 timepoint and a significant upregulation of γH2AX (pSer139), which was greater in D4A cells (Figures 3J, S3H, S3I). Notably, γH2AX (pSer139) decreased in both acutely and chronically stimulated D8 cells. Rapid proliferation of T cells during T cell activation can cause DNA breaks and activate the DNA damage response via the ATM-CHK2-p53 pathway^62^. In addition, T cell activation increases ROS production^63^, which orchestrates downstream signaling pathways and, over time, can lead to DNA damage. Consistent with this, we detected activation of the DNA damage response two days after initial stimulation (D4A, D4C), with a stronger response in cells that have been stimulated once. This was accompanied by activation of the p53 pathway. Our flow cytometry data suggests that transient activation-induced superoxide accumulation in D2 cells gradually declines in both acutely and chronically stimulated T cells, reducing the need for ongoing DNA damage repair. In line with this observation, we detected lower levels of γH2AX (pSer139) in D8 cells compared to D4 cells.

We identified an additional reactivity change in protease FAM111B (C50), a downstream component of the p53 pathway involved in degradation of CDKN2A/p16 protein (Figures 3G, 3H)^64,65^. In chronically stimulated cells, a peptide containing predicted but functionally unannotated phosphorylation sites at serine and threonine residues (K.CSSTFK.L)^54^ exhibited a decrease in signal intensity, suggesting that the observed reactivity change may be attributed to direct phosphorylation of FAM111B. Furthermore, CDKN2A/p16 protein expression was elevated in both acutely and chronically stimulated T cells compared to activated T cells, which correlated with increased gene expression in D8A but not D8C cells (Figures 2E, S2B). These findings suggest potential involvement of FAM111B and the p53 pathway in the post-transcriptional regulation of p16 levels following chronic T cell stimulation. CDKN2A/p16 is involved in cell-cycle regulation through inhibition of key cell-cycle kinases CDK4 and CDK6. To examine the impact of chronic stimulation on cell-cycle progression, we performed an EdU incorporation assay with flow cytometry-based analysis. As anticipated, chronically stimulated T cells showed accumulation in the G0/G1 phase (Figure 3K), consistent with the activation of the p21 and p16 pathways. Notably, we also observed a reactivity change in CDKN1B/p27 (C29), an important cell-cycle kinase that also contributes to inhibition of progression from G1 to S phase (Figure S3J). The reactivity change in CDKN1B/p27 is located at the protein-protein interaction surface with cyclin D1 in the p27-cyclin D1-CDK4 complex, and CDK4 was one of the few cell cycle proteins decreased in expression following chronic stimulation, suggesting that the reactivity change might be due to changes in p27 interactomes (Figure S3J).

### Chronic stimulation leads to nucleotide-dependent reactivity changes in mitochondrial proteins.

**Figure 4.**
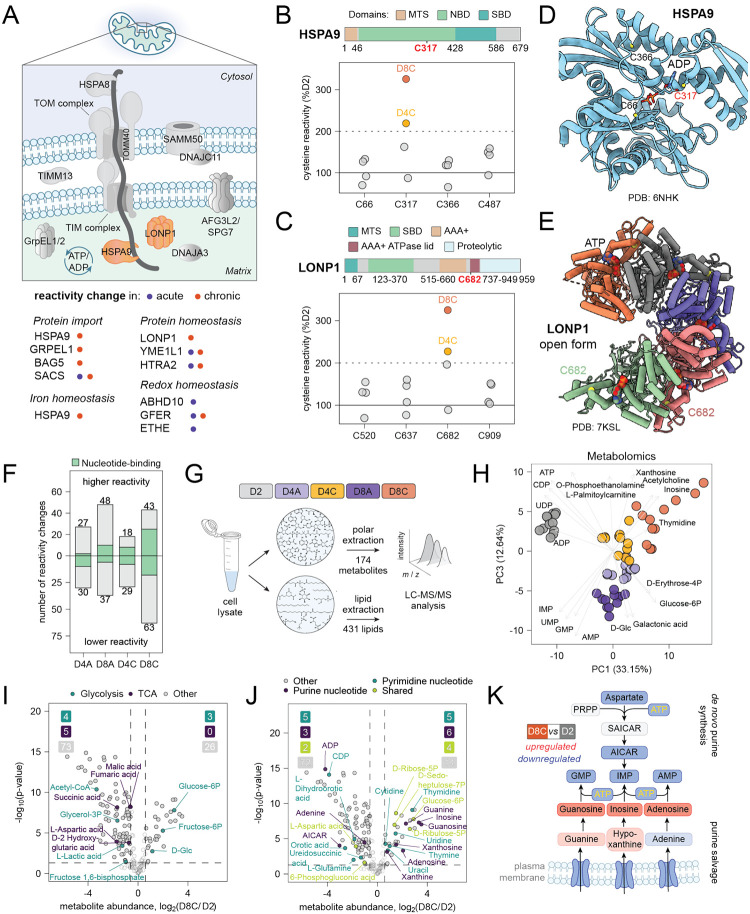
Cysteine reactivity chemical proteomics and metabolic profiling reveal dysfunction of mitochondrial protein homeostasis and *de novo* purine synthesis in chronically stimulated T cells. (A) Schematic overview of mitochondrial protein import and degradation pathways, including HSPA9 and LONP1. Proteins with cysteine reactivity changes in T cells cultured with or without chronic stimulation are marked with orange and purple dots, respectively. (B-C) Cysteine reactivity of HSPA9 and LONP1 as a percentage of D2 reactivity level. All quantified peptides for both proteins are shown. Conditions with greater than 200% D2 cysteine reactivity are labeled. Protein domain schematics are shown with differentially reactive cysteines labeled in red. Data are from n = 5 donors. MTS – mitochondrial targeting sequence, NBD – nucleotide-binding domain, SBD – substrate-binding domain. (D) Crystal structure of HSPA9 in ADP-bound state (PDB: 6NHK^5^) with quantified cysteines C66 and C366 labeled in black, and differentially reactive C317 labeled in red. (E) Electron microscopy structure of the open form of LONP1 (PDB: 7KSL^6^) with labeling of differentially reactive C682. (F) Bar graph of reactivity changes across cysteine reactivity profiling in n = 5 donors. Nucleotide-binding proteins (based on Gene Ontology annotation) are shown in green. Higher/lower reactivity was determined by comparison to whole proteome or median cysteine reactivity. (G) Schematic showing metabolomic and lipidomic profiling experiment design (Data S3-1, S3–2). See STAR Methods for more details. (H) PCA of metabolomics data from n = 4 donors. Signal intensity values were log_2_ transformed and only metabolites quantified in all replicates were used. The top and bottom five loadings from PC1 and PC3 are shown with grey arrows. (I-J) Volcano plots showing log_2_ fold changes of metabolite abundances between D8C and D2 T cells. Metabolites are colored based on manually curated pathway lists retrieved from KEGG. Dashed lines represent cutoffs of p-value < 0.05 and fold-change > 1.5. Data are from n = 4 donors. (K) Schematic of purine *de novo* synthesis and salvage pathways. Metabolites are colored according to fold change between D8C and D2 T cells.

Our proteomic, flow cytometry, and functional extracellular flux analysis data suggested that primary human T cells might leverage distinct regulatory pathways to limit ROS accumulation during chronic activation, resulting in attenuated mitochondrial ETC activity, and reduced mitochondrial ATP synthesis. The specific molecular consequences of these metabolic alterations during chronic TCR stimulation remain poorly understood. A substantial fraction of reactivity changes identified in our study were within mitochondrial proteins (Figure 4A). These included reactivity changes in proteins involved in mitochondrial protein import (HSPA9_C317, GRPEL1_C124), protein homeostasis (LONP1_C682, YME1L_C143, HTRA2_C71), ETC complex I (NDUFV1_C206, NDUFB7_C80), and redox regulation (ABHD10_C15, GFER_C188). Notably, increased cysteine reactivity in a number of proteins, including HSPA9 and LONP1 (Figures 4B, 4C), could not be explained by changes in protein expression or direct post-translational modifications at cysteine (e.g., oxidation). This suggests additional biochemical changes in mitochondria that affect the accessibility or nucleophilicity of specific cysteine residues. Structural analyses of HSPA9 and LONP1 (Figures 4D^66^, 4E^67^) revealed that the observed cysteine reactivity changes could be associated with the decreased ATP availability measured in D8C cells by extracellular flux analysis (Figures 2K, S2K). Notably, only the cysteine facing the nucleotide-binding pocket of HSPA9 exhibited altered reactivity (Figures 4B, 4D). Similarly, LONP1, an ATP-dependent protease that forms hexameric complexes for degradation of oxidatively damaged or misfolded proteins^67^, showed increased reactivity of a cysteine that is more exposed and accessible for reactions with electrophilic probes in its substrate-free, open form (Figure 4E).

### Metabolic profiling highlights reduced mitochondrial NAD^+^ regeneration, increased non-oxidative TCA cycle metabolism and activation of nucleotide salvage in chronically stimulated T cells.

Global analysis of identified reactivity changes revealed an enrichment of nucleotide-binding proteins under chronically stimulated conditions (D4C, D8C; Figure 4F), leading us to hypothesize that these reactivity changes were a result of compromised mitochondrial ATP generation in chronically stimulated T cells. To test this hypothesis and further characterize the metabolic phenotypes of activated, acutely, and chronically stimulated human T cells, we performed unbiased steady-state metabolomic analysis for polar metabolites and lipid species (Figure 4G; Data S3-1, S3–2). As D2, D8A, and D8C cells have different sizes (Figure S1B), we used mean cell volume-based normalization for all metabolomics data. PCA based on steady-state abundances of the 156 targeted polar metabolites quantified in all replicates showed that T cells could be reproducibly separated based on polar metabolite abundance alone (Figure 4H). Interestingly, nucleotides were the strongest drivers of separation between D2, D8A, and D8C cells, with nucleotide di- and tri-phosphates accumulating in D2 cells and nucleosides accumulating in D8C cells (Figures 4H, S4A). Indeed, recent studies using mouse models of chronic infection and cancer have suggested that dysregulation of *de novo* pyrimidine and purine synthesis may contribute to T cell dysfunction^68–70^.

Analysis of differentially abundant metabolites showed that compared with D2 cells, D8A cells exhibited broadly decreased abundances across all polar metabolite classes, including nucleotides, amino acids, and carbohydrates (Figure S4A, Data S3-1). These results are consistent with the reduced metabolic demand that characterizes a transition to quiescence following initial activation. Similarly, D8C cells exhibited a substantial decrease in mitochondrial TCA cycle metabolites (malate, fumarate, oxoglutarate, succinate), as well as mitochondrial precursors of nucleotide biosynthesis (dihydroorotate, aspartate). However, in contrast to D8A cells, D8C cells exhibited an accumulation of several upstream glycolytic intermediates (glucose, glucose-6P, fructose-6P) and metabolites reflective of nucleotide salvage (adenosine, xanthosine, uridine, guanosine) (Figures 4I, 4J), which is known to be induced under conditions of reduced mitochondrial ETC function^71^. These findings are consistent with reduced engagement of mitochondrial oxidative metabolism in chronically stimulated T cells. As both *de novo* synthesis of purines and pyrimidines depend on mitochondrial ATP as well as oxidative synthesis of aspartate^72,73^, activation of nucleotide salvage is also consistent with reduced ETC engagement in chronically stimulated cells (Figure 4K). Of note, we also observed a reactivity change in phosphoribosyl pyrophosphate amidotransferase (PPAT), a key enzyme that catalyzes the conversion of 5-phosphoribosyl-1-pyrophosphate (PRPP) into 5-phosphoribosyl-1-amine (PRA), the first committed step of *de novo* purine synthesis pathway (Figures S4B, S4C). PPAT binds its direct substrate, PRPP, as well as AMP and GMP - end products of the *de novo* purine synthesis pathway known to inhibit PPAT (Figure S4D)^74^. The cysteine exhibiting the observed reactivity change (C348) is located close to the allosteric pocket that binds GMP (Figure S4D), suggesting that this reactivity change detects allosteric enzyme regulation by altered metabolite abundance.

Both purine and pyrimidine nucleotide synthesis require ATP as well as precursor metabolites generated as part of mitochondrial metabolism, leading us to ask whether TCA cycle metabolism was globally altered in D8C cells. To answer this question, we performed stable isotope tracing of D2, D8A, and D8C cells cultured in the presence of universally ^13^C-labeled glucose or glutamine for 4 hours (Figures S4E, S4F; Data S3-3, S3–4). Analysis of glucose tracing showed that steady-state labeling of glycolytic intermediates by glucose was unimpaired in D8C cells, indicating that glucose uptake and glycolytic metabolism was unimpeded (Figure S4G). Steady-state labeling of glutamine as well as proximal glutamine metabolites glutamate and alpha-ketoglutarate (α-KG) was increased in D8C cells, indicating that both uptake and initial metabolism of glucose and glutamine was unimpaired by chronic TCR stimulation (Figure S4H). However, we observed a substantial decrease in downstream oxidative metabolism of either glucose or glutamine within the TCA cycle, as demonstrated by a significant decrease in m+2 labeling of fumarate, malate, and aspartate by ^13^C-glucose and a significant decrease in m+4 labeling of aspartate by ^13^C-glutamine. The mitochondrial TCA cycle consists of both oxidative (NAD^+^-dependent) and non-oxidative enzymatic reactions; the decrease in TCA cycle labeling by both ^13^C-glucose and ^13^C-glutamine was most potent for NAD^+^-dependent reactions catalyzed by IDH3, OGDH, and MDH2 (Figures S4I, S4J). This suggested that the metabolic phenotype observed in chronically stimulated T cells is at least partially driven by decreased engagement of ETC-dependent mitochondrial NAD^+^ regeneration.

We also observed a substantial increase in non-oxidative metabolism of glutamine in D8C cells to generate proline, which has been shown to limit mitochondrial redox stress (Figure S4H)^75–77^. Moreover, we observed a decrease in the oxidative metabolism of both glucose- and glutamine-derived citrate in D8C versus D8A cells (Figures S4I, S4J). Citrate is an essential precursor for the *de novo* synthesis of fatty acids^78–80^, which can limit mitochondrial ROS by serving as an electron acceptor^81^. To test whether chronic TCR stimulation altered intracellular lipid pools, we performed whole-cell lipidomic profiling on D2, D8A, and D8C cells (Figures S5A–S5C, Data S3-2). PCA based on steady-state abundances of the 402 targeted lipids quantified in all replicates showed that T cells were also well separated based on lipid metabolite abundance alone (Figure S5A). Accumulation of ether phospholipids was a primary driver of separation between D8 and D2 cells, whereas triglycerides drove the separation between D8A and D8C cells. Analysis of differentially abundant lipids between D8C and D2 cells confirmed an accumulation of triglycerides and ether phospholipids, in contrast to D8A cells, which showed much lower accumulation of triglyceride species (Figures S5B, S5C). Both ether phospholipids and triglycerides act as electron acceptors and play established roles in antioxidant defense^81,82^. In combination with our proteomic data showing an increase in accumulation of proteins from peroxisomes, which are required for ether phospholipid synthesis, these data indicate that chronically stimulated T cells might limit donation of electrons to the mitochondrial ETC by shunting NADH towards alternative electron sinks, including proline and lipid synthesis (Figure S5D).

### Proteome-wide effects of nucleotide imbalance in chronically stimulated T cells.

**Figure 5.**
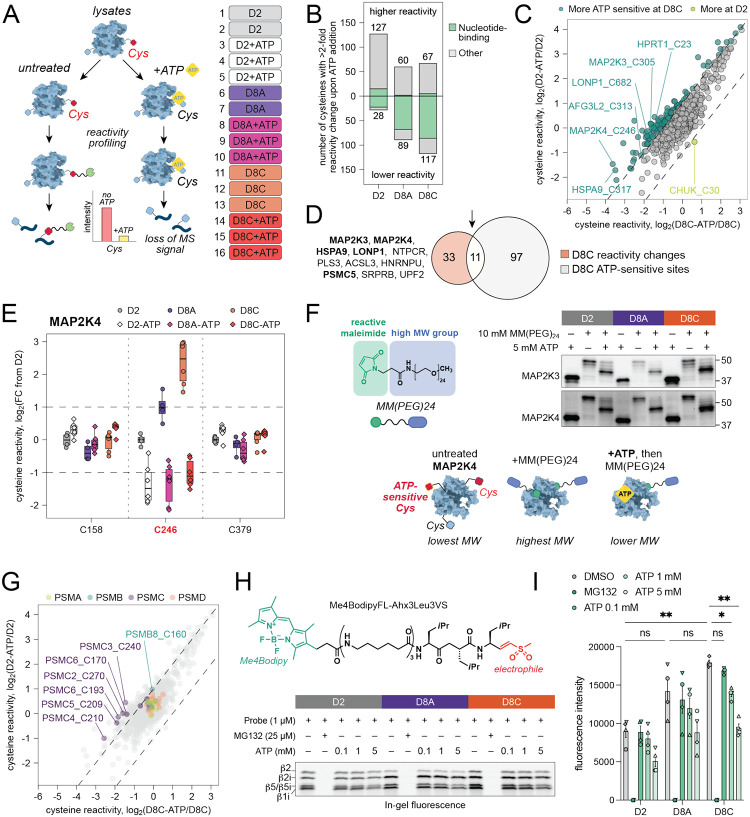
A function-first approach for characterization of ATP-sensitive reactivity changes in different T cell states. (A) Schematic of ATP add-back experiment and diagram of TMT 16-plex channels (Data S2-6). See STAR Methods for more details. (B) Quantification of cysteines with two-fold difference in cysteine reactivity between control and ATP add-back conditions in D2, D8A, and D8C T cells. Nucleotide-binding proteins (green) are labeled based on Gene Ontology annotation. (C) Scatter plot of log_2_ fold changes between control and ATP add-back conditions in D8C (x-axis) and D2 (y-axis) T cells. Dashed lines indicate a cutoff for ratio of fold change between D8C and D2 T cells of >2. (D) Venn diagram of reactivity changes and ATP-sensitive sites in D8C cells. Only cysteines quantified in both datasets are shown. (E) Boxplot of log_2_ fold changes in cysteine reactivity of MAP2K4 in control (circle) and ATP add-back (diamond) conditions in D2, D8A, and D8C T cells compared to D2 control condition. Boxes mark the lower and upper quartiles, horizontal black lines mark the median value, and whiskers extend to the furthest point within 1.5x the interquartile range. (F) Validation of ATP-sensitivity of reactive cysteines in MAP2K3 and MAP2K4 using activity-based protein profiling with a high-molecular weight cysteine-reactive maleimide probe and Western blot visualization (WB-ABPP). (G) Scatter plot of log_2_ fold changes in cysteine reactivity between D8C cells with ATP add-back compared to D8C cells without ATP add-back (x-axis) and log_2_ fold change in cysteine reactivity between D2 cells with ATP add-back compared to D2 cells without ATP add-back (y-axis). Colors show proteasome subunit families PSMA, PSMB, PSMC, and PSMD. Data are from n = 2 donors analyzed across two independent cysteine reactivity profiling experiments. (H) Chemical structure of a fluorescent proteasome activity probe, Me_4_BodipyFL-Ahx_3_Leu_3_VS (top) and gel-based proteasome activity profiling of D2, D8A, and D8C T cells (bottom). Cell lysates were preincubated with 25 μM MG132 or ATP (0.1, 1 or 5 mM), followed by incubation with 1 μM Me_4_BodipyFL-Ahx_3_Leu_3_VS. (I) Quantfication of active protease subunit labeling in cell lysates of D2, D8A, and D8C T cells. Statistical comparisons by two-way repeated measures ANOVA with Šídák’s multiple comparisons test of D2 vs D8A, D2 vs D8C, and D8A vs D8C, as well as D8C + DMSO vs D8C + 0.1, 1 or 5 mM ATP (ns p>0.05; * p < 0.05; ** p < 0.01). Donor-matched conditions are indicated by differently shaped data points.

To determine whether the observed insufficiency in mitochondrial ATP synthesis was directly responsible for the observed state-dependent differences in cysteine reactivity, we conducted a “function-first” ATP add-back experiment in which T cell lysates from activated (D2), acutely (D8A), and chronically (D8C) stimulated cells were treated with 5 mM ATP for 10 min before exposure to a broadly reactive iodoacetamide probe, followed by the standard TMT-ABPP workflow (Figure 5A; Data S2-6). We anticipated that cysteines directly or allosterically affected by ATP binding would exhibit changes in reactivity. As expected, most decreased reactivity changes were observed in nucleotide-binding proteins (Figure 5B). Reactive cysteines in chronically stimulated T cells were more sensitive to ATP add-back than in activated or acute conditions (Figures 5B, 5C). In line with our hypothesis, several proteins with reactivity changes were sensitive to ATP add-back (Figure 5D). These included the stress-sensing kinases MAP2K4_C246 and MAP2K3_C305, both of which showed decreased cysteine reactivity in ATP-treated conditions (Figures 5D, 5E, S6A). We further validated T cell state-dependent ATP sensitivity of MAP2K3 and MAP2K4 cysteine reactivity by performing an in-gel ABPP experiment with a high-molecular weight maleimide-based cysteine-reactive probe, followed by Western blot visualization of MAP2K3 and MAP2K4, both of which showed probe- and ATP-dependent band shifts (Figure 5F).

As predicted through structural analyses, reactivity changes in the key proteins within mitochondrial protein import complex – HSPA9 and LONP1 – were both ATP-sensitive (Figure S6B). HSPA9 is a multifunctional chaperone, essential for maintaining mitochondrial protein quality control, Fe-S cluster biogenesis, and protection from oxidative stress^83–85^. Similarly, the mitochondrial protease LONP1 plays a critical role in mitochondrial function by degrading unfolded and oxidized proteins and facilitating mitochondrial protein folding together with HSPA9^85,86^. To assess whether LONP1 function was compromised in chronically stimulated T cells, we separated D2, D8A, D8C, D15A, and D15C cells into detergent-soluble and insoluble fractions (Figure S6C). This method enables evaluation of improperly folded or aggregated proteins, which accumulate in the insoluble pellet, and has previously been used to identify proteins affected by LONP1 knockout in 143B cancer cells, including an HSPA9 co-chaperone DNAJA3 and a broader subset of proteins^85^. Western blot analysis revealed that DNAJA3 accumulated in the detergent-insoluble fraction following chronic stimulation, indicating potential impairment of LONP1 ATPase function, which has been previously shown to be required for its solubility (Figures S6D, S6E). However, HSPA9 and NDUFA9, a complex I component sensitive to LONP1 knockout in other systems^85^, were not affected in our experimental setup (Figures S6D, S6F). These findings suggested that LONP1 ATPase function may be only partially impaired under conditions of chronic T cell stimulation.

Five out of six ATPase subunits of the 26S proteasome complex (PSMC proteins) exhibited ATP-sensitive reactivity changes, whereas non-ATPase subunits (PSMA, PSMB, and PSMD proteins) remained unaffected (Figure 5G). We leveraged existing activity-based probes targeting proteasome subunit^87^ to test proteasome activity in D2, D8A, and D8C cells, and the role of ATP concentration in regulating its function (Figures 5H, 5I). Proteasome activity was higher in chronically stimulated cells, in line with previous literature showing non-linear relationship between ATP concentrations and proteasome activity^88^ (Figures 5H, 5I). As expected, proteasome activity was decreased across all conditions following addition of additional ATP to the lysates.

We further identified ATP-sensitive reactivity changes in additional proteins involved in mitochondrial proteostasis (AFG3L2_C313, SPG7_C353, Figure S6G) and purine salvage pathway (HPRT1_C23, C106; Figures S6G, S6H)^71,89^, most of which were located within nucleotide-binding pockets. An intriguing example of an ATP-sensitive reactivity change was observed in ZAP70 kinase, a critical mediator of TCR signaling^90^. Here, an ATP-sensitive reactivity change was detected in a cysteine distant from the ATP-binding pocket (C596), but not in a cysteine directly facing ATP (C346) according to available crystal structures for both active and autoinhibited conformations of the kinase^91^, suggesting potential allosteric regulation of cysteine reactivity (Figures S6I, S6J). These findings suggest that altered ATP availability broadly contributes to the reactivity changes observed in nucleotide-binding proteins in T cells, many of which are associated with changes in protein function. Given the significant reduction in phosphorylated nucleotide metabolites observed in our metabolomic profiling of chronically stimulated T cells (Figure 4J), dysregulation of *de novo* nucleotide synthesis may have widespread effects on cellular function during chronic TCR stimulation.

### Altered nucleotide synthesis in chronically stimulated T cells contributes to the development of integrated and proteotoxic stress.

**Figure 6.**
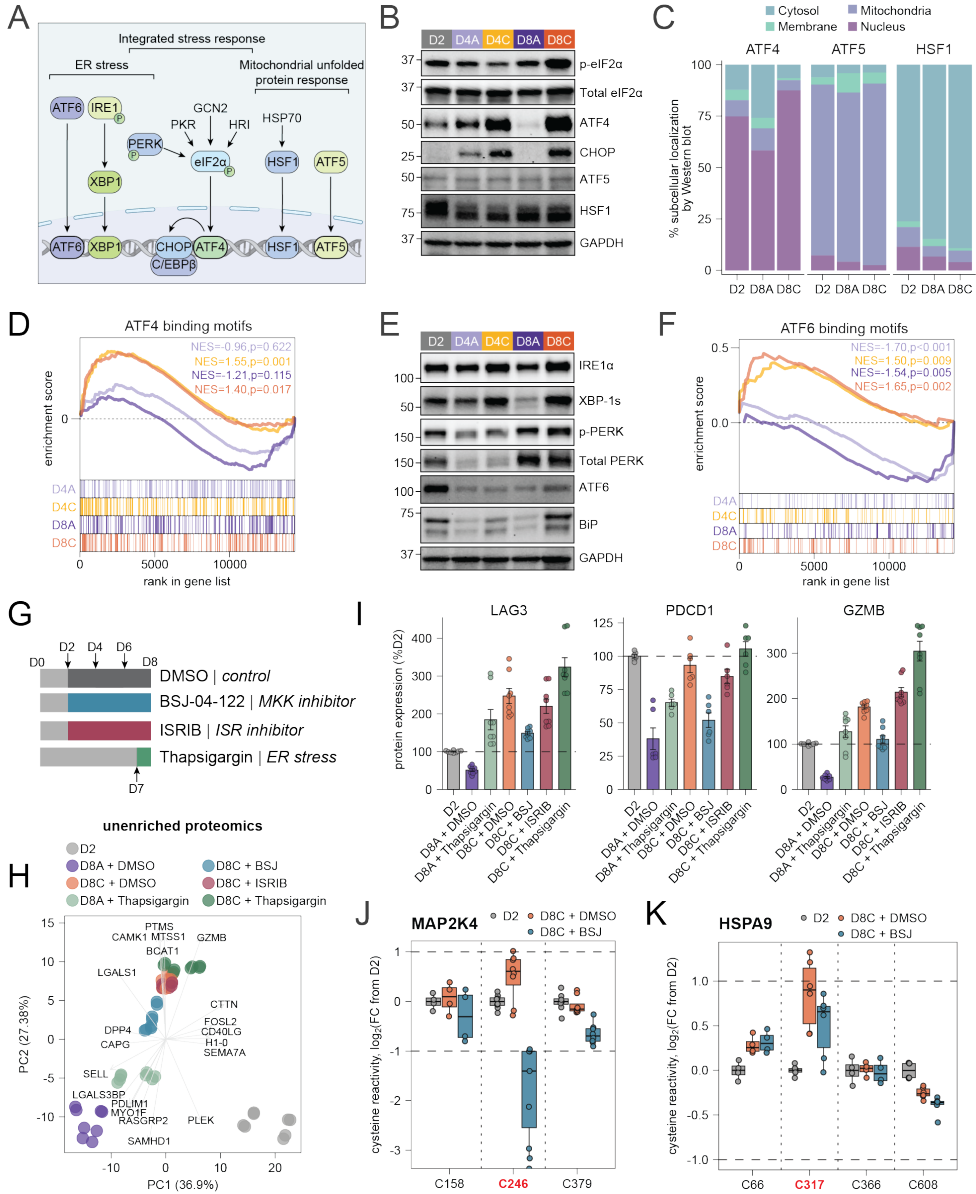
Activation of multiple parallel cellular stress responses drives chronic stimulation-dependent T cell dysfunction. (A) Schematic of mtUPR, ISR, and ER stress-response pathways. (B) Western blot analysis of protein expression in ISR (ATF4, CHOP, p-eIF2a) and mtUPR (ATF5, HSF1) pathways. GAPDH included as loading control for ATF4 membrane only. (C) Western blot quantification of subcellular localization of ATF4, ATF5, and HSF1. Data are shown as average of n = 2 donors. (D) GSEA of bulk RNA-sequencing data on the Hallmark gene set for ATF4_BINDING_MOTIFS. Each condition was compared to D2 T cells. Gene expression data are from n = 3 donors. (E) Western blot analysis of protein expression of ER stress signaling pathway members. GAPDH included as loading control for IRE1α membrane only. (F) GSEA of bulk RNA-sequencing data on Hallmark gene sets for ATF6_BINDING_MOTIFS. Each condition was compared to D2 T cells. Gene expression data are from n = 3 donors. (G) Schematic of cytokine-mediated T cell expansion with or without chronic TCR stimulation and addition of small-molecule modulators of MAPK (10 μM BSJ-04–122), ISR (200 nM ISRIB), and ER stress (25 nM Thapsigargin) pathways. (H) PCA of unenriched proteomic data with or without inhibitor treatment from n = 4 donors (Data S2-7). Protein ratio values were log_2_ transformed and only proteins quantified in all experiments (4,198) were used. The five highest and five lowest loadings from PC1 and PC2 were plotted. (I) Bar graphs showing LAG3, PDCD1, and GZMB protein expression levels in D2, D8A, and D8C T cells with or without inhibitor treatments compared to D2 T cells (% of median D2 expression). Data are presented as mean ± SEM; n = 4 donors (2 technical replicates per experimental condition). (J-K) Boxplot of log_2_ fold change in cysteine reactivity of MAP2K4 (J) and HSPA9 (K) proteins between D8C and D2 T cells with or without BSJ treatment (Data S2-8). Cysteines with reactivity changes are highlighted in red. Boxes mark the lower and upper quartiles, horizontal black lines mark the median value, and whiskers extend to the furthest point within 1.5x the interquartile range. Data are from n = 4 donors analyzed across four independent cysteine reactivity profiling experiments.

Our broad cysteine reactivity analysis of chronically stimulated T cells identified preferential reactivity changes both in protein classes related to cell metabolism as well as in protein homeostasis and stress responses. We therefore hypothesized that chronic antigen-driven impairment in mitochondrial ETC activity, ATP production, and limited accumulation of aggregated proteins would lead to activation of mitochondrial unfolded protein response (mtUPR), integrated stress response (ISR), or endoplasmic reticulum (ER) stress response pathways due to a loss of chaperone-mediated ATP-dependent protein folding (Figure 6A)^92–94^. Western blot analysis of expression (Figure 6B) and nuclear translocation of ATF5^95^ and HSF1^96^ (Figures 6C, S7A), both of which are hallmark markers of mtUPR activation, were not induced by chronic TCR stimulation, likely reflecting the lack of increased mitochondrial ROS levels in chronically stimulated human T cells.

We next looked into activation of ISR, which is known to be activated in the setting of decreased mitochondrial ATP production^97^ and which is regulated by four kinases – PERK, PKR, GCN2, and HRI^98^. To validate ISR activation, we demonstrated increased phosphorylation of eukaryotic translation initiation factor 2 alpha (eIF2α) and expression of canonical ISR markers ATF4 and CHOP by Western blot in chronically stimulated T cells (Figures 6B, S7A). Subcellular fractionation followed by Western blot analysis further confirmed nuclear localization of ATF4 and CHOP, supporting ISR activation (Figure 6C, S7B). This was consistent with the enrichment of gene expression signature for ATF4-binding elements by gene set enrichment analysis (GSEA) (Figure 6D) and upregulation of ISR-regulated genes observed by RNA-sequencing (Figure S7C). Notably, we observed ATP-sensitive reactivity changes in chronically stimulated T cells in three cysteine residues within the stalled ribosome sensor GCN1, a key regulator of GCN2 activation and ISR (Figure S7D)^99^. While GCN1 does not directly bind ATP, previous studies have shown that ATP enhances the formation of GCN1 complexes with GCN2, GCN20, and polysomes^100^. Of the three affected cysteines, two (C55, C648) are located in the polysome-binding region, while the third (C2179) resides within the GCN2-binding domain^100^.

We further confirmed activation of ER stress response pathway during chronic TCR stimulation by looking at expression of key markers, including ATF6, PERK, BiP, and the spliced form of XBP1 by Western blot (Figures 6E, S7E). GSEA of RNA-sequencing data further revealed enrichment in chronically stimulated cells of a gene expression signature associated with ATF6-binding elements in chronically stimulated cells (Figure 6F). Combined, this data suggests that the ISR and ER stress response pathways, but not mtUPR, mediate the stress response in chronically stimulated primary human T cells. This lack of mtUPR activation is consistent with decreased ATP availability rather than mitochondrial ROS being a fundamental driver of dysfunction in chronically stimulated human T cells^93,101^.

Finally, we asked whether ER stress or stress kinase activation contributed substantially to the development of dysfunctional T cell state. To address this question, we treated activated T cells (D2) with either the reported MKK4/7 inhibitor BSJ-04–122^102^ or the ISR antagonist ISRIB^103^ for 6 days following primary T cell activation (Figure 6G). We also activated ER stress by treating acutely or chronically stimulated T cells between D7 and D8 with thapsigargin^104^, which inhibits SERCA pumps and ER calcium stores leading to ER stress induction (Figures 6G, S8A). Western blot analysis confirmed that thapsigargin was sufficient to induce ATF4 stabilization in acutely stimulated T cells, while ISRIB reduced ATF4 levels in chronically stimulated T cells (Figure S8B). We also discovered that following the prolonged 6-day treatment, BSJ-04–122 significantly reduced phosphorylation and activation of both MAP2K3 and MAP2K4 as well as their downstream targets p38 and JNK (Figure S8C).

We profiled these cells using a newly developed low-input proteomics platform, which leverages SP3 bead enrichment, enabling >20-fold reduction in protein input requirements for both unenriched proteomics and reactivity profiling experiments (Figure S8D; Data S2-7, S2–8)^105^. PCA of unenriched proteomics data revealed clear separation of activated (D2), acutely stimulated (D8A), and chronically stimulated (D8C) T cells (Figure 6H). Consistent with roles for both ER stress^106^ and MAP2K4 in driving T cell exhaustion, thapsigargin-treated D8A cells had increased proteomic similarity to D8C cells compared with DMSO-treated D8A cells, whereas BSJ-04–122-treated D8C cells were more similar to D8A cells compared with DMSO-treated D8C cells (Figure 6H). Interrogation of specific proteins associated with exhaustion, including checkpoint receptors (PDCD1, LAG3, CTLA4), transcription factors (NFATC1, NR4A3, EGR2), and GZMB, showed an increase with thapsigargin treatment and a decrease with BSJ-04–122 (Figures 6I, S8E). Finally, thapsigargin was sufficient to induce expression of proteins containing both ATF4 and ATF6 binding motifs in the absence of chronic TCR stimulation, whereas BSJ-04–122 attenuated chronic TCR stimulation-driven expression of the same proteins (Figures S8F). We validated key changes by flow cytometry and observed similar changes in PD-1 and LAG-3 to those seen in our proteomic data (Figure S8G). Notably, ISRIB treatment did not produce a comparable rescue effect, as assessed by both proteomic and flow cytometric analyses.

Our reactivity profiling data further confirmed engagement of both MAP2K4_C246 and MAP2K3_C207 following BSJ-04–122 treatment, as observed by competition for their reactivity with this covalent electrophile (Figures 6J, S8H). In addition to canonical exhaustion markers, we observed partial reversal of chronic stimulation-driven cysteine reactivity changes for C317 in HSPA9 (Figure 6K). Taken together, these results are consistent with effective pharmacologic induction and reversal of T cell dysfunction by targeting ER stress and stress kinases, respectively.

### Proteomic profiling confirms stress kinase activation in *in vivo* exhausted murine T cells and primary patient TILs.

**Figure 7.**
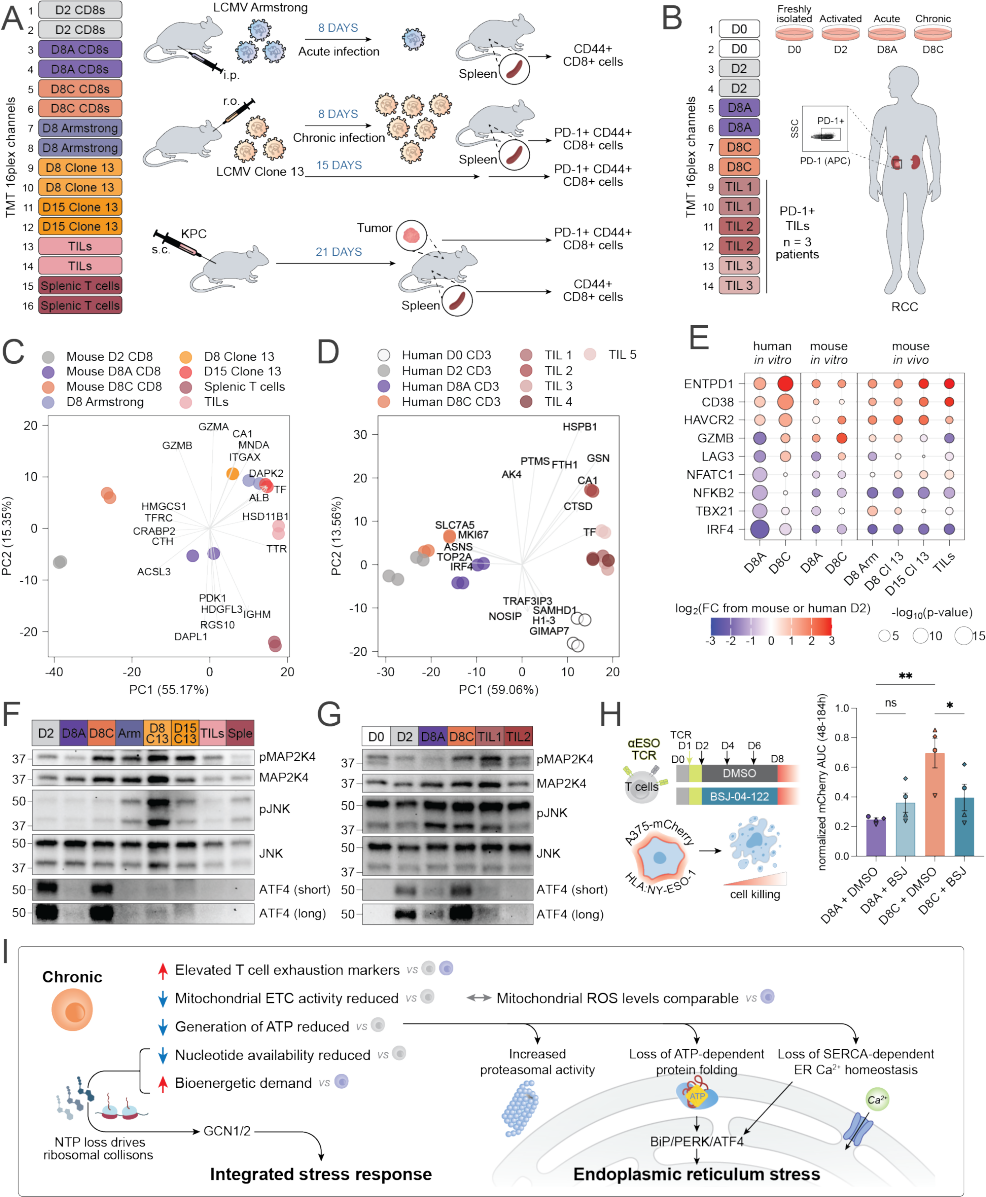
Unbiased proteomic analysis of exhausted murine and human T cells *in vivo*. (A) Schematic depicting experimental strategy to generate activated or exhausted CD8^+^ T cells from mice infected with acute or chronic strains of lymphocytic choriomeningitis virus (LCMV) infection as well as mice bearing subcutaneous KPC tumors in comparison with mouse T cells undergoing acute or chronic stimulation *in vitro*. Viably sorted CD8^+^ T cells with the indicated phenotypes were analyzed by low-input unenriched proteomics and Western blot analysis. (B) Schematic depicting the strategy to isolate human CD3^+^ T cells generated *in vitro* as described previously, as well as PD-1^+^ cells from renal cell carcinoma (RCC) patient tumors. Viable CD3^+^ T cells were sorted as shown. (C) PCA of unenriched proteomic data of *in vitro*-generated mouse D2, D8A, D8C T cells, and T cells isolated from mice bearing either LCMV infections or KPC tumors. Protein ratio values were log_2_ transformed and only proteins quantified in all experiments (4,343) were used. The five highest and five lowest loadings from PC1 and PC2 were plotted. (D) PCA of unenriched proteomic data of *in vitro*-generated human D0, D2, D8A, D8C T cells, and TILs isolated from five independent RCC patients. Protein ratio values were log_2_ transformed and only proteins quantified in all experiments (1,904) were used. The five highest and five lowest loadings from PC1 and PC2 were plotted. (E) Dot plot of log_2_FC and p-values of selected proteins detected in human and mouse T cells *in vitro* and *in vivo*. Values are relative to human or mouse D2 T cells. (F) Western blot analysis of *in vitro*-generated mouse T cells and T cells from mice bearing either LCMV infections or KPC tumors. (G) Western blot analysis of *in vitro*-generated human T cells and TILs from two independent RCC patients. (H) Schematic (left) and quantification (right) of antigen-specific cancer cell killing by D8A and D8C T cells transduced with NY-ESO-1-specific TCR and treated with DMSO or BSJ-04–122. Normalized AUC of mCherry intensity for 48–184 hours normalized to untreated D8A condition. Statistical comparison by one-way repeated measures ANOVA with Šídák’s multiple comparisons test (ns p > 0.05; * p < 0.05; ** p < 0.01). Donor-matched conditions are indicated by differently shaped data points. (I) Schematic showing molecular differences between activated, acutely, and chronically stimulated primary human T cells.

The dysfunctional T cell transcriptomic and epigenomic program, often referred to as T cell ‘exhaustion’, has classically been established in T cells from mice with chronic viral infections or from either mouse or human tumors. The proteomic landscape of mouse or human T cells undergoing chronic antigen-driven dysfunction *in vivo* have not been well described. We therefore isolated CD8^+^ T cells from the spleens of mice bearing either acute (LCMV-Armstrong) or chronic (LCMV-Clone 13) viral infections as well as from the tumors and spleens of KPC tumor-bearing mice, which we directly compared with D2, D8A, or D8C mouse T cells *in vitro* using low-input unenriched proteomics (Figure 7A, S9A; Data S2-9). In parallel, we compared CD3^+^PD-1^+^ TILs from primary renal cell carcinoma tumors together with D2, D8A, or D8C human T cells *in vitro* (Figure 7B, S9B; Data S2-10). PCA of mouse T cells revealed one principal component (PC1) which predominantly separated *in vitro* from *in vivo* conditions and a second component (PC2) which predominantly separated T cells experiencing chronic antigen stimulation *in vitro* (D8C) or antigen stimulation *in vivo* (LCMV-Arm, LCMV-Clone 13, TIL), suggesting both antigen and environmentally driven proteomic alterations *in vivo* (Figures 7C, S9C). PCA of human T cells similarly separated human T cells cultured *in vitro* from primary TILs along PC1, with a smaller contribution of PC2 separating TILs and D8C cells from D0, D2, and D8A cells (Figure 7D). These results suggest that certain proteomic changes seen in dysfunctional T cells *in vivo* may not be fully recapitulated *in vitro*. Importantly, however, key exhaustion markers were similarly regulated across both human and mouse *in vitro* and *in vivo* models (Figures 7E, S9D, S9E). A notable exception was regulation of mitochondrial and peroxisomal proteins, which showed distinct patterns from the ones observed for the human *in vitro* system, possibly indicative of distinct adaptive strategies to counter redox stress in mouse and human T cells (Figure S9F; Data S2-9).

Importantly, we observed increased phosphorylation of MAP2K4 and JNK in dysfunctional T cells from LCMV-Clone 13 infected mice, MC38 tumors, and primary human TILs from renal cell carcinoma tumors, recapitulating our findings from the *in vitro* system (Figures 7F, 7G). We asked whether pharmacologic targeting of stress pathway or stress kinase activity would impact T cell effector function during serial tumor encounter. T cells transduced with the affinity-enhanced NY-ESO-1-specific TCR 1G4-LY were acutely or chronically stimulated for 6 days in the presence of either vehicle, BSJ-04–122, or thapsigargin (18 hours) and then co-cultured with NY-ESO-1^+^ A375 melanoma cancer cells for an additional eight days, with replenishment of cancer cells every 2 days. BSJ-04–122 dramatically restored the cytotoxic capacity of chronically stimulated T cells as reflected by a significant reduction in the tumor cell abundance over eight days of co-culture (Figures 7H, S9G). In contrast, thapsigargin pre-treatment impaired tumor control by acutely activated T cells (Figure S9H).

## DISCUSSION

Chronic antigen-driven T cell dysfunction remains a significant barrier to anti-tumor immunity. In the present study, we leverage an *in vitro* platform of chronic antigen stimulation in peripheral blood human T cells from healthy donors to identify unique metabolic, proteomic, and post-translational alterations that are directly attributed to persistent TCR stimulation. This platform generates dysfunctional T cells at a time-resolved scale that enables broad metabolomic and proteomic profiling, while recapitulating many of the key features of *in vivo* T cell dysfunction, including upregulation of inhibitory immunoreceptors, expression of exhaustion-associated transcription factors, and loss of cytotoxic function. While this platform is unlikely to comprehensively capture all features of dysfunctional T cells within tumors, and particularly those features driven primarily by the tumor microenvironment, it offers a powerful and scalable experimental paradigm to identify specific proteomic and metabolomic changes driven by chronic TCR stimulation. Importantly, we validated key proteomic changes identified using our platform using conventional *in vivo* mouse T cell exhaustion platforms and primary patient TILs, supporting the utility of this approach in identifying new therapeutic targets to reverse chronic antigen-driven T cell dysfunction.

One key observation from our study is that many metabolic and proteomic hallmarks of T cells rendered dysfunctional by chronic TCR stimulation (“D8C”) are best revealed in comparison to activated T cells (“D2”) rather than acute cells that were previously activated but expanded in the absence of further antigen (“D8A”). This is critical given work from our groups and others demonstrating that T cell activation itself is associated with metabolic and proteomic rewiring. The metabolic phenotype of T cells returns to quiescence following initial activation, and as a result comparisons between T cells expanded following a single antigenic challenge and T cells encountering chronic antigen may not appropriately capture the metabolic or proteomic phenotype relevant to T cell dysfunction following chronic stimulation. For example, nucleotide pools are reduced in D8C cells compared with D2, but not D8A cells. This is a consequence of decreased metabolic demand in acute T cells and metabolic dysfunction in chronically stimulated T cells.

Our work identified, for the first time, unique metabolic features of human T cells undergoing TCR stimulation. Key amongst these features is the accumulation of mitochondrial NADH, similar to what has been observed in mouse T cells. In response to chronic stimulation, human T cells induce several adaptive mechanisms to limit mitochondrial ROS accumulation, diverting both carbon and reducing equivalents towards triglyceride, ether phospholipid, and proline biosynthesis. This is reflected in the accumulation of both neutral lipids and peroxisomal proteins (the site of ether phospholipid synthesis) during chronic TCR synthesis. Our observed decrease in protein expression of respiratory chain complexes I-IV further reflects an adaptive reduction in ETC flux during chronic TCR stimulation, thereby reducing ROS accumulation at the expense of nucleotide synthesis. The interplay between peroxisomes and other organelles, including mitochondria and ER, is well-documented^40,107–109^; however, little is known about their contribution to cellular ROS and lipid management in the context of mitochondrial dysfunction in T cells. Further investigations into the interplay between peroxisomes and mitochondria in T cells will be essential to provide a better mechanistic understanding and determine its role in ROS detoxification, lipid homeostasis, and T cell dysfunction following chronic antigen stimulation.

Our protein abundance and reactivity profiling analyses identified several changes during chronic stimulation that are directly connected to these metabolic defects, including activation of stress-associated kinases in the p38 and JNK pathways and DNA damage response. Notably, we identified and validated the MAP2K4-JNK axis as a targetable regulatory node capable of reducing the accumulation of exhaustion markers and extending T cell functional persistence and cytotoxic capacity. Activation of this pathway was orthogonally confirmed in exhausted T cells from mice bearing both chronic viral infections and tumors, as well as in primary TILs from renal cell carcinoma patients. Our results also point to broader functional consequences related to nucleotide-binding proteins, including mitochondrial protein homeostasis and proteasome activation. As a result of these and likely other changes, chronically stimulated T cells exhibit activation of both the integrated and ER stress responses. Functional follow up studies indicated that ER stress, rather than the ISR, contributes to the establishment of T cell exhaustion program, even in the absence of chronic antigen stimulation. Interestingly, the mtUPR was not activated in chronically stimulated T cells; this has been shown to require not only accumulation of unfolded or misfolded proteins but also elevated mitochondrial ROS, which we did not observe in chronically stimulated human T cells. Further mechanistic studies are necessary to elucidate the extent of mitochondrial protein homeostasis dysfunction and to determine why mtUPR activation is absent. These investigations could provide deeper insights into the ability of human T cell mitochondria to maintain protein homeostasis under chronic stress conditions.

Our study validated cysteine reactivity profiling as an unbiased approach to investigate structural and functional proteomic changes across different immune cell states. While we identified nucleotide binding as a key biochemical feature that can be read out using reactivity profiling platforms, it is important to recognize that the enrichment and identification of cysteine-containing peptides can also be influenced by other direct or indirect post-translational modifications on the same tryptic peptides, including phosphorylation, ubiquitination, and fatty acylation, among others. We demonstrated that function-first approaches, including ATP add-back TMT-ABPP experiments with cysteine-reactive probes, serve as powerful strategies for annotating biochemical cues associated with specific reactivity changes. Expanding function-first chemical proteomic platforms will deepen our understanding of the factors driving these reactivity changes.

While this study focused on stress-response pathways and mitochondrial and metabolic dysfunction, our comprehensive dataset – including transcriptional, metabolomic, and functional proteomic profiling in primary human T cells – provides a valuable foundation for future investigations. This resource can be leveraged to explore the roles of specific proteins and pathways in T cell dysfunction, including splicing factors, transcriptional regulators, chromatin remodeling proteins, and translation elongation factors, among others. The development of additional proteogenomic pipelines will further enable functional validation of select targets and identification of candidates for future mechanistic studies and chemical probe development.

### Limitations of the study

We acknowledge several potential limitations to our experimental paradigm. Our platform allowed us to identify specific metabolic and proteomic alterations that are attributable to chronic TCR stimulation. However, our proteomic analysis of *in vivo* exhausted T cells from mice and primary human TILs clearly indicates additional proteomic alterations that are environmentally encoded. Tumor-infiltrating T cells are subject to several cellular and environmental inputs *in vivo* that may contribute to their dysfunction, including antigens of varying TCR avidity, immunosuppressive stromal cells, paracrine factors including cytokines, and altered nutrient availability. We focused on the role of chronic TCR stimulation because evidence of persistent antigen stimulation is seen in tumor infiltrating T cells across tumor types. We anticipate building upon this platform to understand how additional tumor-associated features synergize with chronic TCR stimulation to activate dysfunctional programs that are tissue- and/or tumor-specific.

It is important to acknowledge that some of the proteins exhibiting reactivity changes may have functional roles beyond those described in this study. For example, NAP1L1 is a multifunctional protein with reported roles beyond p53 regulation, including nucleosome, chromatin, and microtubule assembly. Similarly, DHCR24 is a key enzyme, which participates in important cholesterol biosynthesis pathways. Cholesterol is a key lipid, which has been reported to promote T cell exhaustion within the tumor microenvironment by inducing ER stress and upregulating XBP1 expression^110^. At this stage, we cannot rule out potential involvement of these proteins in additional pathways relevant to T cell dysfunction following chronic antigen stimulation.

Our stringent cutoffs for defining reactivity changes enabled identification of robust T cell state-dependent proteomic differences but may have overlooked proteins subject to substoichiometric functional regulation. Cross-referencing these datasets with additional functional proteomics approaches, including phosphoproteomics profiling, could help uncover and validate such low-stoichiometry events^41^. Additionally, analysis of proteomes from the pan-CD3^+^ population may mask T cell subtype-specific changes or reactivity changes within distinct subcellular compartments, which will need to be addressed in future studies. Finally, expanding reactivity profiling platforms to other nucleophilic amino acid residues, including lysine, arginine, and tyrosine, will provide further insights into proteins involved in T cell dysfunction following chronic TCR stimulation, considering their distinct abundances, physicochemical properties, and functional roles.

## RESOURCE AVAILABILITY

### Lead contact

Further information and requests for resources and reagents should be directed to and will be fulfilled by the lead contact, Ekaterina V. Vinogradova (vinograd@rockefeller.edu).

### Materials availability

Questions and requests for chemical probes and plasmids generated in this study should be directed to and will be fulfilled by the lead contact with a completed materials transfer agreement.

### Data and code availability

- Raw proteomic data and RNA-sequencing data will be deposited to PRIDE and NCBI GEO.
- The code used for data processing and visualization will be deposited to Github.
- Any additional information required to reanalyze the data reported in this paper is available from the lead contact upon request.

## Supplementary Material

1
