# Supplementary material for: A post-translational regulatory map of chronic antigen-driven human T cell dysfunction
Source: bioRxiv. 2026 Mar 6:2026.03.04.709614. Preprint. [Version 1] doi: 10.64898/2026.03.04.709614 (PMC12991124; doi:10.64898/2026.03.04.709614)
Supplement: 1 [file NIHPP2026.03.04.709614V1-supplement-1.pdf]

bioRxiv preprint doi: https://doi.org/10.64898/2026.03.04.709614; this version posted March 6, 2026. The copyright holder for this preprint (which was not certified by peer review) is the author/funder, who has granted bioRxiv a license to display the preprint in perpetuity. It is made available under aCC-BY 4.0 International license.

(B) Supplementary Figures

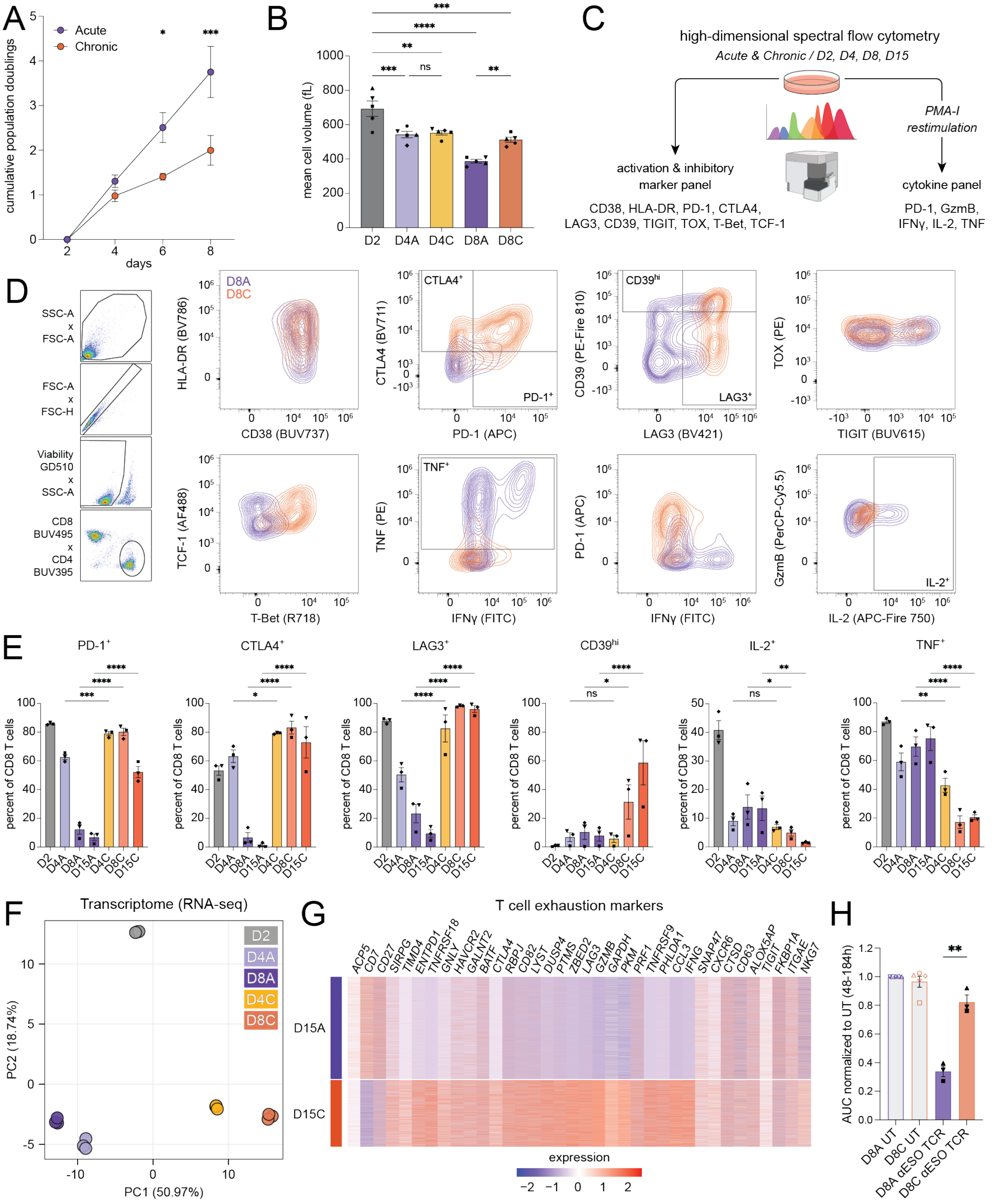

Figure S1. Validation of the *in vitro* T cell culturing system (Related to Figure 1).
(A) Population doublings of T cells cultured with or without chronic TCR stimulation from n = 3 independent donors compared by two-way ANOVA with Šídák's multiple comparisons test (* $p < 0.05$; *** $p < 0.001$).

bioRxiv preprint doi: https://doi.org/10.64898/2026.03.04.709614; this version posted March 6, 2026. The copyright holder for this preprint (which was not certified by peer review) is the author/funder, who has granted bioRxiv a license to display the preprint in perpetuity. It is made available under aCC-BY 4.0 International license.

(B) Cell volumes of activated, acutely, and chronically stimulated T cells compared by one-way repeated measures ANOVA with Šídák's multiple comparisons test (ns, not significant $p > 0.05$; ** $p < 0.01$; *** $p < 0.001$; **** $p < 0.0001$). Data are presented as mean ± SEM; n = 5.
(C) Schematic of flow cytometry experiments used to profile markers of activation and T cell dysfunction over time.
(D) Representative gating strategy and contour plots of activation/inhibitory marker and cytokine flow cytometry panels for $CD8^+$ T cells from D8 acute (purple) and chronic (orange) conditions. Contour plots represent >3,000 cells per condition.
(E) Quantification of T cell exhaustion markers and effector cytokines from flow cytometry time course experiments. Statistical comparison by one-way repeated measures ANOVA with Holm-Šídák's multiple comparisons test of D4A vs D4C, D8A vs D8C and D15A vs D15C conditions (ns, $p > 0.05$; * $p < 0.05$; ** $p < 0.01$; *** $p < 0.001$; **** $p < 0.0001$). Donor-matched conditions are indicated by differently shaped data points. Data are presented as mean ± SEM; n = 3.
(F) Principal component analysis (PCA) of bulk RNA-sequencing from n = 3 donors. The 5,000 protein-coding genes with the highest row-wise variance were used for PCA, and normalized count values were $\log_2$ transformed before PCA.
(G) Expression by scRNAseq of 36 of the 48 gene markers of exhausted $CD8^+$ T cells from Chu et al.[2] as measured in D15 $CD8^+$ T cells cultured with (D15C) or without (D15A) chronic TCR stimulation.
(H) Antigen-specific cancer cell killing of SK37 melanoma cells by D8A and D8C T cells transduced with NY-ESO-1-specific TCR. AUC of relative mCherry signal over 48-184 hours for n = 3-5 independent donors, normalized to untransduced (UT) D8A. Statistical comparison by two-tailed paired t test (** $p < 0.01$). Donor-matched conditions are indicated by differently shaped data points.

bioRxiv preprint doi: https://doi.org/10.64898/2026.03.04.709614; this version posted March 6, 2026. The copyright holder for this preprint (which was not certified by peer review) is the author/funder, who has granted bioRxiv a license to display the preprint in perpetuity. It is made available under aCC-BY 4.0 International license.

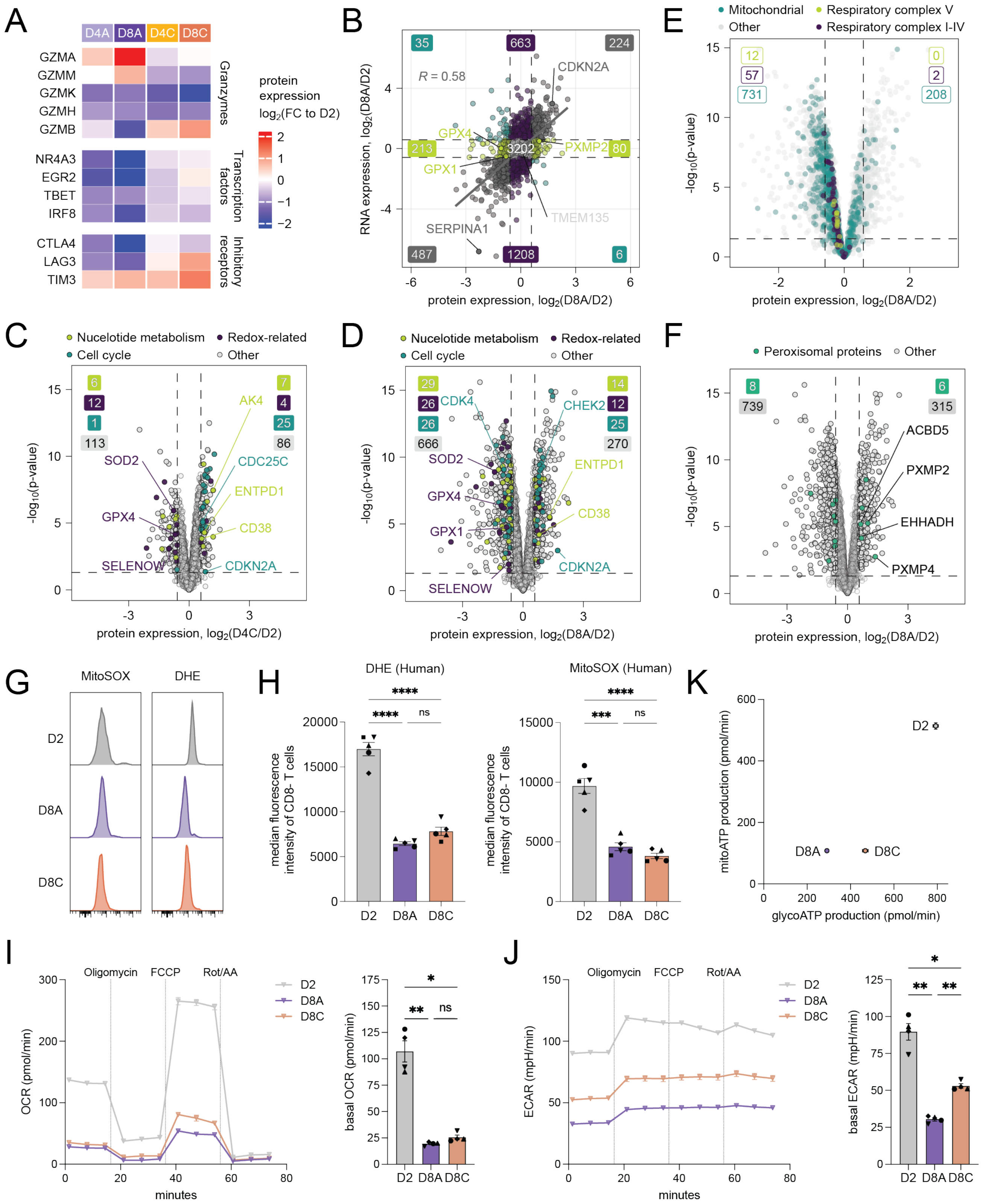

Figure S2. Transcriptomic and proteomic analyses facilitate identification of metabolic and redox pathways in chronically stimulated T cells (Related to Figure 2).
(A) Heatmap showing $\log_2$ fold protein expression changes in D4 and D8 T cells cultured with or without chronic stimulation relative to D2 T cells for selected granzymes, transcription factors, and inhibitory receptors. Data is averaged (mean) across n = 6 donors.

bioRxiv preprint doi: https://doi.org/10.64898/2026.03.04.709614; this version posted March 6, 2026. The copyright holder for this preprint (which was not certified by peer review) is the author/funder, who has granted bioRxiv a license to display the preprint in perpetuity. It is made available under aCC-BY 4.0 International license.

(B) Scatter plot showing $\log_2$ fold change of protein expression (x-axis) and RNA expression (y-axis) between D8A and D2 T cells. Genes quantified in both proteomic and transcriptomic datasets were used for the comparison. Pearson's correlation coefficient and regression line are shown. Transcriptomic data are from n = 3 donors and proteomic data are from n = 6 donors.
(C-D) Volcano plots showing $\log_2$ fold change of protein expression between D4C and D2 (C) and D8A and D2 (D) T cells. Genes related to nucleotide metabolism, redox regulation, and cell cycle are labeled based on Gene Ontology annotation (STAR Methods).
(E-F) Volcano plots showing $\log_2$ fold change of proteins between D8A and D2 T cells. Mitochondrial, respiratory complexes I-V (E), and peroxisomal proteins (F) are labeled based on Gene Ontology Cellular Component annotation.
(G) Representative histograms of oxidative stress markers in human $CD8^+$ T cells by flow cytometry using two ROS-reactive dyes: mitochondrial superoxide indicator (mitoSOX) and dihydroethidium (DHE; pan-cellular superoxides).
(H) Quantification of oxidative stress markers by flow cytometry for human $CD8^-$ T cells from five independent donors using two ROS-reactive dyes: mitoSOX and DHE. Statistical comparison by repeated measures one-way ANOVA with Šídák's multiple comparisons test (ns, $p > 0.05$; *** $p < 0.001$; **** $p < 0.0001$). Donor-matched conditions are indicated by differently shaped data points. Data are presented as mean ± SEM; n = 5.
(I-J) Representative plots of oxygen consumption rate (OCR) and extracellular acidification rate (ECAR) as measured by extracellular flux analysis in D2, D8A, and D8C cells. Injections of oligomycin (ATP synthase inhibitor), FCCP (mitochondrial uncoupler), and rotenone/antimycin A (electron transport chain inhibitors) are indicated by vertical lines. Quantification across n = 4 independent donors is shown on the right. Statistical comparison by one-way repeated measures ANOVA with Tukey's multiple comparisons test (ns $p > 0.05$; * $p < 0.05$; ** $p < 0.01$). Donor-matched conditions are indicated by differently shaped data points; data are presented as mean ± SEM.
(K) Representative energy map of calculated mitochondrial (mitoATP) and glycolytic (glycoATP) ATP production by D2, D8A, and D8C T cells.

bioRxiv preprint doi: https://doi.org/10.64898/2026.03.04.709614; this version posted March 6, 2026. The copyright holder for this preprint (which was not certified by peer review) is the author/funder, who has granted bioRxiv a license to display the preprint in perpetuity. It is made available under aCC-BY 4.0 International license.

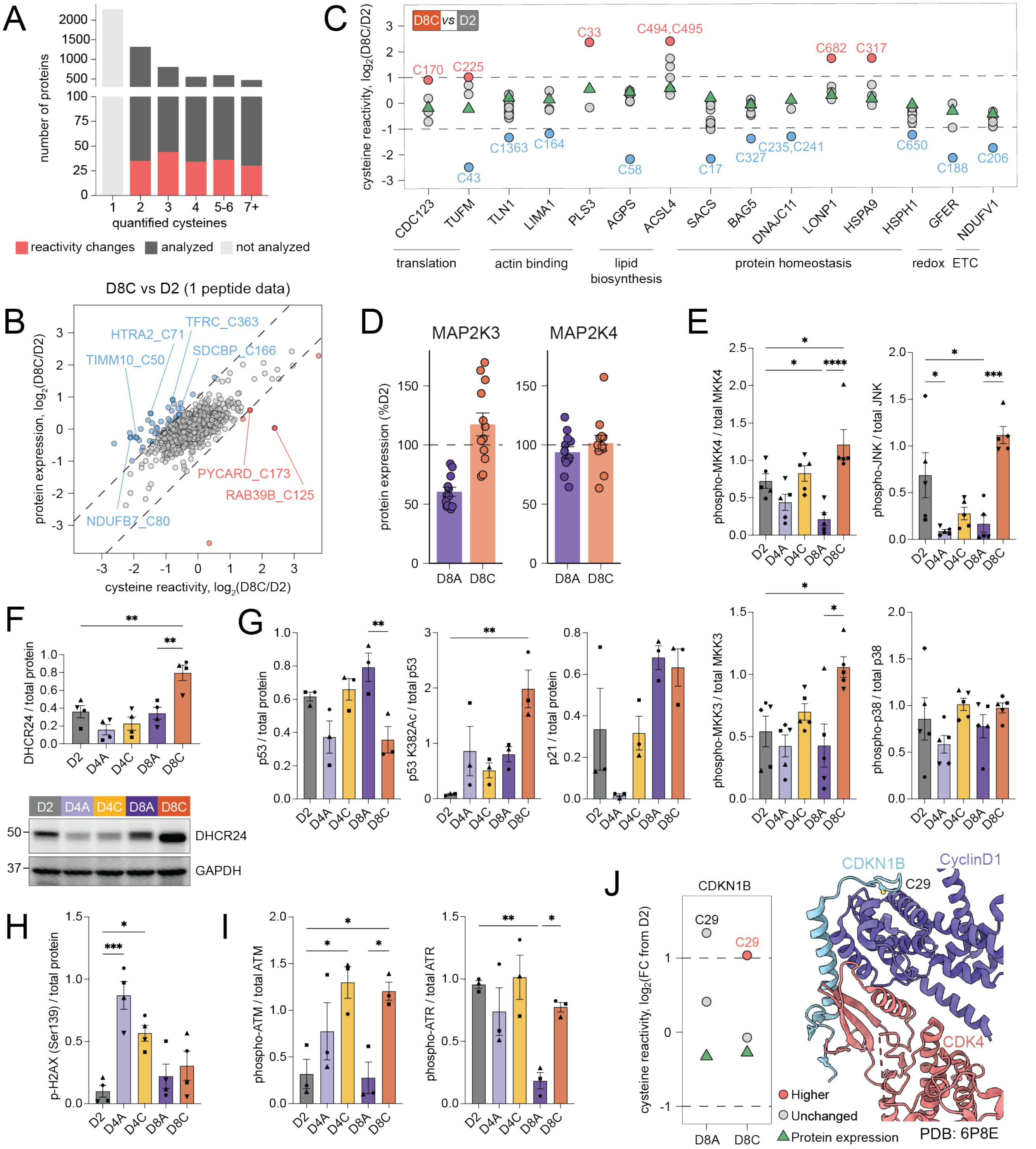

Figure S3. Reactivity profiling identifies changes in proteins spanning multiple key metabolic and stress signaling pathways (Related to Figure 3).

(A) Bar graph showing identified reactivity changes in proteins with specified numbers of quantified peptides across all conditions (D4A, D4C, D8A, and D8C) in comparison to D2. Proteins with one quantified cysteine were not considered for reactivity changes in this analysis (gray bar). See STAR Methods for more details.

(B) Cysteine reactivity (x-axis) and protein expression (y-axis) $\log_2$ fold changes between D8C and D2 T cells for peptides with one quantified cysteine in TMT-ABPP data. Dashed lines indicate an expression to reactivity fold change ratio cutoff of >2.

bioRxiv preprint doi: https://doi.org/10.64898/2026.03.04.709614; this version posted March 6, 2026. The copyright holder for this preprint (which was not certified by peer review) is the author/funder, who has granted bioRxiv a license to display the preprint in perpetuity. It is made available under aCC-BY 4.0 International license.

(C) Cysteine reactivity $\log_2$ fold change values between D8C and D2 T cells for select proteins. Protein expression $\log_2$ fold change values are shown as green triangles. Cysteine reactivity data is from n = 5 donors and protein expression data is from n = 6 donors.
(D) Bar graphs showing MAP2K3 and MAP2K4 protein expression levels in D8A and D8C T cells compared to D2 T cells (% of median D2 expression). Data are presented as mean ± SEM; n = 6 donors, 2 technical replicates per experimental condition.
(E) Western blot quantifications of T cell lysates at D2, D4, and D8 timepoints. Statistical comparisons by one-way repeated measures ANOVA with Holm-Šídák's multiple comparisons test of D4A vs D4C, D8A vs D8C, and D2 vs D8C (ns, $p > 0.05$; * $p < 0.05$; *** $p < 0.001$; **** $p < 0.0001$). Donor-matched conditions are indicated by differently shaped data points. Phosphorylation levels of MAP2K4 at Ser257 normalized by total MAP2K4; of JNK at Thr183 and Tyr185 normalized by total JNK; of MAP2K3 at Ser189 and Ser207 normalized to total MAP2K3, and of p38 at Thr180 and Tyr182 normalized to total p38 Data are presented as mean ± SEM; n = 4-5.
(F) Western blot analysis and quantification of DHCR24 protein expression in activated, acutely, and chronically stimulated T cells. Statistical comparison by one-way repeated measures ANOVA with Holm-Šídák's multiple comparisons test of D2 versus all conditions, D4A vs D4C, and D8A vs D8C (ns, $p > 0.05$; ** $p < 0.01$). Donor-matched conditions are indicated by differently shaped data points. Data are presented as mean ± SEM; n = 4.
(G) Western blot quantifications of T cell lysates at D2, D4, and D8 timepoints. Statistical comparisons by one-way repeated measures ANOVA with Holm-Šídák's multiple comparisons test of D4A vs D4C, D8A vs D8C, and D2 vs D8C (ns, $p > 0.05$; * $p < 0.05$; ** $p < 0.01$). Donor-matched conditions are indicated by differently shaped data points. p53 (DO-1) level was normalized to total protein, p53 acetylation at lysine 382 was normalized to total p53 level, and p21 was normalized to total protein (n = 3).
(H) Western blot analysis and quantification of pH2AX (Ser139) protein normalized to total protein in activated, acutely, and chronically stimulated T cells. Statistical comparison by one-way repeated measures ANOVA with Holm-Šídák's multiple comparisons test of D2 versus all conditions, D4A vs D4C, and D8A vs D8C (ns, $p > 0.05$; * $p < 0.05$; *** $p < 0.001$). Donor-matched conditions are indicated by differently shaped data points. Data are presented as mean ± SEM; n = 4.
(I) Phosphorylation levels of ATM at Ser1981 normalized by total ATM and of ATR at Thr1989 normalized by total ATR. Statistical comparison by one-way repeated measures ANOVA with Holm-Šídák's multiple comparisons test of D2 versus all conditions, D4A vs D4C, and D8A vs D8C (* $p < 0.05$; ** $p < 0.01$; non-significant comparisons not shown).
(J) Left: Cysteine reactivity $\log_2$ fold change from D2 T cells for cysteines in CDKN1B. Log_2 fold change of protein expression from whole proteome experiments is shown as green triangles. Right: Crystal structure of Cyclin D1 (purple), CDK4 (orange), and CDKN1B (blue) protein complex (PDB: 6P8E[7]) with differentially reactive cysteine CDKN1B_C29 labeled at the protein-protein interaction surface with Cyclin D1.

bioRxiv preprint doi: https://doi.org/10.64898/2026.03.04.709614; this version posted March 6, 2026. The copyright holder for this preprint (which was not certified by peer review) is the author/funder, who has granted bioRxiv a license to display the preprint in perpetuity. It is made available under aCC-BY 4.0 International license.

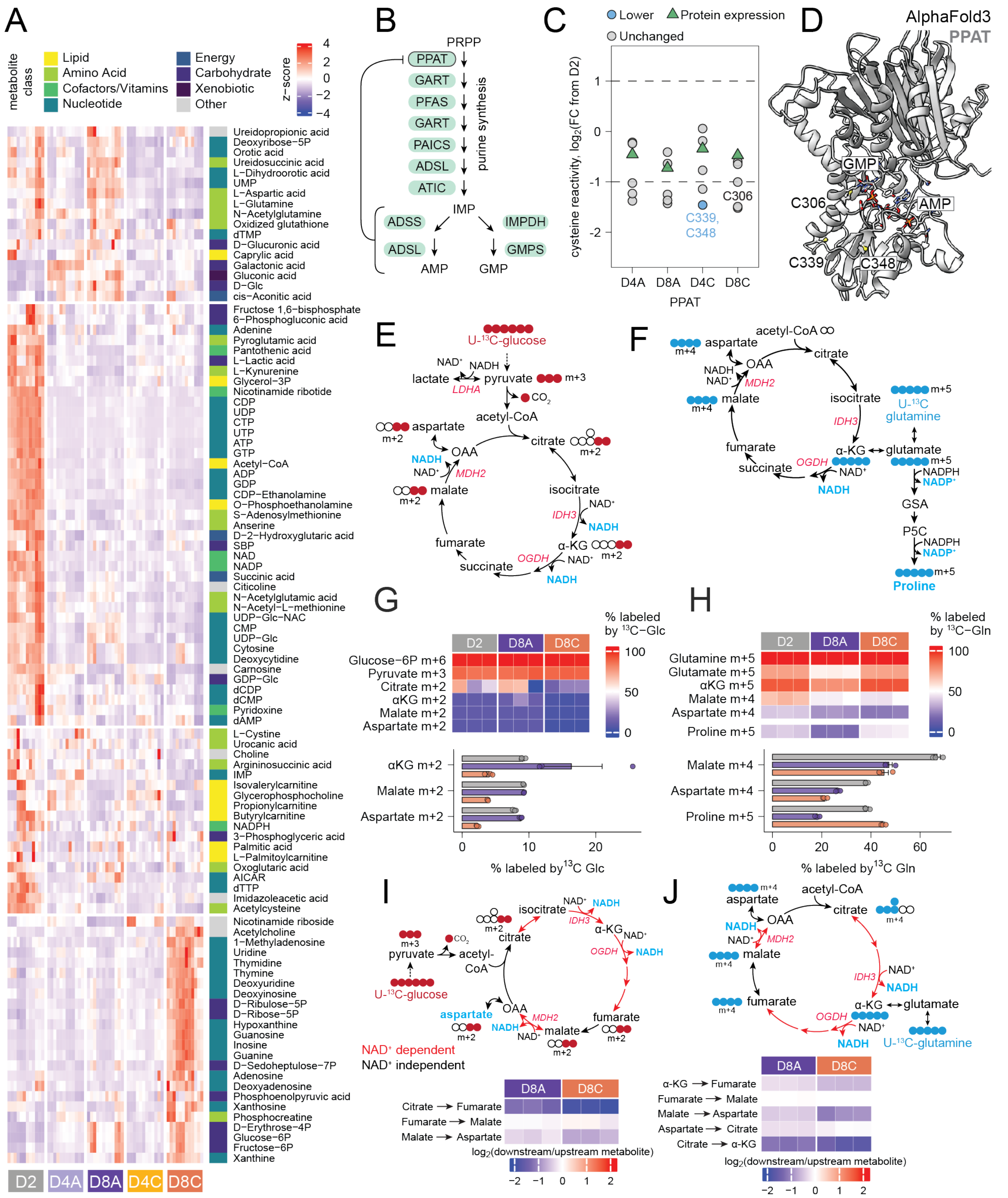

Figure S4. Metabolic profiling highlights reduced mitochondrial NAD$^+$ regeneration, increased non-oxidative TCA cycle metabolism and activation of nucleotide salvage in chronically stimulated T cells (Related to Figure 4).

(A) Heatmap showing z-score calculated from metabolite signal intensity. Top 100 metabolites with the highest variance across samples are shown. Annotations at the top indicate metabolite class. Data are from n = 4 donors.

bioRxiv preprint doi: https://doi.org/10.64898/2026.03.04.709614; this version posted March 6, 2026. The copyright holder for this preprint (which was not certified by peer review) is the author/funder, who has granted bioRxiv a license to display the preprint in perpetuity. It is made available under aCC-BY 4.0 International license.

(B) Schematic showing the role of PPAT in *de novo* purine biosynthesis.
(C) Cysteine reactivity $\log_2$ fold change from D2 cells for all quantified cysteines in PPAT across different conditions. Log_2 fold change of protein expression from whole proteome experiments is shown as green triangles.
(D) Predicted AlphaFold3 PPAT structure in complex with AMP and GMP.
(E) Schematic depicting how oxidative metabolism of [U-^{13}C] glucose generates labeled metabolites associated with the TCA cycle. Red circles represent ^{13}C-labeled carbons.
(F) Schematic depicting how oxidative and non-oxidative metabolism of [U-^{13}C] glutamine generates labeled metabolites associated with the TCA cycle. Blue circles represent ^{13}C-labeled carbons.
(G) Heatmap (top) and bar graph (bottom) showing fractional labeling by [U-^{13}C] glucose of indicated metabolites in D2, D8A, and D8C T cells. Representative data from one of two independent donors (n = 3 replicates/donor) showing similar results. Data are presented as mean ± SEM.
(H) Heatmap (top) and bar graph (bottom) showing fractional labeling by [U-^{13}C] glutamine of indicated metabolites in D2, D8A, and D8C T cells. Representative data from one of two independent donors (n = 3 replicates/donor) showing similar results. Data are presented as mean ± SEM.
(I-J) Schematic showing NAD$^+$-dependent and NAD$^+$-independent steps of glucose (I) and glutamine (J) metabolism. Colored circles indicate ^{13}C-labeled carbons. Heatmaps of the $\log_2$ fold change in percentage of labeling between downstream and upstream metabolites in D8A and D8C T cells.

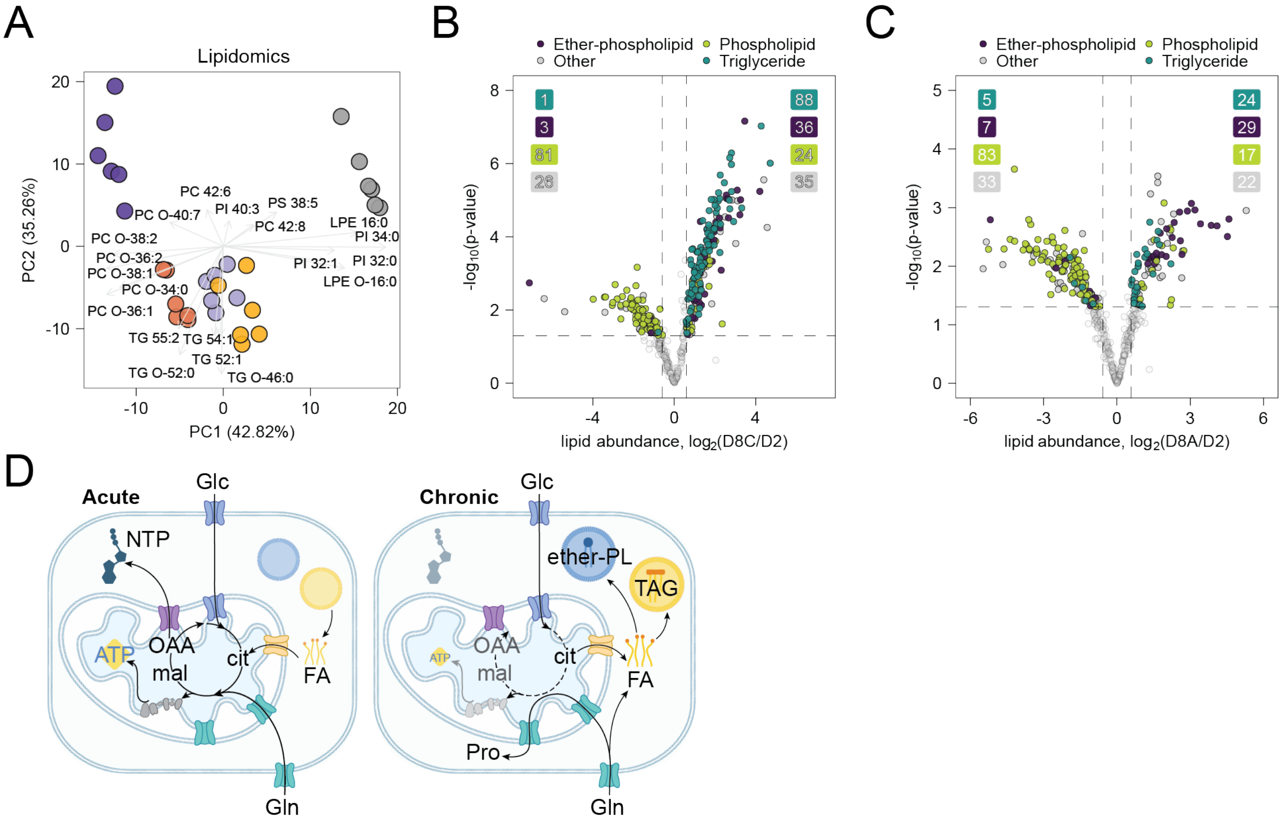

Figure S5. Lipidomic characterization of chronically stimulated T cells (Related to Figure 4).
(A) PCA of whole-cell lipidomics data from n = 2 donors (3 technical replicates each). Signal intensity values were $\log_2$ transformed and only metabolites quantified in all replicates were used. The top and bottom five loadings from PC1 and PC2 are shown with grey arrows.
(B-C) Volcano plot showing $\log_2$ fold changes in lipid abundances between D8C and D2 (B) and D8A and D2 (C) T cells. Dashed lines represent cutoffs of p-value < 0.05 and fold-change > 1.5. Data are from n = 2 donors.
(D) Schematic showing metabolic differences between acutely and chronically stimulated T cells.

bioRxiv preprint doi: https://doi.org/10.64898/2026.03.04.709614; this version posted March 6, 2026. The copyright holder for this preprint (which was not certified by peer review) is the author/funder, who has granted bioRxiv a license to display the preprint in perpetuity. It is made available under aCC-BY 4.0 International license.

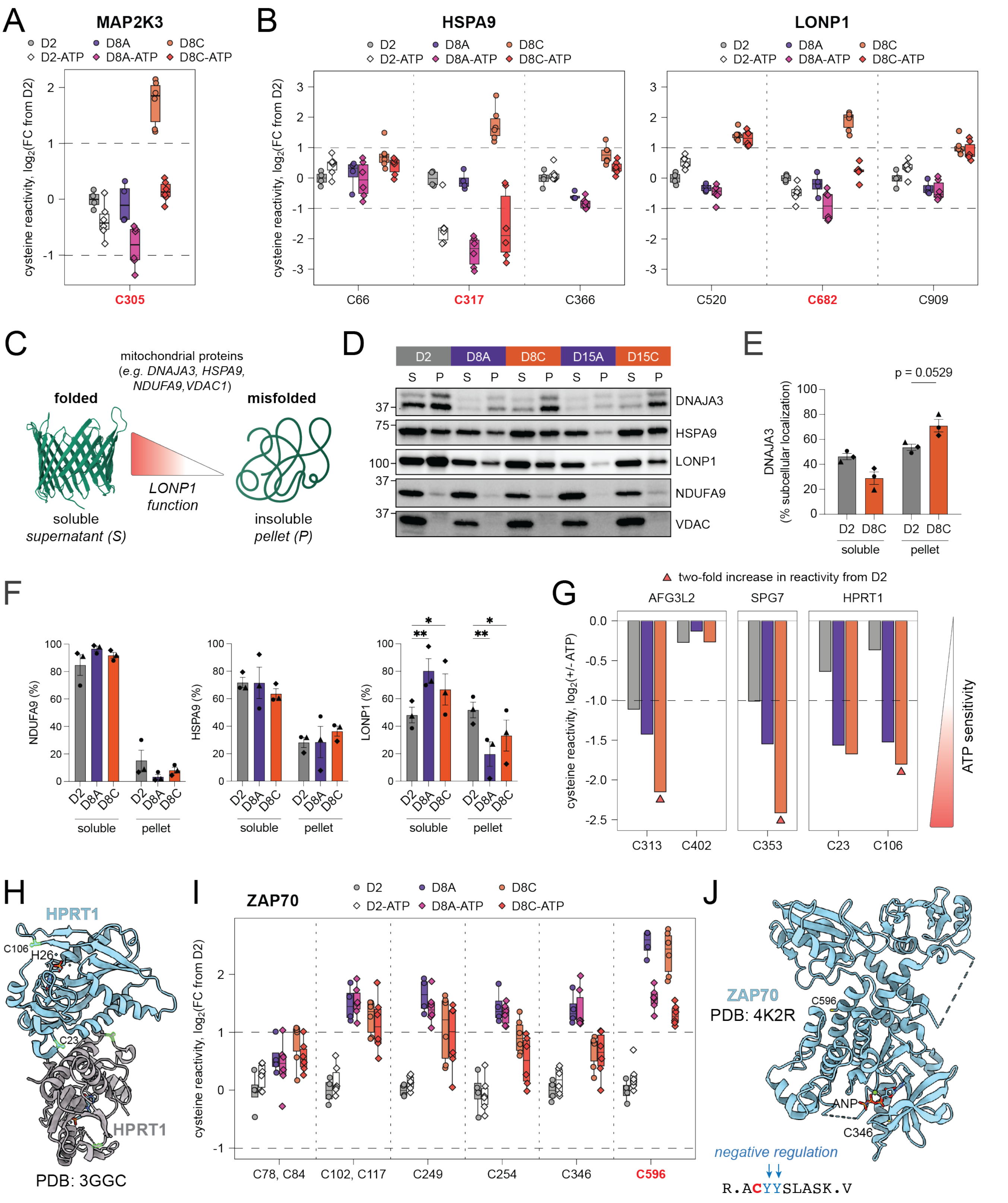

Figure S6. Identification of ATP-sensitive cysteine reactivity changes during chronic TCR stimulation (Related to Figure 5).

(A-B) Boxplot of $\log_2$ fold changes in cysteine reactivity of MAP2K3, HSPA9, and LONP1 in control (circle) and ATP add-back (diamond) conditions in D2, D8A, and D8C T cells compared to D2 control condition. Boxes mark the lower and upper quartiles, horizontal black lines mark the median value, and whiskers extend to the furthest point within 1.5x the interquartile range.

bioRxiv preprint doi: https://doi.org/10.64898/2026.03.04.709614; this version posted March 6, 2026. The copyright holder for this preprint (which was not certified by peer review) is the author/funder, who has granted bioRxiv a license to display the preprint in perpetuity. It is made available under aCC-BY 4.0 International license.

(C) Schematic of detergent-soluble and insoluble fractions for identification of misfolded or aggregated proteins in activated, acutely, and chronically stimulated T cells[8].
(D) Comparison of solubility of mitochondrial proteins in D2, D8A, D8C, D15A, and D15C T cells. Cell lysate was separated into 1% Triton X-100-soluble fraction (S) and insoluble pellet (P), and protein levels were analyzed by Western blot.
(E) Quantification of the distribution of DNAJA3 between soluble and insoluble fractions in activated and chronically stimulated T cells. Statistical comparison by two-tailed paired t test (ns, $p > 0.05$). Donor-matched conditions are indicated by differently shaped data points. Data are from n = 3 donors.
(F) Quantification of the distribution of NDUFA9, HSPA9, and LONP1 between soluble and insoluble fractions in activated, acutely, and chronically stimulated T cells. Statistical comparison by one-way repeated measures ANOVA with Šídák's multiple comparisons test (* $p < 0.05$; ** $p < 0.01$; non-significant comparisons not displayed). Donor-matched conditions are indicated by differently shaped data points. Data are presented as mean ± SEM; n = 3.
(G) Bar graph of $\log_2$ fold changes in cysteine reactivity of AFG3L2, SPG7, and HPRT1 proteins in D2, D8A, and D8C cells between control and ATP add-back conditions. Red triangles below bars denote a cysteine showing a general reactivity change (FC > 2) compared to D2 cells. Data are from n = 2 donors analyzed across two independent cysteine reactivity profiling experiments.
(H) Crystal structure of HPRT1 in complex with 9-(2-phosphonoethoxyethyl)hypoxanthine (PDB: 3GGC[9]). Cysteines 23 and 106 are labeled.
(I) Boxplot of $\log_2$ fold changes in cysteine reactivity of ZAP70 in control (circle) and ATP add-back (diamond) conditions in D2, D8A, and D8C T cells compared to D2 control condition. Data are from n = 2 donors analyzed across two independent cysteine reactivity profiling experiments. Boxes mark the lower and upper quartiles, horizontal black lines mark the median value, and whiskers extend to the furthest point within 1.5x the interquartile range.
(J) Crystal structure of ZAP70 in complex with the non-hydrolyzable ATP analog ANP (PDB: 4K2R[10]). Cysteines 346 and 596 are labeled.

bioRxiv preprint doi: https://doi.org/10.64898/2026.03.04.709614; this version posted March 6, 2026. The copyright holder for this preprint (which was not certified by peer review) is the author/funder, who has granted bioRxiv a license to display the preprint in perpetuity. It is made available under aCC-BY 4.0 International license.

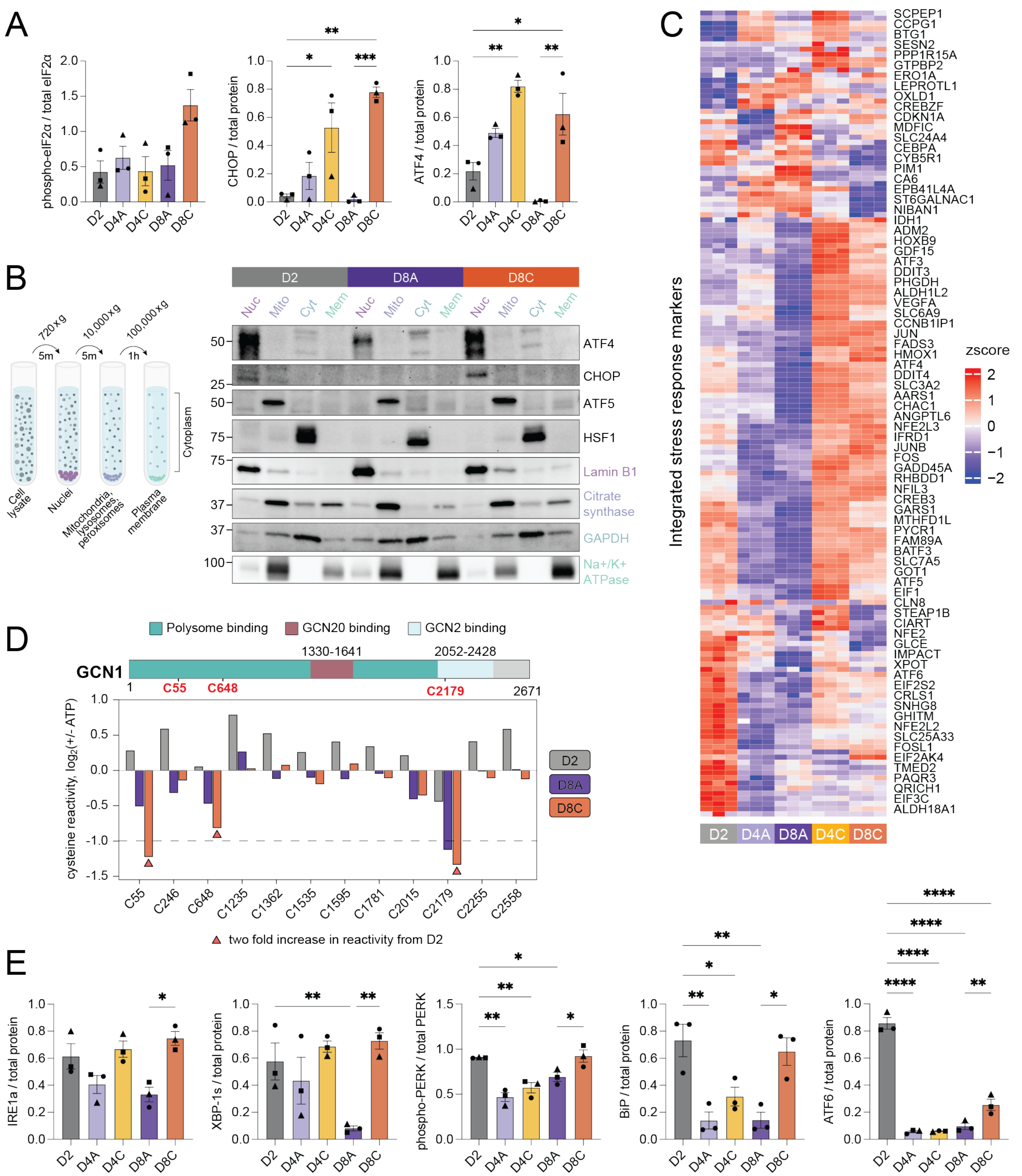

Figure S7. Differential activation of integrated, mitochondrial, and ER stress responses during chronic TCR stimulation (Related to Figure 6).

(A) Western blot quantifications of ISR proteins in T cell lysates at D2, D4 and D8 timepoints. Statistical comparisons by one-way repeated measures ANOVA with Holm-Šídák's multiple comparisons test of D4A vs D4C, D8A vs D8C, and D2 vs D8C (* $p < 0.05$; ** $p < 0.01$, *** $p < 0.001$). Donor-matched conditions are indicated by differently shaped data points. ATF4 and CHOP were normalized to total protein level (n = 3). Phosphorylation levels of eIF2a at Ser51 were normalized by total eIF2a.

bioRxiv preprint doi: https://doi.org/10.64898/2026.03.04.709614; this version posted March 6, 2026. The copyright holder for this preprint (which was not certified by peer review) is the author/funder, who has granted bioRxiv a license to display the preprint in perpetuity. It is made available under aCC-BY 4.0 International license.

(B) Schematic overview of subcellular fractionation of primary human T cells using differential centrifugation (left) and Western blot analysis of subcellular localization of transcription factors involved in ISR or mtUPR (right).
(C) Heatmap of bulk RNA-Seq expression of ISR genes[11]. Every second gene is labeled.
(D) Bar graph of $\log_2$ fold changes in cysteine reactivity of GCN1 in D2, D8A, and D8C cells between control and ATP add-back conditions. Red triangles below bars denote a cysteine showing a general reactivity change (FC > 2) compared to D2 cells. Data are from n = 2 independent cysteine reactivity profiling experiments. Sequence of GCN1 with annotated polysome-, GCN20-, and GCN2-binding domains[12,13].
(E) Western blot quantifications of ER-stress proteins in T cell lysates at D2, D4, and D8 timepoints. Statistical comparisons by one-way repeated measures ANOVA with Holm-Šídák's multiple comparisons test of D2 versus each condition, D4A vs D4C, and D8A vs D8C (* $p < 0.05$; ** $p < 0.01$, **** $p < 0.0001$; non-significant comparisons not displayed). Donor-matched conditions are indicated by differently shaped data points. Data are presented as mean ± SEM; n = 3.

bioRxiv preprint doi: https://doi.org/10.64898/2026.03.04.709614; this version posted March 6, 2026. The copyright holder for this preprint (which was not certified by peer review) is the author/funder, who has granted bioRxiv a license to display the preprint in perpetuity. It is made available under aCC-BY 4.0 International license.

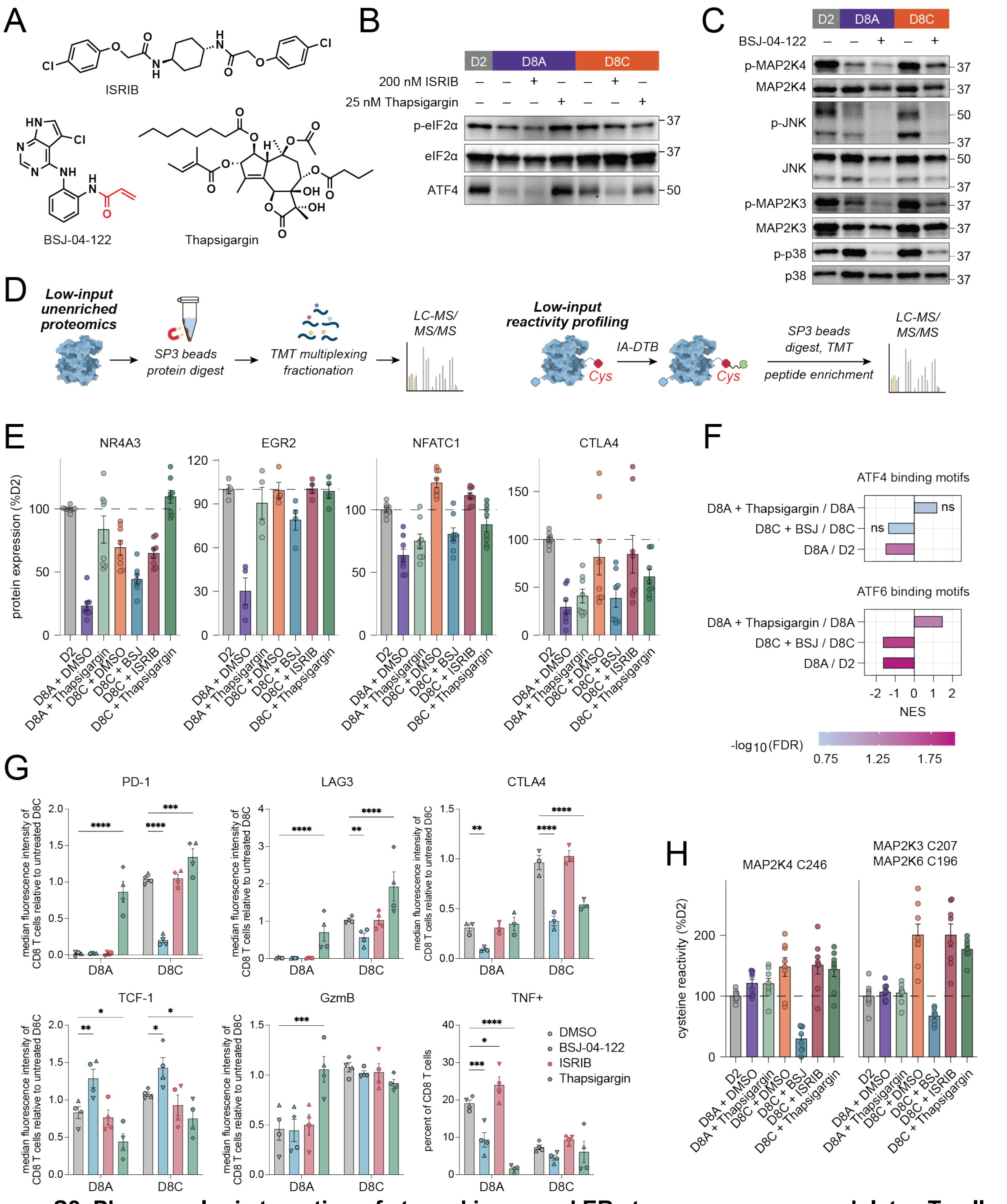

Figure S8. Pharmacologic targeting of stress kinase and ER stress responses modulates T cell dysfunction (Related to Figure 6).

(A) Chemical structures of ISRIB, BSJ-04-122, and Thapsigargin. The covalent electrophilic group of BSJ-04-122 is highlighted in red.

(B) Western blot analysis of phospho-eIF2α and ATF4 in D2, D8A, and D8C T cells with or without the indicated inhibitor treatments.

bioRxiv preprint doi: https://doi.org/10.64898/2026.03.04.709614; this version posted March 6, 2026. The copyright holder for this preprint (which was not certified by peer review) is the author/funder, who has granted bioRxiv a license to display the preprint in perpetuity. It is made available under aCC-BY 4.0 International license.

(C) Western blot analysis of phosphorylation levels of MAP2K4 (Ser257), JNK (Thr183/Tyr185), MAP2K3 (Ser189), and p38 (Thr180/Tyr182) relative to protein expression in D2, D8A, and D8C T cells with or without 10 μM BSJ-04-122 treatment.
(D) Schematic of low-input unenriched proteomics and cysteine reactivity profiling. See STAR Methods for more details.
(E) Bar graphs showing the protein expression levels of T cell exhaustion markers, including NR4A3, EGR2, NFATC1, and CTLA4 in D8A and D8C T cells with or without inhibitor treatments compared to D2 T cells (% of median D2 expression). Data are presented as mean ± SEM; n = 4 donors (2 technical replicates per experimental condition).
(F) GSEA of unenriched proteomics data from T cells on the hallmark gene sets ATF4 and ATF6 binding motifs. Comparisons not passing an FDR < 0.05 are labelled n.s.. Unenriched proteomics data are from n = 4 donors.
(G) Quantification by flow cytometry of $CD8^+$ T cell expression of key markers of T cell exhaustion. Data for PD-1, LAG3, CTLA4, TCF-1, GzmB, and TNF presented as median fluorescent intensity (MFI) relative to the MFI of untreated D8C cells from the matched donor. Comparison by two-way repeated measures ANOVA with Dunnett's multiple comparisons test (* p < 0.05; ** p < 0.01; *** p < 0.001; **** p < 0.0001). Each condition compared to DMSO-treated control; non-significant comparisons not displayed. Data are presented as mean ± SEM; n = 4 donors. Donor-matched conditions are indicated by differently shaped data points.
(H) Cysteine reactivity profiling of MAP2K4 and MAP2K3/6 in D8A and D8C T cells with or without inhibitor treatments compared to D2 T cells (% of median D2 expression). Data are presented as mean ± SEM; n = 4 donors (2 technical replicates per experimental condition).

bioRxiv preprint doi: https://doi.org/10.64898/2026.03.04.709614; this version posted March 6, 2026. The copyright holder for this preprint (which was not certified by peer review) is the author/funder, who has granted bioRxiv a license to display the preprint in perpetuity. It is made available under aCC-BY 4.0 International license.

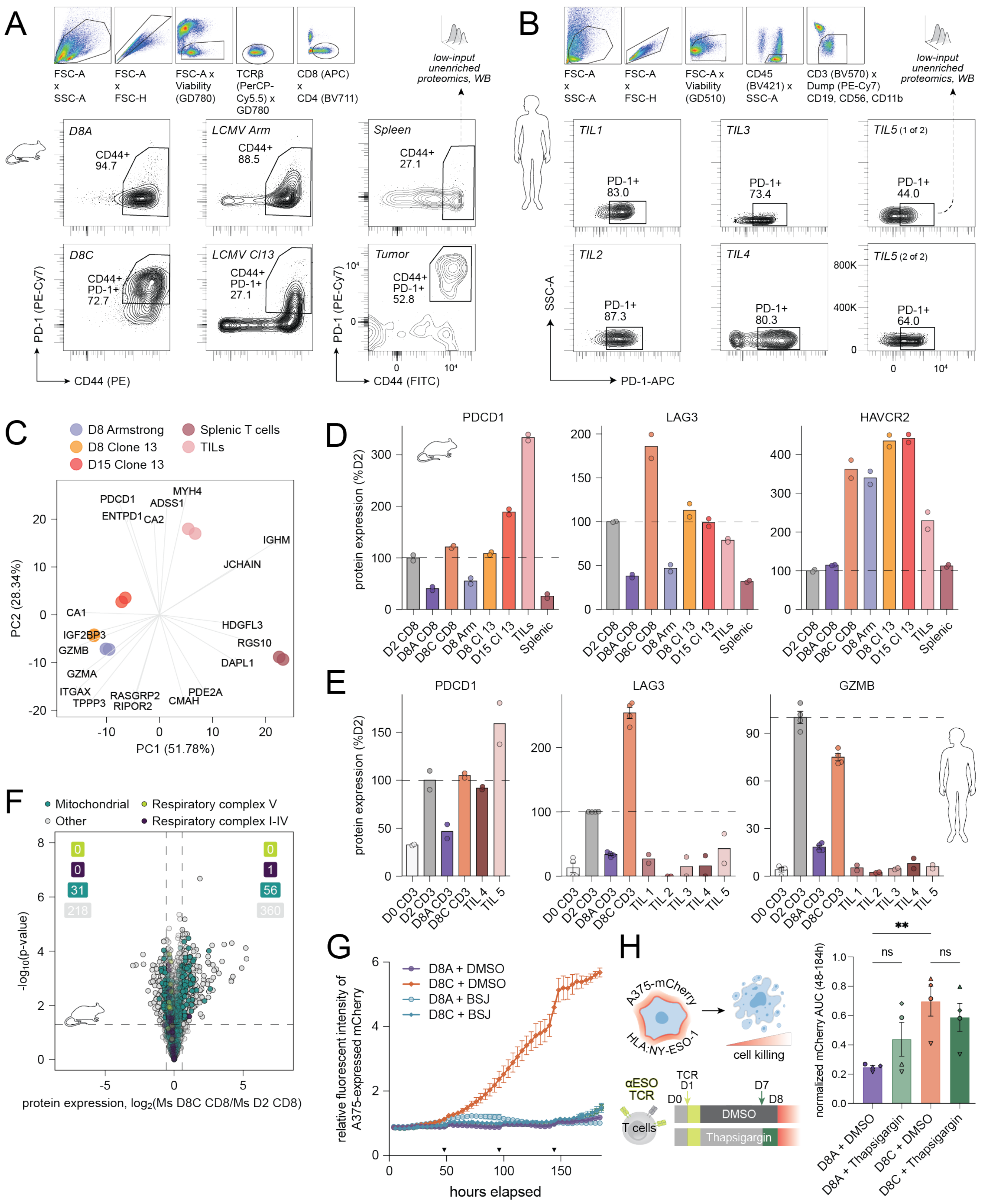

Figure S9. Phenotypic validation of T cell dysfunction in exhausted T cells from mouse and human samples (Related to Figure 7).

(A) Gating strategy for mouse T cells sorted as shown in **Figure 7A**. Contour plots below show representative CD44 and PD-1 expression for individual conditions.

(B) Gating strategy for human T cells sorted as shown in **Figure 7B**. Contour plots below show representative PD-1 expression for individual conditions.

bioRxiv preprint doi: https://doi.org/10.64898/2026.03.04.709614; this version posted March 6, 2026. The copyright holder for this preprint (which was not certified by peer review) is the author/funder, who has granted bioRxiv a license to display the preprint in perpetuity. It is made available under aCC-BY 4.0 International license.

(C) PCA of unenriched proteomic data of T cells isolated from mice bearing either LCMV infections or KPC tumors. Protein ratio values were $\log_2$ transformed and only proteins quantified in all experiments (4,343) were used. The five highest and five lowest loadings from PC1 and PC2 were plotted.
(D) Bar graphs showing the protein expression levels of T cell exhaustion markers, including PDCD1, LAG3, and HAVCR2 in *in vitro* and *in vivo* mouse T cells compared to *in vitro*-generated D2 T cells (% of mean D2 expression). Data are presented as mean; n = 1 donor (2 technical replicates per experimental condition).
(E) Bar graphs showing the protein expression levels of T cell exhaustion markers, including PDCD1, LAG3, and GZMB in TILs from RCC patients compared to *in vitro*-generated D2 T cells (% of mean D2 expression). Data are presented as mean ± SEM; n = 5 donors (2 technical replicates per experimental condition).
(F) Volcano plot showing $\log_2$ fold changes of protein expression between D8C and D2 mouse $CD8^+$ T cells. Mitochondrial and respiratory chain subunits I-V are labeled based on Gene Ontology Cellular Component annotation (STAR Methods). Dashed lines represent cutoffs of p-value < 0.05 and fold-change > 1.5. Data are from n = 1 donor.
(G) A representative cell viability curve of NY-ESO-1-expressing A375 melanoma cells engineered to express mCherry following 184 hours of co-culture with T cells pre-treated with DMSO or BSJ-04-122, normalized to 0 h condition. Inverted triangles on x axis indicate timepoints when A375 melanoma cells where added to the co-culture.
(H) Schematic (left) and quantification (right) of antigen-specific cancer cell killing by D8A and D8C T cells transduced with NY-ESO-1-specific TCR and treated with DMSO or Thapsigargin. Normalized AUC of mCherry intensity for 48-184 hours normalized to untreated D8A condition. Statistical comparison by one-way repeated measures ANOVA with Šídák's multiple comparisons test (ns p > 0.05; ** p < 0.01). Donor-matched conditions are indicated by differently shaped data points. A representative cell viability curve is shown on the right.

bioRxiv preprint doi: https://doi.org/10.64898/2026.03.04.709614; this version posted March 6, 2026. The copyright holder for this preprint (which was not certified by peer review) is the author/funder, who has granted bioRxiv a license to display the preprint in perpetuity. It is made available under aCC-BY 4.0 International license.

(C) Supplementary Data Set Legends

Data S1

#	Title	Description
1	S1-1 Bulk RNA Sequencing Data	Differential gene expression analysis of bulk RNA-Seq data from activated, acutely, and chronically stimulated T cells (related to Figures 1 and S1).

Table S1-1: Differential gene expression analysis from DESeq2. RNA counts were normalized using DESeq2's median of ratios method. Genes with zero reads mapping are not displayed. Data are from n = 3 donors.

Data S2

#	Title	Description
1	S2-1 Unenriched proteomics	Whole proteome (TMT-exp) data from activated, acutely, and chronically stimulated human T cells (related to Figures 2 and S2)
2	S2-2 PCA pathway enrichment	Gene ontology biological process enrichment of principal components (related to Figure 2)
3	S2-3 Bulk RNA vs WP	Whole proteome (TMT-exp) and transcriptome (bulk RNA-Seq) data (related to Figures 2 and S2)
4	S2-4 Reactivity changes	Cysteine reactivity changes identified from IA-DTB reactivity TMT-ABPP data (related to Figures 3, 4, and S3)
5	S2-5 Cysteine reactivity vs WP	Cysteine reactivity values (TMT-ABPP) versus whole proteome (TMT-exp) values for D4A, D8A, D4C, and D8C human T cells (related to Figure S3)
6	S2-6 ATP add-back	Cysteine reactivity values (TMT-ABPP) for activated, acutely, and chronically stimulated human T cells with and without addition of ATP (related to Figures 5, S6, and S7)
7	S2-7 Low-input WP, inhibitors	Low-input whole proteome (TMT-exp) data from acutely and chronically stimulated human T cells with and without inhibitor treatment (related to Figures 6 and S8)
8	S2-8 Low-input RC, inhibitors	Low-input cysteine reactivity (TMT-ABPP) for activated, acutely, and chronically stimulated human T cells with and without inhibitor treatment (related to Figures 6 and S8)
9	S2-9 Low-input WP, mouse	Low-input whole proteome (TMT-exp) data from *in vitro*-generated mouse D2, D8A, D8C T cells, and T cells isolated from mice bearing either LCMV infections or KPC tumors (related to Figures 7 and S9)
10	S2-10 Low-input WP, TILs	Low-input whole proteome (TMT-exp) data from *in vitro*-generated human D0, D2, D8A, D8C T cells, and TILs isolated from five independent RCC patients (related to Figures 7 and S9)

bioRxiv preprint doi: https://doi.org/10.64898/2026.03.04.709614; this version posted March 6, 2026. The copyright holder for this preprint (which was not certified by peer review) is the author/funder, who has granted bioRxiv a license to display the preprint in perpetuity. It is made available under aCC-BY 4.0 International license.

Table S2-1: TMT-exp data showing protein expression changes in human T cells cultured for 4 or 8 days with or without chronic stimulation compared to activated D2 T cells. Data are from n = 6 donors.
Table S2-2: Gene ontology biological process pathways that were enriched (Benjamini Hochberg corrected p-values ≤ 0.01) in gene set enrichment analysis.
Table S2-3: TMT-exp and bulk RNA-seq data showing gene and protein expression changes in human T cells cultured for 4 or 8 days with or without chronic stimulation compared to activated D2 human T cells.
Table S2-4: Proteins with identified cysteine reactivity changes in at least one condition (D4A, D8A, D4C, D8C) compared to D2 human T cells. Data are median from n = 5 donors.
Table S2-5: Reactivity fold change values versus protein expression fold change values for human T cells cultured for 4 or 8 days with or without chronic stimulation compared to activated D2 human T cells.
Table S2-6: Cysteine reactivity values for human T cells in D2, D8A, and D8C stimulation conditions with and without the addition of ATP. Data are from n = 2 donors.
Table S2-7: Low-input TMT-exp data showing protein expression changes in human T cells cultured for 8 days under chronic stimulation with or without inhibitor treatment compared to activated D2 cells. Data are from n = 4 donors.
Table S2-8: Low-input TMT-ABPP data for human T cells cultured for 8 days under chronic stimulation with or without inhibitor treatment compared to activated D2 cells. Data are from n = 4 donors.
Table S2-9: Low-input TMT-exp data showing protein expression changes in $CD8^+$ T cells from mice infected with acute or chronic strains of lymphocytic choriomeningitis virus (LCMV) infection as well as mice bearing subcutaneous KPC tumors in comparison with mouse T cells undergoing acute or chronic stimulation *in vitro*. Data is shown normalized to *in vitro*-generated D2 cells.
Table S2-10: Low-input TMT-exp data showing protein expression changes in *in vitro*-generated acutely or chronically stimulated human $CD3^+$ T cells and PD-1^+ cells isolated from five independent renal cell carcinoma (RCC) patients compared to *in vitro*-generated D2 cells. Data are from n = 5 patients.

Data S3

#	Title	Description
1	S3-1 Polar metabolites	Abundance of polar metabolites in activated, acutely, and chronically stimulated human T cells (related to Figures 4 and S4)
2	S3-2 Whole cell lipidomics	Abundance of lipids in activated, acutely, and chronically stimulated human T cells (related to Figures 4 and S5)
3	S3-3 Isotope tracing	Abundance of stable metabolite isotopologues in D2, D8A, or D8C human T cells cultured in the presence of universally ^{13}C-labeled glucose or glutamine (related to Figure S4)
4	S3-4 Additional isotope tracing	Abundance of stable metabolite isotopologues in D8A or D8C human T cells cultured in the presence of universally ^{13}C-labeled glucose or glutamine, used for processivity analysis (related to Figures S4)

Table S3-1: Polar metabolite abundance values in human T cells at day 2, day 4, or day 8 of culturing with or without chronic stimulation as determined by LC-MS/MS analysis. The channel ratio values after quantile regression imputation of left censored data (QRILC) and cell volume normalization are shown alongside raw signal intensity values and differential expression calculations. Data are from n = 4 donors.
Table S3-2: Lipid abundance values in human T cells at day 2, day 4, or day 8 of culturing with or without chronic stimulation. The channel ratio values after quantile regression imputation of left censored data

bioRxiv preprint doi: https://doi.org/10.64898/2026.03.04.709614; this version posted March 6, 2026. The copyright holder for this preprint (which was not certified by peer review) is the author/funder, who has granted bioRxiv a license to display the preprint in perpetuity. It is made available under aCC-BY 4.0 International license.

(QRILC) and cell volume normalization are shown alongside raw signal intensity values and differential expression calculations. Data are from n = 2 donors.

Table S3-3: Abundance of stable metabolite isotopologues in D2, D8A, or D8C human T cells cultured in the presence of universally ^{13}C-labeled glucose or glutamine.

Table S3-4: Abundance of stable metabolite isotopologues in D8A, or D8C human T cells cultured in the presence of universally ^{13}C-labeled glucose or glutamine, used for processivity analysis.

bioRxiv preprint doi: https://doi.org/10.64898/2026.03.04.709614; this version posted March 6, 2026. The copyright holder for this preprint (which was not certified by peer review) is the author/funder, who has granted bioRxiv a license to display the preprint in perpetuity. It is made available under aCC-BY 4.0 International license.

(D) Biological methods

EXPERIMENTAL MODEL AND SUBJECT DETAILS

Human samples

All studies involving primary human T cells used in *in vitro* experiments were conducted using buffy coats obtained from the New York Blood Center, and leukopaks or LRS cones from STEMCELL Technologies. The primary human T cells were isolated from blood samples collected from randomly selected, de-identified healthy donors. Donor sex was not recorded. Human tumor tissue samples were processed as previously described[14]. Briefly, samples were obtained from patients with pathologically confirmed kidney cancer who had consented to MSKCC's institutional biobanking protocol (MSKCC IRB #06-107). Utilization of biospecimens was approved under IRB #12-237 and IRB #20-025. Samples were obtained from patients undergoing clinically indicated operative resections of their primary kidney tumor. Samples were directly obtained from the operating room during nephrectomy. Tumor tissue was transported from the operating room to the laboratory in sterile saline on ice and kept at 4 °C until processed.

Isolation of human peripheral blood mononuclear cells (PBMCs) and T cells

For isolation of human PBMCs, 12.5 mL of Lymphoprep density gradient medium was added to 50 mL Falcon tubes, followed by careful overlaying of 30 mL of whole blood diluted 2-fold in phosphate-buffered saline (PBS). Tubes were centrifuged for 20 min at 524 x *g* at room temperature (18-22 °C) with the brake off and acceleration set to 5. Following centrifugation, buffy coats containing white PBMC layer were collected, pooled into sterile Falcon tubes, washed with 15 mL PBS, and centrifuged for 8 min at 520 x *g* (4 °C) with the brake on. Pellets were subsequently combined and resuspended in 5 mL hypotonic red blood cell (RBC) lysis buffer (cat. sc-296258) for 5 min. After this time, tubes were topped up to 50 mL with PBS, centrifuged again, and cells were resuspended in 20 mL resuspension buffer (PBS without calcium or magnesium, supplemented with 2% FBS and 2 mM EDTA). Cell count and viability were assessed using a hemocytometer with Trypan blue staining. T cells were isolated using negative selection EasySep human T cell isolation kits (STEMCELL Technologies) according to slightly modified manufacturer's instructions. Briefly, PBMCs were diluted to 50 million cells per mL in resuspension buffer. The resuspended cells were placed into 14 mL separation tubes (maximum 8 mL/tube), followed by the addition of isolation cocktail (25 µL/mL) and incubation for 5 min at room temperature. A suspension of RapidSpheres (25 µL/mL) was added, mixed by pipetting up and down, and incubated for another 5 min. The cell suspension was then topped up to 10 mL with resuspension buffer and incubated for 5 min on a magnetic rack to remove bead-bound cells. The supernatant containing T cell-enriched cell suspension was transferred into a new 14 mL separation tube on the magnetic rack, followed by incubation for 5 min to completely remove bead-bound cells. The supernatant was then transferred into a 50 mL Falcon using using a serological pipette, after which cells were centrifuged and resuspended in T cell media (RPMI-1640 supplemented with 10% heat-inactivated FBS, 2 mM *L*-glutamine, and 100 U/mL Penicillin-Streptomycin).

Isolation of Tumor infiltrating lymphocytes (TILs) from human renal cell carcinoma tumors.

Tumor infiltrating lymphocytes (TILs) from human tumors were isolated as described before[14]. Briefly, single-cell suspensions from human renal cell carcinoma tumors were generated using Human Tumor Dissociation Kit (Miltenyi Biotec, 130-095-929), following manufacturer's instructions. In brief, tumors were cut into small pieces (~2-4 mm³) and transferred to gentleMACS C tubes (Miltenyi Biotec, 130-093-237) with R-10 medium (RPMI-1640 medium supplemented with 10% heat inactivated FBS, 4 mM *L*-glutamine, 100 U/mL Penicillin-Streptomycin and 10 mM HEPES). Based on the tumor weight, volume of R-10 medium was adjusted and enzyme mix was added. Samples were dissociated with a gentleMACS Octo Dissociator with Heaters (Miltenyi Biotec, 130-134-029) using the 37C_h_TDK3 program for 30 min. Dissociated tissue was then mashed with the flat end of a syringe plunger, passed through a 100 µm strainer, washed with 5 mL of R-10 medium and centrifuged at 500 x *g* for 5 min at 4 °C. Single-cell suspension was resuspended in 1 mL ACK buffer and incubated at RT for 3 min to lyse erythrocytes. Cells were then washed with 9 volumes of R-10 medium, centrifuged at 500 x g for 5 min at 4 °C, and

bioRxiv preprint doi: https://doi.org/10.64898/2026.03.04.709614; this version posted March 6, 2026. The copyright holder for this preprint (which was not certified by peer review) is the author/funder, who has granted bioRxiv a license to display the preprint in perpetuity. It is made available under aCC-BY 4.0 International license.

either cryopreserved in Bambanker freezing medium (Fisher Scientific, NC3072290) or stained for sorting. Cells were stained for viability with 1:400 Ghost Dye Violet (Cell Signaling Technology, 59863S) and 1:20 Human TruStain FcX Fc Receptor Blocking Solution (BioLegend, 422302) in 1X PBS for 10 min on ice. Thereafter, cells were washed with 1X PBS, centrifuged at 350 x *g* for 5 min at 4 °C and stained for surface markers described below with 1X BD Horizon Brilliant Stain Buffer (BD Biosciences, 566349) in FACS buffer (2% FBS in 1 X PBS without calcium and magnesium) for 30 min at 4 °C. Cells were then washed with FACS buffer, spun down at 350 x *g* for 5 min at 4 °C, resuspended in FACS buffer, filtered through a 35 µm nylon mesh (Corning, 352235) and sorted on a SH800S Sony Cell Sorter into tubes containing FACS buffer. Sorted TILs ($CD3^+$ T cells) cells were centrifuged at 520 x *g* for 5 min at 4 °C, washed once with ice cold 1X PBS, and cell pellets were snap frozen on dry ice until further processing for proteomic and Western blot analyses.

Human TILs sort panel

Staining type	Marker	Fluorophore	Catalog #	Clone	Dilution
Surface	CD3	BV570	300436	UCHT1	1:25
Surface	CD56	PE-Cy7	362510	5.1H11	1:50
Surface	CD11b	PE-Cy7	101216	M1/70	1:400
Surface	CD19	PE-Cy7	363012	SJ25C1	1:100
Surface	CD45	BV421	368522	2D1	1:200
Surface	PD1	APC	329908	EH12.2H7	1:100

Mouse T cell isolation

Experiments involving primary mouse cells were performed according to Memorial Sloan Kettering Cancer Center Institutional Animal Care and Use Committee (IACUC) guidelines (protocol number 20-10-012). Mouse T cells were isolated from spleens of 6-12-week-old C57BL/6J mice (Jackson Laboratory 000664). Briefly, mice were euthanized with CO_2 followed by cervical dislocation. Spleens were collected in ice-cold mouse T cell media - RPMI-1640 medium (Media Preparation Core, MSKCC) supplemented with 10% heat inactivated FBS (GeminiBio, 100-106), 4 mM *L*-glutamine, 50 µM β-Mercaptoethanol (ThermoFisher, 21985023), 100 U/mL Penicillin-Streptomycin (ThermoFisher, 15140163). Single cell suspensions of splenocytes were generated by mashing spleens in 5 mL RPMI-1640 medium (Media Preparation Core, MSKCC) with the flat end of a syringe plunger and passing through a 40 µm strainer. Splenocytes were centrifuged at 1,200 rpm for 5 min at 4 °C, resuspended in 5 mL ACK buffer (150 mM NH_4Cl, 10 mM $KHCO_3$, 0.1 mM EDTA-Na_2) and incubated at RT for 90 s to lyse erythrocytes. Cells were filtered through a 40 µm strainer and washed with 45 mL PBS to remove ACK buffer. Splenocytes were resuspended at 1 x 10^8 cells/mL in isolation buffer (1 X PBS without calcium and magnesium containing 2% FBS and 2 mM EDTA) and used for T cell isolation using Dynabeads Untouched Mouse T cell kit (ThermoFisher, 11413D) according to the manufacturer's protocol. Briefly, 500 µL of splenocyte suspension (5 x 10^7 cells) was mixed with 100 µL heat inactivated FBS and 100 µL Antibody Mix and incubated for 20 min at 4 °C. Cells were washed with 10 mL isolation buffer to remove excess of antibody and centrifuged at 1,200 rpm for 5 min at 4 °C. Cells were resuspended in 4 mL isolation buffer, mixed with 1 mL pre-washed Mouse Depletion Dynabeads and incubated for 15 min at RT with gentle tilting and rotation. Thereafter, 5 mL of isolation buffer were added to the cell suspension and tubes were placed in magnets for 2 min. Untouched T cells present in the supernatant were transferred to new tubes, centrifuged at 1,200 rpm for 5 min at 4 °C, resuspended at 1 x 10^6 cells/mL in mouse T cell media supplemented with 10 ng/mL mIL-2 (Peprotech, 212-12), and activated as described in "Generation of acutely or chronically stimulated mouse T cells".

Mouse tumor model and isolation of tumor infiltrating lymphocytes (TILs)

In vivo experiments involving mouse models were performed according to Memorial Sloan Kettering Cancer Center IACUC guidelines (protocol number 20-10-012). Parental 2838c3 KPCY ($Kras^{LSL-G12D/+}$;$Trp53^{LSL-R172H/+}$;Pdx1-Cre;$Rosa26^{YFP/YFP}$) cell line was a kind gift of Dr. Katelyn Byrne[15]. For all

bioRxiv preprint doi: https://doi.org/10.64898/2026.03.04.709614; this version posted March 6, 2026. The copyright holder for this preprint (which was not certified by peer review) is the author/funder, who has granted bioRxiv a license to display the preprint in perpetuity. It is made available under aCC-BY 4.0 International license.

experiments presented here, the KPC cell line was generated by CRISPR Cas9-mediated deletion of YFP (sgRNA: 5'- GTAGCCGAAGGTGGTCACGA-3') using the Lenti-CRISPRv2 plasmid[16] (Addgene, 52961) and further selected by fluorescence-activated cell sorting (FACS). KPC cells were implanted subcutaneasly (2×10^5 cells/mouse in 100 μL 1 X PBS) into the right flank of 8-week-old female C57BL/6J mice (Jackson Laboratory, 000664). When tumors reached an average of 1,500 mm^3, mice were euthanized with CO_2 followed by cervical dislocation, and tumors were harvested to isolate tumor infiltrating lymphocytes (TILs). Additionally, spleens were harvested to isolate T cells not exposed to persistent tumor antigen stimulation. When collected, spleens and tumors were placed in cold RPMI+++ media - RPMI-1640 medium (Media Preparation Core, MSKCC) supplemented with 10% heat inactivated FBS (GeminiBio, 100-106), 4 mM *L*-glutamine, and 100 U/mL Penicillin-Streptomycin (ThermoFisher, 15140163) - until further processing. Spleens and tumors were then cut in small pieces and incubated for 45 min at 37 °C, 250 rpm shaking, in 1-2 mL of digestion buffer containing RPMI-1640 medium supplemented with 100X DNase I (Millipore Sigma, 10104159001; final concentration: 0.1 mg/mL), 350 units/mL hyaluronidase (Worthington Biochemical Corporation, LS002592) and 50 μg/mL Liberase (Millipore Sigma, 05401020001). Single cell suspensions of tumors and spleens were generated by mashing tissues in RPMI+++ media with the flat end of a syringe plunger and passing through a 100 μm strainer (Greiner Bio-One, 542000). Cells were centrifuged at 500 x *g* for 5 min at 4 °C, resuspended in 1-2 mL ACK buffer and incubated at RT for 5 min to lyse erythrocytes. Cells were then washed with 9 volumes of 1 X PBS to quench ACK buffer, centrifuged at 500 x *g* for 5 min at 4 °C, and proceeded with Dynabeads FlowComp Mouse Pan T (CD90.2) Kit (FisherScientific, 11465D) following manufacturer's instruction to enrich for T cell population. After enrichment, cells were stained on ice for viability with 1:2000 Ghost Dye Red 780 (Cytek Biosciences, 13-0865-T100) and 1:100 TruStain FcX (BioLegend, 101320) in 1X PBS for 10 min. Following centrifugation at 300 x *g* for 5 min at 4 °C, cells were stained for surface markers described below with 1X BD Horizon Brilliant Stain Buffer (BD Biosciences, 566349) in FACS buffer for 30 min at 4 °C, then washed with FACS buffer and spun down at 300 x *g* for 5 min at 4 °C. Cells were then resuspended in FACS buffer and sorted on a BD FACSymphony S6 Cell Sorter into tubes containing FACS buffer. Sorted cells were centrifuged at 520 x *g* for 5 min at 4 °C, washed once with ice cold 1X PBS, and cell pellets were snap frozen on dry ice until further processing for proteomic and Western blot analyses.

Spleen and TILs sort panel

Staining type	Marker	Fluorophore	Catalog #	Clone	Dilution
Surface	CD45	BV570	103136	30-F11	1:800
Surface	CD11b	BUV395	563553	M1/70	1:800
Surface	NK1.1	BUV395	564144	PK136	1:200
Surface	CD45R/ B220	BUV395	563793	RA3-6B2	1:800
Surface	TCRb	BUV805	748405	H57-597	1:100
Surface	CD8	BUV615	613004	53-6.7	1:800
Surface	CD4	PE	100408	GK1.5	1:800
Surface	CD44	FITC	11-0441-85	IM7	1:400
Surface	PD1	PE-Cy7	109110	RMP1-30	1:200

Lymphocytic choriomeningitis virus (LCMV) infection and isolation of virus reactive mouse T cells

Lymphocytic choriomeningitis virus (LCMV) was obtained from the Gene Transfer, Targeting, and Therapeutics Core, Salk Institute for Biological Studies, La Jolla, CA, USA. C57BL/6J female mice (10-week-old) were infected with LCMV Armstrong (2×10^5 PFU/mouse in 100 μL 1 X PBS, via intraperitoneal injection) or Clone 13 (2×10^6 PFU/mouse in 100 μL 1 X PBS, via retro-orbital injection). Spleens from LCMV-infected mice were harvested on day 8 (Armstrong and Clone 13) and day 15 (Clone 13) post-infection. At experiment endpoint, mice were euthanized with CO_2 followed by cervical dislocation, and spleens were collected to isolate virus-reactive T cells. When collected, spleens were placed in cold

bioRxiv preprint doi: https://doi.org/10.64898/2026.03.04.709614; this version posted March 6, 2026. The copyright holder for this preprint (which was not certified by peer review) is the author/funder, who has granted bioRxiv a license to display the preprint in perpetuity. It is made available under aCC-BY 4.0 International license.

RPMI+++ media until further processing. Single cell suspensions of spleens were generated by mashing tissues in RPMI+++ media with the flat end of a syringe plunger and passing through a 100 μm strainer. Cells were centrifuged at 500 x *g* for 5 min at 4 °C, resuspended in 1 mL ACK buffer and incubated at RT for 3 min to lyse erythrocytes. Cells were then washed with 9 volumes of 1 X PBS to remove ACK buffer, centrifuged at 500 x *g* for 5 min at 4 °C, and proceeded with Dynabeads Untouched Mouse CD8 Cells Kit (ThermoFisher, 11417D) following manufacturer's instruction to enrich for $CD8^+$ T cells. After enrichment, cells were stained on ice for viability staining with 1:2000 Ghost Dye Red 780 (Cytek Biosciences, 13-0865-T100) and 1:100 TruStain FcX (BioLegend, 101320) in 1X PBS for 10 min. Following centrifugation at 300 x *g* for 5 min at 4 °C, cells were stained for surface markers described below with 1X BD Horizon Brilliant Stain Buffer (BD Biosciences, 566349) in FACS buffer for 30 min at 4 °C, then washed with FACS buffer and spun down at 300 x *g* for 5 min at 4 °C. Cells were then resuspended in FACS buffer and sorted on a BD FACSAria III Cell Sorter into tubes containing FACS buffer. Sorted cells were centrifuged at 520 x *g* for 5 min at 4 °C, washed once with ice cold 1X PBS, and cell pellets were snap frozen on dry ice until further processing for proteomic and Western blot analyses.

LCMV T cell sort panel

Staining type	Marker	Fluorophore	Catalog #	Clone	Dilution
Surface	TCRb	PerCP/Cy5.5	109228	H57-597	1:100
Surface	CD8	APC	100712	53-6.7	1:800
Surface	CD4	BV711	100550	GK1.5	1:800
Surface	CD44	PE	12-0441-83	IM7	1:400
Surface	PD1	PE-Cy7	109110	RMP1-30	1:200

METHODS DETAILS

T cell activation (D2 activated T cells)

For T cell activation, untreated 15 cm² culture dishes were pre-coated with 17 mL of a solution of anti-CD3 antibody (5 μg/mL, diluted 1:200 from a 1 mg/mL stock; original stock concentration: 6.66 mg/mL) and anti-CD28 antibody (2 μg/mL; stock concentration: 7.3 mg/mL) in PBS. Plates were incubated overnight at 4 °C or, alternatively, for 2 h at 37 °C. Prior to cell plating, the coating solution was aspirated, and plates were washed twice with 5 mL of PBS. Primary human T cells, isolated by negative selection as described above, were resuspended in T cell media supplemented with IL-7 and IL-15 at final concentrations of 5 ng/mL each (5 μL of each stock per 100 mL of media). On day 0, T cells were plated at a density of 1×10^6 cells/mL (20×10^6 cells per 15 cm² plate) and incubated for 48 h at 37 °C and 5% CO_2 for activation before being allocated to conditions with and without chronic stimulation.To prepare cytokines for T cell culture *in vitro*, human recombinant IL-7 and IL-15 (STEMCELL Technologies) were reconstituted at 100 μg/mL by dissolving 100 μg of lyophilized powder in 1 mL of 0.1% BSA in molecular-grade water. Aliquoted stock solutions were stored at -80 °C until use.

Generation of acutely and chronically stimulated primary human T cells (D4A, D8A, D15A, D4C, D8C, D15C)

On day 2, activated T cells were harvested by pipetting up and down multiple times to ensure complete detachment from the plate. Cells were spun down (520 x *g*, 5 min, 4 °C), counted using trypan blue exclusion, and resuspended in fresh T cell media containing IL-7 and IL-15 at a density of 1×10^6 cells/mL. Cells (20×10^6) were plated on 15 cm² dishes, which have either been pre-coated with anti-CD3 antibody (chronic stimulation; 5 μg/mL in PBS, 200x stock) or used without pre-coating (acute stimulation). Every 48 hours, all cells from each respective condition were pooled, counted, and resuspended in fresh media containing IL-7 and IL-15 at 1×10^6 cells/mL. Cells were replated at 20×10^6 cells per 15 cm² dish, either on anti-CD3 pre-coated plates or uncoated plates, following the same conditions as day 2. This subculture procedure was repeated every two days to maintain consistent experimental conditions with the exception of the last timepoint, which was collected one day after re-plating (D15A/D15C).

bioRxiv preprint doi: https://doi.org/10.64898/2026.03.04.709614; this version posted March 6, 2026. The copyright holder for this preprint (which was not certified by peer review) is the author/funder, who has granted bioRxiv a license to display the preprint in perpetuity. It is made available under aCC-BY 4.0 International license.

Generation of acutely or chronically stimulated mouse T cells

Generation of acutely or chronically stimulated mouse T cells was performed as previously described[3]. Briefly, freshly isolated T cells were seeded at 1 x 10^6 cells/mL and activated with plate-bound anti-CD3 (3 µg/mL, ThermoFisher, 16-0031-38) and anti-CD28 (1 µg/mL, ThermoFisher, 16-0281-85) for 48 h at 37 °C and 5% CO_2 in mouse T cell media supplemented with 10 ng/mL mIL-2 (Peprotech, 212-12), henceforth referred to as 'complete mouse T cell media'. On day 2, following 48 h of activation, activated T cells were harvested by pipetting up and down multiple times to ensure complete detachment from the plate. Cells were spun down (300 x *g*, 5 min, 4 °C), counted using a Beckmann Coulter Multisizer 3 Particle Size Analyzer with a gate of between 200 and 1000 femtoliters (fL), and resuspended in fresh complete mouse T cell media at a density of 1 × 10^6 cells/mL. Cells (2 × 10^6) were plated in 6-well plates, which had either been pre-coated with anti-CD3 antibody (3 µg/mL in PBS, ThermoFisher, 16-0031-38) or used without pre-coating (acute stimulation). Every 48 h, all cells from each respective condition were pooled, counted, and resuspended in fresh complete mouse T cell media at 1 × 10^6 cells/mL. Cells were replated at 2 × 10^6 cells per well of a 6-well plate, either on anti-CD3 pre-coated plates or uncoated plates, following the same conditions as day 2. This subculture procedure was repeated every two days to maintain consistent experimental conditions. Cell density was kept below 5 × 10^6 cells/mL on each passage.

Flow cytometry analysis

Inhibitory receptor panel

Cells were plated at 200,000 live cells per well in a U-bottom 96-well plate. Cells were spun at 520 x *g* for 3 min at 4 °C and washed with 1% BSA in 1X PBS. Cells were incubated with Fc block (cat. 422302) at a 1:20 dilution for 30 min at 4 °C. Following centrifugation, cells were stained for surface markers with 1X Brilliant Buffer (cat. 566349) in 1% BSA in PBS for 30 min at 4 °C, then washed with 1% BSA in PBS and spun down. Viability staining was performed for 30 min at 4 °C using Ghost Dye 510 Violet (cat. 59863) diluted 1:800 in PBS. Following washing and centrifugation, cells were fixed for 30 min at room temperature using the eBioscience Intracellular Fixation & Permeabilization Buffer Set (cat. 88-8824-00). Fixed cells were centrifuged at 700 x *g* for 5 min and washed twice with permeabilization buffer. Cells were stained overnight at 4 °C for two of the intracellular markers, T-Bet and TCF-1, diluted in permeabilization buffer. The following day, cells were spun down and resuspended in stains for two additional markers (CTLA4 and TOX) for a further 30 min at 4 °C. Cells were washed twice in permeabilization buffer and resuspended in 1% BSA in PBS. Data was acquired using a Cytek Aurora spectral cytometer, with a minimum of 5,000 live CD8 cells measured per condition. Gating for CD4 and CD8 was determined by eye, while all other gates were set using fluorescence minus one (FMO) controls.

Staining type	Marker	Fluorophore	Catalog #	Clone	Dilution
Surface	CD4	BUV395	363-0047-42	SK3	1:200
Surface	CD8	BUV496	612942	RPA-T8	1:800
Surface	CD38	BUV737	612825	HB7	1:400
Surface	CD39	PE-Fire 810	568712	A1	1:200
Surface	HLA-DR	BV786	564041	G46-6	1:200
Surface	PD-1	APC	329908	EH12.2H7	1:100
Surface	LAG3	BV421	369314	11C3C65	1:100
Surface	TIGIT	BUV615	570444	TgMab-2	1:50
Intracellular (O/N)	T-Bet	R718	568175	4B10	1:200
Intracellular (O/N)	TCF-1	Alexa 488	6444S	C63D9	1:3,200
Intracellular (30 m)	CTLA4	BV711	369632	BNI3	1:400
Intracellular (30 m)	TOX	PE	130-120-716	REA473	1:50

bioRxiv preprint doi: https://doi.org/10.64898/2026.03.04.709614; this version posted March 6, 2026. The copyright holder for this preprint (which was not certified by peer review) is the author/funder, who has granted bioRxiv a license to display the preprint in perpetuity. It is made available under aCC-BY 4.0 International license.

Cytokine panel

Experiments were performed as above with the following differences: Upon cell counting and plating, cells were resuspended in T cell media containing phorbol 12-myristate 13-acetate (PMA, cat. P1585, 50 ng/mL) and ionomycin (cat. I0634, 1 µg/mL). After 1 h, Brefeldin A (cat. 00-4506-51, 1:1,000 dilution) was added to the media for an additional 3 h incubation at 37 °C. Blocking, surface and viability staining was performed as for the inhibitory receptor panel. Fixation was performed using the BD Biosciences Cytofix/Cytoperm kit (cat. 554714) for 30 min at 4 °C. All intracellular staining was performed overnight.

Staining type	Marker	Fluorophore	Catalog #	Clone	Dilution
Surface	CD4	BUV395	363-0047-42	SK3	1:200
Surface	CD8	BUV496	612942	RPA-T8	1:800
Surface	PD-1	APC	329908	EH12.2H7	1:100
Intracellular	GzmB	PerCP-Cy5.5	372212	QA16A02	1:200
Intracellular	IFNγ	FITC	502506	4S.B3	1:200
Intracellular	IL-2	APC-Fire 750	500352	MQ1-17H12	1:400
Intracellular	TNF	PE	502909	MAb11	1:1,600

Restimulation flow on inhibitor-treated samples

To evaluate function under more physiologically relevant restimulation conditions, we used ImmunoCult Human CD3/CD28/CD2 T Cell Activator beads (cat. 10970) at a concentration of 12.5 µL/mL. After 1 hour, Brefeldin A (cat. 00-4506-51, 1:1,000 dilution) was added to the media for an additional 5 h incubation at 37 °C. All other steps were performed as for the "Cytokine panel" experiments.

Cell cycle analysis

The cell cycle flow cytometry assay was performed following a protocol adapted from the Click-iT Plus EdU Alexa Fluor 594 Flow Cytometry Assay Kit (cat. C10646). Cells were incubated with EdU at a concentration of 10 µM in 200 µL T cell media for 30 min at 37 °C. After incubation, cells were spun down for 3 min at 520 x *g* and washed with 1% BSA in PBS. The cells were then stained with Zombie NIR live/dead stain (cat. 423106, dilution 1:400) for 10 min at 4 °C and washed with 1% BSA in PBS. Cells were fixed in 3.7% formaldehyde (cat. F79-1) for 15 min at room temperature, then washed with saponin-based permeabilization buffer. Following fixation, centrifugations were increased to 5 min at 700 x *g*. Cells were incubated with Fc block (cat. 422302) at a 1:50 dilution in saponin-based permeabilization wash for 15 min at 4 °C. Staining for CD4 (Brilliant Violet 711, cat. 563028, dilution 1:200) and CD8 (FITC, cat. 947296, dilution 1:400) was then performed intracellularly. Cells were washed with permeabilization wash, spun down and incubated in 50 µL per well of the Click-iT reaction cocktail, including Alexa Fluor 594 picolyl azide and copper protectant for 30 min at room temperature. Following the reaction, cells were washed twice with permeabilization buffer, then resuspended in 1% BSA in PBS containing FxCycle Violet (cat. F10347) at a dilution of 1:50,000. Flow cytometry data was acquired on a Cytek Aurora at low flow rate. FxCycle Violet signal was processed as autofluorescence for the purpose of unmixing. Samples were stained in triplicate and a matched replicate not treated with EdU used to gate each sample.

TCR transduction confirmation

To confirm successful transduction of primary human T cells with the 1G4 TCR, single-cell suspensions were first stained with Ghost Dye 510 (see Key Resources Table) and Human FcBlock (Human TruStain FcX) in a volume of 50 µL of PBS for 10 min in the dark at room temperature (RT). Cells were then washed with 200 µL of FACS buffer and centrifuged at 700 x *g* for 2 min at 4 °C. Cells were then resuspended in 50 µL of surface stain containing 1x Brilliant Buffer in FACS buffer and the below panel for 20 min at RT in the dark. Following surface staining, cells were washed once with FACS buffer and resuspended in a final volume of 200 µL. Immediately prior to acquisition, cell suspensions were passed through a 100 µm nylon mesh filter to ensure a single-cell suspension. Data were acquired on a Cytek Aurora. An untransduced culture from the same donor was run simultaneously to gate for the true 1G4

bioRxiv preprint doi: https://doi.org/10.64898/2026.03.04.709614; this version posted March 6, 2026. The copyright holder for this preprint (which was not certified by peer review) is the author/funder, who has granted bioRxiv a license to display the preprint in perpetuity. It is made available under aCC-BY 4.0 International license.

TCR (identified by Mouse TCR β Chain) positive population and this percentage was used as the transduction efficiency to plate the Incucyte-based tumor cell killing assays.

Staining type	Marker	Fluorophore	Catalog #	Clone	Dilution
Surface	Human CD4	BV711	300558	RPA-T4	1:800
Surface	Human CD8	BUV563	612914	RPA-T8	1:3200
Surface	Mouse TCR β Chain	BUV805	748405	H57-597	1:100

Oxidative stress assays

To assess oxidative stress in primary human and mouse T cells, we used dihydroethidium (DHE) and MitoSOX Red (MSR) as indicators of whole cell superoxide accumulation. Stock solutions were freshly prepared as follows: DHE was reconstituted at 5 mM in DMSO, Antimycin A (AA) at 10 mM in ethanol, and MSR at 1 mM in DMSO. Working solutions were prepared in PBS containing 5 mM pyruvate, with final concentrations of 0.625 µM DHE, 1.64 µM MSR, and 10 µM AA.

Cells were seeded at 2×10^5 cells per well in a 96-well U-bottom plate and centrifuged at 520 × g for 5 min. After washing with PBS, cells were incubated with a blocking buffer containing an Fc receptor blocker (cat. 422302, 1:20) and Ghost Dye Violet 510 (cat. 59863S, 1:800) for 10 min at room temperature. Following another PBS wash, surface staining was performed by incubating cells with BUV395-conjugated CD8 antibody (1:200) along with superoxide detection dyes (DHE or MSR) in the presence or absence of AA for 30 min at 37 °C in a humidified incubator, protected from light. Staining conditions included unstained controls, single-color controls, fluorescence-minus-one (FMO) controls, and a positive control with AA treatment. After surface staining and oxidative stress dye incubation, cells were washed with ice-cold PBS, resuspended in 200 µL ice-cold FACS buffer, kept on ice, preventing cells from light until analysis. Flow cytometry was performed using a Cytek Aurora, with acquisition gated on $CD8^+$ T cells and a stopping gate of 1,500 events per sample. Data were analyzed using FlowJo.

Extracellular flux analysis

Oxygen consumption rate (OCR), extracellular acidification rate (ECAR), and ATP production rates were measured using a Seahorse XFe96 Extracellular Flux Analyzer (Agilent). Primary human T cells were isolated, activated, and cultured as described above. Activated (D2), acutely, and chronically stimulated T cells (D8A, D8C) from the same donor were analyzed on the same plate. To enable this, freshly isolated T cells were cryoprezerved in Bambanker freezing medium at -80 °C until day 6 of cell culture. At that time, the cells were thawed, spun down to remove the freezing medium (92 x *g*, 10 min), re-suspended in T cell culture medium, and activated for 2 days, allowing D2 cells to be analyzed in parallel with D8A and D8C cells from the same donor. One day before the analysis, XFe96/XF Pro cell culture microplates (Agilent, 103794-100) were coated with 20 µL of 50 µg/mL poly-*L*-lysine (Millipore Sigma, P4707) in 1X PBS and incubated for 30 min at RT. Plates were washed twice with 100 µL of 1X PBS, air dryed for 1-2 h and stored overnight at 4 °C. On the day of the analsysis, T cells were spun down at 300 x *g* for 5 min at 4 °C and washed once with Seahorse medium (XF RPMI medium, 10 mM XF glucose, 1 mM XF pyruvate, and 2 mM *L*-glutamine). T cells were then plated at 1 x 10^5 per well in Seahorse medium on XFe96/XF Pro poly-*L*-lysine-coated plates and centrifuged at 300 x *g* for 1 min at 4 °C. Cells were incubated in a non-CO_2 incubator at 37 °C for 45-60 min before the analysis. OCR and ECAR were measured at basal level and after treatment with oligomycin (1 µM), FCCP (1 µM), and rotenone/antimycin mix (0.5 µM), contained in the Seahorse XF Cell Mito Stress Test Kit (Agilent, 103015-100). OCR, ECAR, and ATP production rates were determined using Agilent Seahorse Analytics XF software.

Incucyte-based tumor cell killing assay[17]

1G4-LY TCR transfer plasmids

The HLA-A*02:01-restricted NY-ESO-$1_{157\text{-}165}$-specific TCR 1G4 has been described previously[18]. Human constant regions of the 1G4 TCR were replaced with codon optimized murine constant regions[19]

bioRxiv preprint doi: https://doi.org/10.64898/2026.03.04.709614; this version posted March 6, 2026. The copyright holder for this preprint (which was not certified by peer review) is the author/funder, who has granted bioRxiv a license to display the preprint in perpetuity. It is made available under aCC-BY 4.0 International license.

harboring a second disulfide bond[20] as previously described. Codon optimized sequences encoding a bicistronic 1G4 TCR construct were synthesized by Genscript and subcloned into the lentiviral transfer plasmid pZR071[21] (Addgene, #180264) via restriction enzyme cloning using MluI-HF (NEB, #R3198S) and SbfI-HF (NEB, #R3642S). The affinity enhanced 1G4-LY[18] was generated via site directed mutagenesis (NEB, #E0554S). Final sequences were verified by whole plasmid sequencing (Plasmidsaurus).

Tumor cell lines

For repetitive cytotoxicity assays, A375 (ATCC, CRL-1619) and SK-MEL-37 (Antibody and Bioresource Core Facility at MSKCC and RU, CVCL_3878) melanoma cells, were retrovirally transduced with nuclear localized mCherry (pMSGV1_mCherry-NLS_T2A_blastR), selected with blasticidin (10 µg/mL) and either used as bulk population (SK-MEL-37) or single cell cloned by limiting dilution (A375). The NLS is derived from *c*-MYC (PAAKRVKLD) and attached to the *C*-terminus of mCherry. The T2A sequence is preceded by a furin cleavage site (RAKR). The change from puroR to blastR was done by Genscript.

Lentivirus Production and Concentration

Lentiviral production was performed according to previously published protocols, with minor modifications.[21,22] Lentiviral vectors were generated by co-transfecting Lenti-X 293T cells (Takara, cat #632180) with a third-generation packaging system. Briefly, Lenti-X 293T cells were maintained in DMEM supplemented with 10% FBS and seeded at a density of 3-3.5 x 10^6 cells per 10 cm dish in cOPTIMEM (OPTIMEM supplemented with 5% FBS, 2 mM *L*-glutamine, 1x MEM non-essential amino acids (Gibco #11140-050), and 1 mM sodium pyruvate (Gibco #11360-070) 16-24 h prior to transfection to reach 70-90% confluency. Transfection was performed using Lipofectamine 3000 (Thermo Fisher Scientific) according to the manufacturer's instructions. For a 10 cm dish, a total of 21.25 µg of DNA, comprising the transfer plasmid, psPAX.2 (Addgene #12260), and pMD2.G (Addgene #12259) at a 1:1:1 molar ratio, was mixed with P3000 reagent in Opti-MEM and added to the Lenti-X 293T cells. When other culture formats were used, reagent volumes and amounts were scaled proportionally by surface area. Three to six hours post-transfection, 1x Viral Boost (Alstem #VB100) was added.

Viral supernatants were harvested at 24 and 48 h post-transfection. The harvests were combined and cleared of debris by centrifugation (500 x *g* for 5 min) and filtered through a 0.45 µm PES filter. The virus was concentrated using Lentivirus Precipitation Solution (Alstem #VC100) at a 1:4 (v/v) ratio by incubating for a minimum of 3 h to overnight at 4 °C and centrifuged at 1,500 x *g* for 30 min. The resulting viral pellets were resuspended in cold RPMI at a 1/100 of the original volume and either used immediately or aliquoted and stored at -80 °C.

Primary Human T Cell Transduction

Primary human bulk T cells ($CD3^+$) were isolated from healthy donor PBMCs by negative selection and activated as described above. Twenty-four hours after initiating activation, T cells were transduced with concentrated lentivirus at a final volume of 2% (v/v) by pre-diluting the 100x viral stocks at 1:5 in T cell medium before addition to the culture. After 24 h of viral exposure, cells were centrifuged and resuspended in fresh RPMI+++ media supplemented with 5 ng/mL hIL-7 (Peprotech, 200-07) and hIL-15 (Peprotech, 200-15). Successful transduction was confirmed by flow cytometry.

Cell killing assay[17]

Tumor cells stably expressing a nuclear-localized fluorescent reporter (A375 mCherry-NLS or SK-MEL37 mCherry-NLS) were seeded into flat-bottom 96-well plates (Corning 3599) at 1 × 10^4 cells per well in 100 µL RPMI supplemented with 10% fetal bovine serum and 1% penicillin–streptomycin (RPMIc). Cells were allowed to adhere for ≥3 h before baseline imaging. Plates were loaded onto an Incucyte live-cell imaging system and scanned using a 10× objective, acquiring phase contrast and red fluorescence signal with preset acquisition parameters. Four images per well were collected at 3-4 h intervals. Plates were equilibrated for at least 30 min prior to imaging to minimize condensation artifacts.

After baseline acquisition, plates were removed from the Incucyte and T cells were added in 100 µL RPMIc at the indicated effector-to-target (E:T) ratios, accounting for TCR transduction efficiency as determined by flow cytometry as described above on the day of assay setup. Conditions were plated in

bioRxiv preprint doi: https://doi.org/10.64898/2026.03.04.709614; this version posted March 6, 2026. The copyright holder for this preprint (which was not certified by peer review) is the author/funder, who has granted bioRxiv a license to display the preprint in perpetuity. It is made available under aCC-BY 4.0 International license.

technical triplicates. Control wells included tumor cells alone and tumor cells co-cultured with untransduced T cells. Plates were reloaded into the Incucyte at least 30 min prior to imaging to minimize condensation artifacts.

Every 48 h thereafter, 75 µL of medium was carefully removed from each well without disturbing the cell layer, and fresh tumor cells (1×10^4 per well in 100 µL RPMIc) were added. Longitudinal imaging was continued throughout the assay under identical acquisition settings.

RNA sequencing of activated, acutely, and chronically stimulated human T cells

Sample preparation

Primary human T cells were activated and expanded with or without chronic stimulation as described above. Following 2, 4, and 8 days of activation, biological triplicate wells were harvested and RNA was extracted using a TRIzol-chloroform-based method[23]. After RiboGreen quantification and quality control by Agilent BioAnalyzer, 500 ng of total RNA with RIN values of 9.4-10 underwent polyA selection and TruSeq library preparation according to instructions provided by Illumina (TruSeq Stranded mRNA LT Kit, catalog # RS-122-2102), with 8 cycles of PCR. Samples were barcoded and run on a NovaSeq 6000 generating 100-bp paired-end reads, using the NovaSeq S4 Reagent Kit (200 Cycles) (Illumina). An average of 127 million paired reads was generated per sample. Ribosomal reads represented 0.1-1.2% of the total reads generated and the percent of mRNA bases averaged 88%. RNA sequencing was performed at the Integrated Genomics Operation Core, MSKCC.

Data processing

FASTQ files were quality-checked using FastQC v0.12.0 and trimmed with Trim Galore v0.6.10 to remove adapter sequences and read ends with Phred scores below 15. STAR v2.7.0a was used to align the FASTQ files to the hg38 reference genome. SAM files outputted by STAR were sorted using the sort function from Samtools v1.19.2 and indexed using the index function to generate BAM index files. A gene expression count matrix was generated using featureCounts v2.0.6, quantifying only read pairs that overlapped exons. Downstream differential gene expression analysis was performed using DESeq2 v1.42.

Principal Component Analysis

Principal component analysis (PCA) was performed using *sci-kit learn*[24] library for Python. The 5,000 protein-coding genes with the highest row-wise variance were used and normalized count values were $\log_2$ transformed before PCA. The first two principal components (PC1 and PC2) were plotted.

Gene Set Enrichment Analysis

The bulk RNA-Seq dataset was filtered to genes with a sum of least 1,000 reads across 15 samples (14,391 genes) for GSEA. Genes were ranked based on $\log_2$(FC) and the permutation type was set to gene-set.

Single-cell RNA sequencing of acutely and chronically stimulated primary human T cells (D15A, D15C)

Sample preparation

Primary human T cells were isolated and cultured as described above ('Generation of acutely and chronically stimulated primary human T cells'). On day 15 following initial activation, acute and chronic T cells were collected and stained with a viability dye along with anti-CD4 (RPA-T4, BioLegend 300534, 1:100) and anti-CD8 (RPA-T8, BD Biosciences 612914, 1:1,200). Live $CD4^+$ and $CD8^+$ T cells from acute and chronic conditions were flow sorted and underwent single cell gene expression profiling using the 10x Genomics Next GEM Single Cell 3' Gene Expression Kit, v3.1. Library preparation from cDNA was performed using the MAS-Seq Kit (PacBio) followed by Illumina sequencing.

MAS-ISO-seq Data Preprocessing

PacBio HiFi reads were first segmented using the *split* function in Skera (v1.3.0). Primers were then removed using lima v2.12.0. With Iso-Seq (v4.0.0), tags (UMI, cell barcode) were clipped from reads

bioRxiv preprint doi: https://doi.org/10.64898/2026.03.04.709614; this version posted March 6, 2026. The copyright holder for this preprint (which was not certified by peer review) is the author/funder, who has granted bioRxiv a license to display the preprint in perpetuity. It is made available under aCC-BY 4.0 International license.

using the *tag* function with the design parameter set as *'T-12U-16B'*, reads were refined using the *refine* function with the *'--require-polya'* parameter, single cell barcodes were corrected with the *correct* function using the '*3M-februrary-2018-REVERSE_COMPLEMENTED.txt'* barcode inclusion list, and reads were deduplicated with the *groupdedup* function. Following this, reads were aligned to GrCh38/hg38 with the pbmm2 (v1.16.99) *align* function with the *'--preset ISOSEQ*' parameter. Transcripts were quantified for each sample utilizing IsoQuant (v3.3.1) with transcript annotations from GENCODE GrCh38v39 and the parameter *'--data_type pacbio_ccs'*. Custom scripts were then used to remove reads with mapq < 5 and reads that were labeled *'noninformative'* or *'intergenic'* by IsoQuant. The remaining reads were assigned to genes, and read assignments were then converted into a cell barcode to gene counts matrix. Additionally, a library size for each cell was calculated as the number of mapped reads in each cell.

The counts matrix was loaded into a Seurat object using the *CreateSeuratObject* function in Seurat (v5.1.0). The counts was normalized by multiplying by 10,000/(cell library size) and then log transformed with the *log1p* function. Cells were initially filtered such that cells with ≤100 expressed genes and cells with ≤100 total counts were removed. Additionally, genes were filtered such that genes expressed in ≤10 cells were removed. Next, cells were filtered based on total counts excluding ribosomal and mitochondrial genes. Cells with less than $2^{10.3}$ to $2^{10.75}$ non-ribosomal/mitochondrial counts were removed, with the exact cutoff determined on a per sample basis by visualization of count density. Cells were then filtered such that those with greater than 9 to 12 percent mitochondrial content were removed on a per sample basis. Doublet Detection v4.2 was used to annotate doublets with the parameter *'voter_thresh=0.5'* for the *predict* function. After running *FindVariableFeatures* and *ScaleData* (default parameters), PCA was conducted using *RunPCA* with variable features as the *'features'* parameter, and a neighborhood graph was constructed with *FindNeighbors* with '*dims = 1:15'*. Leiden clusters were computed for each sample with *FindClusters* (1.5 resolution). Leiden clusters with ≥25% cells assigned as doublets were removed. The samples were then combined into a single object, and visualized with a UMAP created by running *FindVariableFeatures*, *ScaleData* (default parameters), *RunPCA* (using variable features), *FindNeighbors* with '*dims = 1:15', FindClusters* (1.0 resolution), and *RunUMAP* with '*dims = 1:15'*.

$CD8^+$ T cell subtype annotations from a Pan-cancer T cell atlas[2] were transferred onto the *in vitro* $CD8^+$ T cell scRNA-seq samples by first determining anchors between the two datasets with the *FindTransferAnchors* function with parameters *'reference = [atlas data], query = [in vitro data], dims=1:30, reference.reduction="pca''. TransferData* function was called to generate predicted cell types for the *in vitro* data from the anchors using cell type annotations from the atlas as the reference data.

Metabolomic analysis

Sample preparation

For lipid and polar metabolomic analysis, activated, acutely, and chronically stimulated human T cells were harvested at the specified days following initial activation. Cells were washed with cold PBS twice, pelleted, and supernatant was removed. Cell pellets were snap frozen and stored at -80 °C until analysis. Lipid and polar metabolite analysis were performed at Weill Cornell Medicine Proteomics and Metabolomics Core Facility.

Lipid analysis

Lipids were extracted from samples as previously described[25]. The extract was dried down using a SpeedVac and then reconstituted using acetonitrile/isopropanol/water 65:30:5 prior to LC-MS/MS analysis. Chromatographic separation was performed on a Vanquish UHPLC system with a Cadenza CD-C18 3 µm packing column (Imtakt, 2.1 mm id x 150 mm) coupled to a Q Exactive Orbitrap mass spectrometer (Thermo Scientific) via an Ion Max ion source with a HESI II probe (Thermo Scientific). The mobile phase consisted of buffer A: 60% acetonitrile, 40% water, 10 mM ammonium formate with 0.1% formic acid and buffer B: 90% isopropanol, 10% acetonitrile, 10 mM ammonium formate with 0.1% formic acid. The LC gradient was as follows: 0-1.5 min, 32% buffer B; 1.5-4 min, 32-45% buffer B; 4-5 min, 45-52% buffer B; 5-8 min, 52-58% buffer B; 8-11 min, 58-66% buffer B; 11-14 min, 66-70% buffer B; 14-18 min, 70-75% buffer B; 21-25 min, isocratic 97% buffer B, 25-25.1 min 97-32% buffer B; followed by 5 min

bioRxiv preprint doi: https://doi.org/10.64898/2026.03.04.709614; this version posted March 6, 2026. The copyright holder for this preprint (which was not certified by peer review) is the author/funder, who has granted bioRxiv a license to display the preprint in perpetuity. It is made available under aCC-BY 4.0 International license.

of re-equilibration of the column before the next run. The flow rate was 200 µL/min. A data-dependent mass spectrometric acquisition method was used for lipid identification. In this method, each MS survey scan was followed by up to 10 MS/MS scans performed on the most abundant ions. Data was acquired in both positive mode and negative mode. The following electrospray parameters were used: spray voltage 3.0 kV, heated capillary temperature 350 °C, HESI probe temperature 350 °C, sheath gas, 35 units; auxiliary gas, 10 units. For MS scans: resolution, 70,000 (at m/z 200); automatic gain control target, 3e6; maximum injection time, 200 ms; scan range, 250-1800 m/z. For MS/MS scans: resolution, 17,500 (at 200 m/z); automatic gain control target, 1e5 ions; maximum injection time, 75 ms; isolation window, 1 m/z; NCE, stepped 20, 30, and 40. LC-MS files were processed using MS-DIAL software[26] for lipid identification and relative quantitation.

Polar metabolite analysis

The sample was extracted using pre-chilled 80% methanol (-80 °C). The extract was dried with a SpeedVac, and redissolved in HPLC grade water before it was applied to the hydrophilic interaction chromatography LC-MS. Metabolites were measured on a Q Exactive Orbitrap mass spectrometer (Thermo Scientific), which was coupled to a Vanquish UPLC system (Thermo Scientific) via an Ion Max ion source with a HESI II probe (Thermo Scientific). A Sequant ZIC-pHILIC column (2.1 mm i.d. × 150 mm, particle size of 5 µm, Millipore Sigma) was used for separation of metabolites. A 2.1 × 20 mm guard column with the same packing material was used for protection of the analytical column. Flow rate was set at 150 µL/min. Buffers consisted of 100% acetonitrile for mobile phase A, and 0.1% NH_4OH/20 mM CH_3COONH_4 in water for mobile phase B. The chromatographic gradient ran from 85% to 30% A in 20 min followed by a wash with 30% A and re-equilibration at 85% A. The Q Exactive was operated in full scan, polarity-switching mode with the following parameters: the spray voltage 3.0 kV, the heated capillary temperature 300 °C, the HESI probe temperature 350 °C, the sheath gas flow 40 units, the auxiliary gas flow 15 units. MS data acquisition was performed in the m/z range of 70–1,000, with 70,000 resolution (at 200 m/z). The AGC target was 1e6 and the maximum injection time was 250 ms. The MS data was processed using XCalibur 4.1 (Thermo Scientific) to obtain the metabolite signal intensities. Identification required exact mass (within 5 ppm) and standard retention times.

Data processing and imputation

Within each biological replicate, metabolites were required to have at least one condition with an average signal intensity above 5,000 (174/203 metabolites passed). After filtering and log transformation, missing data was imputed through quantile regression imputation of left-censored data (QRILC) performed using imputeLCMD version 2.1. Channel ratios (signal intensity / sum of signal intensities per metabolite) were calculated and used for all analyses.

Principal component analysis

Metabolite ratio values were $\log_2$ transformed and only metabolites with a channel ratio above 0 in all channels before imputation were used. The first and third principal components (PC1 and PC3) were plotted for polar metabolite data. The first two principal components (PC1 and PC2) were plotted for lipidomic data. Principal component analysis was performed with *scikit-learn*[24] version 1.4.2 for Python version 3.1.1. Loadings for plotting were selected by taking the 5 metabolites with highest and lowest loadings for both principal components.

Hierarchical clustering

Only metabolites quantified in all replicates were used for hierarchical clustering (173/174). A z-score was calculated for each observation by $z = (x-\mu)/\sigma$ where x is the channel ratio, µ is the mean channel ratio of the metabolite across all replicates, and σ is the standard deviation of the channel ratio of the metabolite across all replicates. Hierarchical clustering was performed using ComplexHeatmap[27] library version 2.10.0 for R version 4.1.1. The distance matrix was calculated using the “euclidean” method and clustering was performed using the “complete” method. Metabolite class annotations were curated through literature review.

bioRxiv preprint doi: https://doi.org/10.64898/2026.03.04.709614; this version posted March 6, 2026. The copyright holder for this preprint (which was not certified by peer review) is the author/funder, who has granted bioRxiv a license to display the preprint in perpetuity. It is made available under aCC-BY 4.0 International license.

Isotope tracing experiments

Sample preparation

T cells that had either been activated for 2 days (D2) or were cultured with and without chronic stimulation until day 8 post-activation as described in "Generation of acutely and chronically stimulated primary human T cells" (D8A, D8C) were washed with PBS and re-suspended in RPMI-1640 without glucose or glutamine (Media Preparation Core, MSKCC) containing 10% dialyzed FBS (GeminiBio, 100-108), 100 U/mL Penicillin-Streptomycin (ThermoFisher, 15140163), 5 μM BME (ThermoFisher, 21985023), 5 ng/mL hIL-7 (Peprotech, 200-07), and 5 ng/mL hIL-15 (Peprotech, 200-15), to which either universal ^{12}C-glucose (Millipore Sigma, G7021) or [U-^{13}C] glucose (Cambridge Isotope Laboratories, CLM-1396-PK) to a final concentration of 10 mM and either ^{12}C-L-glutamine (ThermoFisher, A2916801) or [U-^{13}C] glutamine (Cambridge Isotope Laboratories, CLM-1822-H-PK) to a final concentration of 2 mM were added. The cells were plated at 1.5 x 10^6 cell/mL in 1 mL of the respective medium and incubated at 37 °C for 4 h. Following this time, plates were placed on ice, cells were rapidly harvested, centrifuged at 300 x *g* for 3 min at 4 °C, and resuspended in 1 mL of ice-cold 80% LC-MS grade methanol (Fisher scientific, A456-4) for metabolite extraction, which was carried out at -80 °C overnight. Subsequently, samples were vortexed and centrifuged at 20,000 x *g* for 20 min to remove proteins. Supernatants were then dried in a vacuum evaporator (Genevac EZ-2 Elite) for 3 h.

Isotopologue analysis

Dried extracts were resuspended in 60 μL of 60% acetonitrile in water for hydrophilic interaction liquid chromatography (HILIC) by the Cell Metabolism Core within the Donald B. and Catherine C. Marron Cancer Metabolism Center at MSKCC. Samples were vortexed, incubated on ice for 20 min and clarified by centrifugation at 20,000 x g for 20 min at 4 °C. HILIC LC-MS analysis was performed on a 6545 Q-TOF mass spectrometer (Agilent Technologies) in both positive and negative ionization modes using columns, buffers and LC-MS parameters as described previously[3]. Targeted data analysis, isotopologue extraction, and natural isotope abundance correction were performed using MassHunter Profinder software v.10.0 (Agilent Technologies).

Proteomic platforms: Abundance-based proteomics with TMT multiplexing

Sample preparation

Six 10-plex experiments with primary human T cells from different biological donors were processed. Five experimental conditions were used in duplicate for each donor, including D2, D4A, D4C, D8A, and D8C T cells. Cells were pelleted (600 x *g*, 5 min, 4 °C), washed once with PBS, transferred into 1.5 mL Eppendorf tubes, flash-frozen, and stored at -80 °C until further processing. On the first day of the mass spectrometry workflow, frozen cell pellets were thawed on ice for 10 min. Ice-cold PBS supplemented with cOmplete EDTA-free Protease Inhibitor Cocktail (1 tablet per 10 mL) was added, and cells were lysed by sonication (three cycles of 8 pulses at 60% output). Protein concentrations were then determined using the standard DC Protein Assay (Bio-Rad) and normalized to a final concentration of 1-2 mg/mL.

Abundance-based proteomics with TMT multiplexing was performed following previously published protocols[28]. Briefly, normalized proteome samples (100 μL) were transferred into new 1.5 mL low-binding Eppendorf tubes containing 48 mg of urea, and vortexed to fully dissolve the urea, resulting in a final concentration of 8 M. DTT was then added (5 μL of 200 mM stock; 10 mM final concentration), and the samples were incubated at 65 °C for 15 min. After cooling for 5 min at room temperature, iodoacetamide was added (5 μL of 400 mM stock; 20 mM final concentration), followed by incubation at 37 °C for 30 min. The tubes were then placed on ice, and 400 μL of water was added to each sample. Proteins were precipitated using a methanol/chloroform protocol: 500 μL of cold methanol and 150 μL of chloroform were added to each tube, followed by centrifugation at 10,000 x *g* for 10 min to pellet the proteins. The supernatant was carefully removed without disturbing the protein pellet. If the protein pellet appeared small, the lower chloroform layer could be left in place until after the second methanol wash. An additional 400 μL of cold methanol was added to each sample, and the tubes were briefly sonicated to break up the protein disks. Samples were centrifuged again (10,000 x *g*, 10 min), and the resulting pellets were re-suspended with sonication in 160 μL of 200 mM EPPS buffer (pH 8.0, no urea). Finally,

bioRxiv preprint doi: https://doi.org/10.64898/2026.03.04.709614; this version posted March 6, 2026. The copyright holder for this preprint (which was not certified by peer review) is the author/funder, who has granted bioRxiv a license to display the preprint in perpetuity. It is made available under aCC-BY 4.0 International license.

6 µL of Trypsin/LysC solution (0.42 µg/µL in buffer containing 15 mM $CaCl_2$) was added to each sample. Digestion was carried out overnight at 37 °C with shaking.

Following the overnight digestion, peptide concentration was determined using the microBCA assay (Thermo Fisher) following manufacturer's instructions. For each TMT labeling reaction, 25 µg of peptides was used, and the volume was adjusted to 35 µL with 200 mM EPPS buffer. Acetonitrile was then added (9 µL per sample), followed by the addition of the appropriate TMT tag (5 µL per sample; 20 µg/µL in anhydrous acetonitrile), resulting in a final acetonitrile concentration of approximately 25%. Samples were incubated at room temperature for 1 hour to allow labeling. Unreacted tags were quenched by adding 5 µL of 5% hydroxylamine in water and incubating for 15 min. Finally, the samples were acidified with 2.5 µL of formic acid per sample.

A ratio check (RC) sample was prepared by pooling 2 µL from each labeled sample. The combined sample was dried using a SpeedVac vacuum concentrator and desalted using C18 stage tips (10 µL, Thermo Fisher). Briefly, stage tips were activated and equilibrated with two washes of 20 µL acetonitrile, followed by three washes with 20 µL of buffer A (0.1% formic acid, 5% acetonitrile, 95% water). The dried peptide mixture was reconstituted in 20 µL of buffer A with sonication, then loaded onto the stage tip by centrifugation (1,000 x *g*, 1 min). After washing with buffer A (3 × 20 µL, 1,000 x *g*, 1 min), peptides were eluted with 20 µL of buffer B (0.1% formic acid, 20% acetonitrile, 80% water) and dried again using a SpeedVac.

The RC sample was reconstituted in 10 µL of buffer A and analyzed via liquid chromatography-mass spectrometry (LC-MS) using a 2 µL injection on an Orbitrap Eclipse mass spectrometer. The following 70-min LC gradient was used: 0-10 min: 5% buffer B (0.1% FA in acetonitrile) in buffer A (0.1% FA in water); 10-50 min: linear increase from 5% to 20% buffer B; 50-55 min: ramp to 45% buffer B; 55-57 min: ramp to 95% buffer B; 57-59 min: hold at 95% buffer B; 59-61 min: decrease to 5% buffer B; 61-63 min: ramp back to 95% buffer B; 63-70 min: re-equilibration at 5% buffer B. The RC sample was used to calculate normalization factors for each TMT channel. For a 10-plex experiment, a volume equivalent to N × 20 µL (where N is the normalization factor derived from RC) was taken from each channel and combined into a single tube. The pooled sample was dried overnight using a SpeedVac and desalted using a SepPak C18 column (50 mg). The column was first activated with 2 × 1 mL of acetonitrile, then equilibrated with 3 × 1 mL of buffer A. The dried peptide mixture was reconstituted in 1 mL of buffer A, loaded onto the column, washed with buffer A, and eluted with buffer B into a clean Eppendorf tube. The eluate was dried overnight in a SpeedVac and subjected to high-pH reversed-phase fractionation using an HPLC system.

High pH HPLC fractionation (general protocol for all LC-MS/MS/MS workflows)

High pH HPLC fractionation was performed following previously published protocols[28]. The desalted peptide sample was reconstituted in 500 µL of Buffer A and subjected to high-pH reversed-phase fractionation using HPLC (Agilent) into a 96-well deep-well plate. Separation was performed on a Zorbax Extend-C18 column (3.5 µm, 4.6 × 250 mm) at a flow rate of 0.5 mL/min. The following gradient was used: 0-2 min: 100% Buffer C; 2-3 min: 0% to 13% Buffer D; 3-60 min: 13% to 50% Buffer D; 60-70 min: 50% to 80% Buffer D; 71-75 min: 100% Buffer D; 75-76 min: 100% to 0% Buffer D; 76-85 min: 100% Buffer C; 85-88 min: 0% to 13% Buffer D; 88-90 min: 13% to 80% Buffer D; 90-95 min: hold at 80% Buffer D; 96-101 min: 100% Buffer C; 101-104 min: 0% to 13% Buffer D; 104-106 min: 13% to 80% Buffer D; 106-111 min: hold at 80% Buffer D; 111-112 min: 80% to 0% Buffer D. The following buffers were used: buffer C - 10 mM ammonium bicarbonate (aqueous); buffer D - acetonitrile. To ensure acidification of eluting peptides, each well of the receiving plate was pre-loaded with 10 µL of 20% formic acid. Fractions were collected in a time-dependent mode across the plate in the following order: A1-A12, B1-B12, C1-C12, D1-D12, E1-E12, F1-F12, G1-G12, and H1-H12. After fractionation, samples were dried overnight using a SpeedVac vacuum concentrator. Wells from each column (e.g., A1–H1) were then combined to generate a total of 12 final fractions. These pooled samples were again dried overnight, reconstituted in 10 µL of Buffer A, and prepared for LC-MS/MS/MS analysis.

General protocol for liquid chromatography mass spectrometry analysis of TMT multiplexed samples

Peptide samples were analyzed by liquid chromatography-tandem mass spectrometry (LC-MS/MS/MS) using a standard 160-min gradient. The scan sequence consisted of the following steps: (1) MS1 was

bioRxiv preprint doi: https://doi.org/10.64898/2026.03.04.709614; this version posted March 6, 2026. The copyright holder for this preprint (which was not certified by peer review) is the author/funder, who has granted bioRxiv a license to display the preprint in perpetuity. It is made available under aCC-BY 4.0 International license.

acquired in the Orbitrap at a resolution of 120,000 over a mass range of 400–1600 m/z, with a 40% RF lens setting. The automatic gain control (AGC) target was set to 250% (normalized), and the maximum injection time was set to automatic. Dynamic exclusion was enabled with a repeat count of 1 and an exclusion duration of 60 s. Data were acquired in profile mode. (2) The top 10 precursor ions from the MS1 scan were selected for MS2/MS3 analysis. Precursor ions were isolated using the quadrupole with a 0.7 m/z isolation window, followed by collision-induced dissociation (CID) in the ion trap. The following parameters were used: standard AGC, 35% normalized collision energy (NCE), and a maximum injection time of 120 ms. (3) Real-time search (RTS) and synchronous precursor selection (SPS) were enabled to select up to 20 MS2 fragment ions for MS3 analysis. MS3 spectra were acquired in the Orbitrap following high-energy collision-induced dissociation (HCD) with 55% NCE, a normalized AGC target of 500%, a maximum injection time of 118 ms, and a resolution of 60,000. The isolation window for MS3 was set at 0.7 m/z.

Raw data files were processed using RAW Converter (v1.1.0.22; available at github.com/proteomicsyates/RawConverter), and the resulting MS1, MS2, and MS3 files were uploaded to the Integrated Proteomics Pipeline (IP2). Peptide identification was performed using the ProLuCID algorithm against a reverse concatenated, non-redundant version of the Human UniProt database (release 2024_03). Cysteine residues were searched with a static modification for carbamidomethylation (+57.02146 Da). A differential modification for iodoacetamide desthiobiotin labeling (+398.25292 Da) was included for site-of-labeling experiments. TMT labeling was considered as a static modification on peptide N-termini and lysine residues (+229.1629 Da for TMT 10-plex, +304.2071 Da for TMT 16-plex). Search results were filtered using DTASelect (v2.0) to maintain a peptide-level false discovery rate (FDR) below 1%. Quantification was performed using MS3 reporter ions with a mass tolerance of 20 ppm via the IP2 platform.

Data processing and analysis for TMT-exp experiments

Data processing

Six 10-plex experiments with primary human T cells from different biological donors were processed. Proteins were required to have at least two unique quantified peptides to pass into the final list. Peptide ratios (signal intensity/sum of signal intensities per peptide) were calculated and ratios of all peptides per protein were averaged. Keratins were not included in analysis. For experiments using the standard protocol, proteins needed to be quantified in at least 2 experiments to be included in the final table. To account for potential variation in expression between donors, proteins detected in two replicates were excluded from analysis if the ratio of the two replicates was > 2 and one replicate had an absolute fold-change < 1.5. For experiments using the low-input protocol, a curated list of proteins (PDCD1, LAG3, TIGIT, HAVCR2 , CD39, NR4A1, NR4A3, GZMB, GSR, CD62L, CTLA4, PRKCH, PRKCQ, and SAMHD1) were included in the final table if they were quantified in a single experiment. For the experiment containing human TILs, the requirement that proteins be quantified in two replicates was not applied. For experiments containing *in vivo* samples, hemoglobins were excluded. For experiments using the low-input protocol, normalization factors were calculated by dividing the global median (the median of all channel medians) by each individual channel's median. Each channel was then multiplied by its corresponding normalization factor.

Principal Component Analysis

Principal component analysis was carried out using *scikit-learn*[24] library in Python. Protein ratio values were $\log_2$-transformed, and only proteins quantified across all experiments were included. The first two principal components (PC1 and PC2) were plotted. Loadings for plotting were selected by taking 5 proteins with highest and lowest loadings for both principal components.

Gene set enrichment analysis of PCA loadings

PCA loadings were used for GSEA using the prerank function in GSEAPy v.1.1.4 with the gene ontology biological process database ("c5.go.bp") set as the database. The top 15 enriched terms based on NES were plotted.

bioRxiv preprint doi: https://doi.org/10.64898/2026.03.04.709614; this version posted March 6, 2026. The copyright holder for this preprint (which was not certified by peer review) is the author/funder, who has granted bioRxiv a license to display the preprint in perpetuity. It is made available under aCC-BY 4.0 International license.

Proteomic platforms: Cysteine reactivity profiling, TMT-ABPP

Sample preparation

Five 10-plex experiments with primary human T cells from different biological donors were processed. Five experimental conditions were used in duplicate for each donor, including D2, D4A, D4C, D8A, and D8C T cells. Cells were pelleted (600 x *g*, 5 min, 4 °C), washed once with PBS, transferred into 1.5 mL Eppendorf tubes, flash-frozen, and stored at -80 °C until further processing. On the first day of the mass spectrometry workflow, frozen cell pellets were thawed on ice for 10 min. Ice-cold PBS (560 µL/sample) was added and cells were lysed by sonication (three cycles of 8 pulses at 60% output). Protein concentrations were then determined using the standard DC Protein Assay (Bio-Rad) and normalized to a final concentration of 1-2 mg/mL.

Cysteine reactivity profiling experiments with TMT multiplexing were performed following previously published protocols[28]. Briefly, normalized proteome samples (500 µL) were transferred into new 1.5 mL low-binding Eppendorf tubes and iodoacetamide-PEG-desthiobiotin was added (5 µL of 10 mM stock; 100 µM final concentration), and the samples were incubated at room temperature for 1 hour. The tubes were then placed on ice, and proteins were precipitated using a methanol/chloroform protocol described above. The resulting pellets can be stored at -80 °C overnight. The pellets were then re-suspended with sonication in 90 µL of 8M urea TEAB buffer with DTT (2.2 g urea, 6.2 mg DTT, 2 mL H_2O, and 200 µL 1 M TEAB buffer, pH 8.5), and incubated at 65 °C for 20 min. After cooling for 5 min at room temperature, iodoacetamide was added (10 µL of 500 mM stock; 50 mM final concentration), followed by incubation at 37 °C for 30 min.

Trypsin/LysC digestion and streptavidin enrichment

To reduce the urea concentration, samples were diluted with 300 µL of TEAB buffer (50 mM, pH 8.5). Next, 4 µL of Trypsin/LysC (0.5 µg/µL in the appropriate buffer) and 4 µL of 100 mM $CaCl_2$ (final concentration 1 mM) were added, and the digestion was carried out overnight at 37 °C with shaking. The following day, 25 µL of compact, pre-washed streptavidin-agarose beads resuspended in 300 µL of enrichment buffer (50 mM TEAB, pH 8.5, with 150 mM NaCl and 0.2% NP40) were added to each sample, and the mixture was rotated for 3 h at room temperature. After the enrichment step, the beads were pelleted by centrifugation (2000 × *g*, 1 min), transferred to BioSpin columns, and washed extensively (three washes each with 1 mL of wash buffer, 1 mL of PBS, and 1 mL of water). Finally, peptides were eluted into a new Eppendorf tube with a 50% acetonitrile:water solution containing 0.1% formic acid, and the solvent was removed using a SpeedVac vacuum concentrator.

TMT labeling

The resulting protein digests were reconstituted in 100 µL of 30% acetonitrile in water containing 0.1% formic acid, with sonication to ensure complete dissolution, and used for TMT labeling. For each sample ("channel"), 3 µL of TMT reagent (20 µg/µL in dry acetonitrile) was added, followed by vortexing and brief centrifugation. Samples were then incubated for 1 h at room temperature. To quench unreacted TMT tags, 3 µL of 5% hydroxylamine in water was added, followed by vortexing and a 15-min incubation. The samples were acidified with 5 µL of formic acid per sample and pooled into a single Eppendorf tube. Solvent was removed overnight using a SpeedVac vacuum concentrator. The combined sample was then desalted using a SepPak C18 column (50 mg), as previously described. After another round of solvent removal via SpeedVac, the sample underwent high-pH fractionation using HPLC, following the same protocol as outlined above.

Data processing and analysis for TMT-ABPP

Five 10-plex TMT experiments with primary human T cells from different biological donors were analyzed. The MS3-based peptide quantification was conducted using the Integrated Proteomics Pipeline (IP2), with the reporter ion mass tolerance set to 20 ppm. To ensure data quality at the level of individual TMT experiments, several filtering steps were applied: non-unique peptides, half-tryptic peptides, peptides with more than one internal missed cleavage, and peptides with low total reporter ion intensity (<10,000 across five channels per donor) were excluded. Proteins were required to have at least one unique quantified peptide in each experiment. Peptide channel ratios were calculated (peptide total intensity of

bioRxiv preprint doi: https://doi.org/10.64898/2026.03.04.709614; this version posted March 6, 2026. The copyright holder for this preprint (which was not certified by peer review) is the author/funder, who has granted bioRxiv a license to display the preprint in perpetuity. It is made available under aCC-BY 4.0 International license.

each channel was normalized by the sum of signal intensities for all peptides in the same channel) and cysteine aggregation was performed to aggregate signal intensities for multiple peptides for the same cysteine (e.g. missed cleavage sites, charge states). Experiments were combined, requiring all peptides to be quantified in minimum 2 experiments. Averaged values (median) were calculated and used to calculate the R ratios of treatment groups (D4A, D8A, D4C, D8C) to control (D2).

Reactivity change analysis

To control for potential donor-specific variability in protein expression, proteins were included in the analysis only if at least one peptide R ratio fell within a 1.5-fold range of the corresponding protein expression level observed in the TMT-exp experiments (when available). Proteins were excluded if all peptide R ratios exceeded a 2.0-fold increase or fell below a 0.5-fold decrease. Additionally, peptides detected in two replicates were excluded from analysis if the ratio of the two replicates was > 2 and one replicate had an absolute fold-change < 1.5.

For proteins with two or more quantified peptides, a cysteine was considered for potential reactivity changes if its peptide R ratio deviated by more than two-fold from the corresponding protein expression level determined by TMT-exp data. Additionally, the highest and lowest peptide R ratios within the protein had to differ by more than two-fold. All identified reactivity changes were subsequently reviewed and curated manually.

Proteomic platforms: ATP-addback, TMT-ABPP

Two 16-plex experiments with primary human T cells from different biological donors were processed. Six experimental conditions were used in duplicate or triplicate for each donor, including D2, D2 + ATP, D8A, D8A + ATP, D8C, D8C + ATP. T cells were pelleted (600 x *g*, 5 min, 4 °C), washed once with PBS, transferred into 1.5 mL Eppendorf tubes, flash-frozen, and stored at -80 °C until further processing. On the first day of the mass spectrometry workflow, frozen cell pellets were thawed on ice for 10 min. Ice-cold PBS containing 1 mM $MgCl_2$ and protease inhibitor cocktail (560 µL/sample) was added and cells were lysed by sonication (three cycles of 8 pulses at 60% output). Protein concentrations were then determined using the standard DC Protein Assay (Bio-Rad), normalized to a final concentration of 1-2 mg/mL, and transferred into new 1.5 mL low-binding Eppendorf tubes (500 µL/channel). ATP stock solution (500 mM) was prepared by dissolving powder in molecular biology grade water and adjusting pH to 7.4 with pH strips. ATP (5 µL, 500 mM) was added to ATP-addback groups, molecular biology grade water (5 µL) was added to control groups, and the mixture was incubated at ambient temperature for 10 min. After this pre-incubation step, samples were processed according to cysteine reactivity profiling workflow described above (starting with the iodoacetamide-PEG-desthiobiotin treatment step).

Proteomic platforms: Low-input abundance-based proteomics with TMT multiplexing

Cells were pelleted (600 x *g*, 5 min, 4 °C), washed once with PBS, transferred into 1.5 mL Eppendorf tubes, flash-frozen, and stored at -80 °C until further processing. Low-input abundance-based proteomics with TMT multiplexing was performed following a previously published protocol with slight modifications[29,30].

Trypsin/LysC digestion

On the first day of the mass spectrometry workflow, frozen cell pellets were thawed on ice for 5 min. Ice-cold PBS supplemented with cOmplete EDTA-free Protease Inhibitor Cocktail (1 tablet per 10 mL) was added, and cells were lysed by sonication (three cycles of 8 pulses at 60% output). Protein concentrations were then determined using the standard DC Protein Assay (Bio-Rad) and normalized to a final concentration of 0.5 mg/mL. Normalized proteome samples (20 µL) were transferred into PCR tubes. For each sample ("channel"), 3 µL of SP3 beads (1:1 mixture of hydrophobic and hydrophilic type in lysis buffer) and 30 µL of ethanol containing 20 mM DTT were added, followed by pipetting and brief centrifugation. After a 15 min incubation at room temperature, the PCR tubes were placed on a magnetic stand, and the liquid was aspirated. The beads were washed once with 100 µL of 80% ethanol, resuspended in 25 µL of PBS containing 20 mM iodoacetamide, incubated in the dark for 30 min at room temperature, and then 50 µL of ethanol containing 20 mM DTT was added. After another 15 min incubation at room temperature, the beads were washed twice with 80% ethanol. The remaining beads

bioRxiv preprint doi: https://doi.org/10.64898/2026.03.04.709614; this version posted March 6, 2026. The copyright holder for this preprint (which was not certified by peer review) is the author/funder, who has granted bioRxiv a license to display the preprint in perpetuity. It is made available under aCC-BY 4.0 International license.

were resuspended in 30 µL of 200 mM EPPS buffer (pH 8) containing 0.3 µg of Lys-C and 0.3 µg of trypsin and incubated at 37 °C overnight.

TMT labeling

The next day, 9 µL of acetonitrile and appropriate TMT tag (3 µL per sample; 20 µg/µL in anhydrous acetonitrile) were added to the mixture of digested peptides and beads, followed by gentle mixing and brief centrifugation. After a 60 min incubation at room temperature, unreacted tags were quenched by adding 7 µL of 5% hydroxylamine in water. All TMT-labeled samples were combined into a single Eppendorf tube, dried using a SpeedVac, and desalted using a 100-mg Sep-Pak column. After another round of solvent removal via SpeedVac, the sample underwent high-pH fractionation using HPLC, following the same protocol as outlined above.

Proteomic platforms: Low-input cysteine reactivity profiling, TMT-ABPP

Cells were pelleted (600 x *g*, 5 min, 4 °C), washed once with PBS, transferred into 1.5 mL Eppendorf tubes, flash-frozen, and stored at -80 °C until further processing. Low-input cysteine reactivity profiling with TMT multiplexing was performed following a previously published protocol with slight modifications[30].

IA-PEG-DTB labeling and Trypsin/LysC digestion

On the first day of the mass spectrometry workflow, frozen cell pellets were thawed on ice for 5 min. Ice-cold PBS supplemented with cOmplete EDTA-free Protease Inhibitor Cocktail (1 tablet per 10 mL) was added, and cells were lysed by sonication (three cycles of 8 pulses at 60% output). Protein concentrations were then determined using the standard DC Protein Assay (Bio-Rad) and normalized to a final concentration of 1-2 mg/mL. Normalized proteome samples (15 µL) were transferred into PCR tubes. For each sample ("channel"), iodoacetamide-PEG-desthiobiotin was added (5 µL of 2 mM stock; 500 µM final concentration), and the samples were incubated at room temperature for 1 h. After that, 3 µL of SP3 beads (1:1 mixture of hydrophobic and hydrophilic type in lysis buffer) and 30 µL of ethanol containing 20 mM DTT were added, followed by pipetting and brief centrifugation. After a 15 min incubation at room temperature, the PCR tubes were placed on a magnetic stand, and the liquid was aspirated. The beads were washed once with 100 µL of 80% ethanol, resuspended in 25 µL of PBS containing 20 mM iodoacetamide, incubated in the dark for 30 min at room temperature, and then 50 µL of ethanol containing 20 mM DTT was added. After another 15 min incubation, the beads were washed twice with 80% ethanol. The remaining beads were resuspended in 30 µL of 200 mM EPPS buffer (pH 8) containing 0.3 µg of Lys-C and 0.3 µg of trypsin and incubated at 37 °C overnight.

TMT labeling

The next day, 9 µL of acetonitrile and appropriate TMT tag (3 µL per sample; 20 µg/µL in anhydrous acetonitrile) were added to the mixture of digested peptides and beads, followed by gentle mixing and brief centrifugation. After a 60 min incubation at room temperature, unreacted tags were quenched by adding 7 µL of 5% hydroxylamine in water. All TMT-labeled samples were combined into a single Eppendorf tube, dried using a SpeedVac, and desalted using a 100-mg Sep-Pak column.

Streptavidin enrichment

The desalted TMT-labeled peptides were resuspended in 460 µL of 100 mM HEPES buffer (pH 7.4), 80 µL Pierce High Capacity Streptavidin Agarose (Thermo Fisher Scientific) were added and the mixture was incubated at room temperature for 3 h. The resulting mixture was then loaded on a Ultrafree-MC centrifugal filter (hydrophilic PTFE, 0.22 µm pore size) and centrifugated at 1,000 x *g* for 30 s. Beads were washed sequentially with 300 µL of 100 mM HEPES (pH 7.4) with 0.05% NP-40 twice, 350 µL of 100 mM HEPES (pH 7.4) three times, and 400 µL of H_2O once. Peptides were eluted sequentially by 1) elution buffer (80% acetonitrile, 0.1% formic acid) with 20 min incubation at room temperature, 2) elution buffer with 20 min incubation at room temperature; 3) elution buffer with 10 min incubation at 72 °C. The combined eluent was dried in a SpeedVac.

bioRxiv preprint doi: https://doi.org/10.64898/2026.03.04.709614; this version posted March 6, 2026. The copyright holder for this preprint (which was not certified by peer review) is the author/funder, who has granted bioRxiv a license to display the preprint in perpetuity. It is made available under aCC-BY 4.0 International license.

High pH fractionation

The desalted peptide sample was reconstituted in 300 µL of 0.1% TFA solution and subjected to high-pH reversed-phase fractionation using a fractionation kit (Thermo Fisher Scientific) into Eppendorf tubes. The sample was fractionated into 8 fractions, dried using a SpeedVac, reconstituted in 10 µL of 5% formic acid/5% acetonitrile solution, and prepared for LC-MS/MS/MS analysis.

Western blot analysis

T cells were pelleted (600 x *g*, 5 min, 4 °C), washed with PBS, transferred to 1.5 mL Eppendorf tubes, flash-frozen, and stored at -80 °C until further analysis. On the day of the analysis, frozen cell pellets were thawed on ice, resuspended in ice-cold PBS containing protease inhibitor cocktail (Roche) and phosphatase inhibitor cocktail (Roche) and lysed by sonication (3 x 8 pulses, 60% duty cycle). Protein concentrations were adjusted to 1-2 mg/mL, 4x Laemmli sample buffer was added, and the samples were heated at 95 °C for 7 min. Proteins were resolved using SDS-PAGE (4–20% Criterion TGX Stain-Free Protein Gel, BioRad) and transferred to 0.45 µm polyvinylidene fluoride membranes (Cytiva). For total protein staining, the membrane was fully dried at room temperature for 1 h and rehydrated using MeOH. Following 30 s incubation, the membrane was washed with PBS and H_2O, and then stained with Revert 700 (LICORbio) at room temperature for 5 min. The membrane was rinsed 2 times with Revert 700 wash solution (LICORbio) and the images were captured using the 680 nm channel on a ChemiDoc MP Imaging System (Bio-Rad). After capturing the images of total protein staining, the membranes were blocked with 5% milk or 5% BSA in Tris-buffered saline (20 mM Tris-HCl pH 7.6, 150 mM NaCl) with 0.1% tween 20 (TBS-T) buffer at room temperature for 30 min, washed 3 times with TBS-T, and incubated with primary antibodies in 5% BSA in TBS-T at 4 °C overnight. The membranes were washed 3 times with TBS-T, incubated with HRP-conjugated secondary antibodies in 5% BSA in TBS-T at room temperature for 1 h and washed 5 times with TBS-T. The immunoreactive bands were detected using Pierce ECL Western Blotting Substrate (Thermo Fisher Scientific) on the ChemiDoc MP Imaging System (Bio-Rad). Blots were quantified with densiometric analysis using ImageJ software (NIH) and normalized to the total amount of protein in each lane.

Subcellular fractionation

Activated (D2), acutely (D8A), and chronically stimulated (D8C) T cells were pelleted (600 x *g*, 5 min, 4 °C), washed with PBS, transferred to low binding 1.5 mL Eppendorf tubes, flash-frozen, and stored at -80 °C until further analysis. On the day of the analysis, frozen cell pellets were thawed on ice, resuspended in ice-cold fractionation buffer (20 mM HEPES [pH 7.4], 250 mM sucrose, 10 mM KCl, 2 mM $MgCl_2$, 1 mM EGTA, 1 mM EDTA, 1 mM DTT, protease inhibitor cocktail), homogenized by passing cell suspension through a 25 gauge needle 20 times and incubated on ice for 20 min. All samples were kept at 4 °C for the duration of the fractionation. The nuclei were pelleted by centrifugation (720 x *g*, 5 min, 4 °C) and the supernatant, which includes the mitochondria, membrane and cytoplasm, was saved for further processing. The nuclear pellet was washed and resuspended in 500 µL of fractionation buffer, passed through a 25 gauge needle 20 times, and pelleted (720 x *g*, 10 min, 4 °C). The wash step was repeated one more time. The nuclear pellet was then resuspended in TBS with 0.1% SDS with sonication (2 x 8 pulses, 60% duty cycle) to shear genomic DNA and homogenize the suspension. The supernatant containing the mitochondria, membrane and cytoplasm was centrifuged (10,000 x *g*, 5 min, 4 °C) to pellet the mitochondrial fraction. The supernatant was transferred to a new Eppendorf tube. The mitochondrial pellet was then washed 3 times in fractionation buffer and resuspended in 200 µL of TBS with 0.1% SDS with sonication (2 x 8 pulses, 60% duty cycle). The supernatant containing the membrane and cytoplasm was ultracentrifuged (100,000 x *g*, 1 h, 4 °C). The supernatant was re-centrifuged (100,000 x *g*, 45 min, 4 °C) and saved as cytoplasm fraction. The membrane pellet was washed, resuspended in 400 µL of fractionation buffer, passed through a 25 gauge, and re-centrifuged (100,000 x *g*, 45 min, 4 °C). The membrane pellet was then resuspended in 500 µL of TBS with 0.1% SDS with sonication (2 x 8 pulses, 60% duty cycle). Protein concentration was adjusted to 2.0 mg/mL and the samples were mixed with 4x Laemmli sample buffer, boiled at 95 °C for 7 min, resolved by SDS-PAGE and immunoblotted.

bioRxiv preprint doi: https://doi.org/10.64898/2026.03.04.709614; this version posted March 6, 2026. The copyright holder for this preprint (which was not certified by peer review) is the author/funder, who has granted bioRxiv a license to display the preprint in perpetuity. It is made available under aCC-BY 4.0 International license.

Fractionation of Triton X-100 soluble and insoluble proteins

T cells were pelleted (600 x *g*, 5 min, 4 °C), washed with PBS, transferred to low binding 1.5 mL Eppendorf tubes, flash-frozen, and stored at -80 °C until further analysis. On the day of the analysis, cell pellets were thawed on ice, resuspended in 150 µL ice-cold fractionation buffer (20 mM Tris-HCl, pH 7.4, 150 mM NaCl, 2 mM EDTA, 1% Triton X-100, protease inhibitor cocktail), and lysed by pipetting the solution up and down. Protein concentrations were measured using a standard DC assay (Bio-Rad) and normalized to 1-2 mg/mL. Normalized cell lysates were transferred to new low binding Eppendorf tubes (300 µL/tube) and centrifuged at 17,000 x *g* for 10 min at 4 °C to separate into supernatant and pellet fractions. The supernatant was saved as soluble fraction and the pellet was washed with ice-cold fractionation buffer. The supernatant and insoluble pellet were reconstituted in equivalent volumes of Laemmli sample buffer, boiled at 95 °C for 7 min and analyzed by Western blotting.

Western blot based cysteine reactivity profiling

Frozen cell pellets were thawed on ice for 5 minutes. Cells were resuspended in PBS containing 1 mM $MgCl_2$ and cOmplete EDTA-free Protease Inhibitor Cocktail (1 tablet per 10 mL), and lysed by sonication (three cycles of 8 pulses at 60% output). Protein concentrations were then determined using the standard DC Protein Assay (Bio-Rad), normalized to a final concentration of 1 mg/mL, and transferred into low binding 1.5 mL Eppendorf tubes (46 µL/condition). ATP stock solution (125 mM) was prepared by dissolving powder in molecular biology grade water and adjusting pH to 7.4 with pH strips. ATP (2 µL of 125 mM stock; 5 mM final concentration) was added to ATP-addback groups, molecular biology grade water (1 µL) was added to control groups, and the mixture was incubated at room temperature for 10 min. After the pre-incubation step, a cysteine reactive probe $MM(PEG)_{24}$ (2 µL of 250 mM stock; 10 mM final concentration) was added to samples, and the mixture was incubated at room temperature for 30 min. The reaction was quenched with 16.6 µL 4x sample buffer and boiled at 95 °C for 7 min. Samples were then analyzed by Western blot.

Proteasome activity assay

Frozen cell pellets were thawed on ice for 5 min. Cells were resuspended in PBS containing 1 mM $MgCl_2$ and cOmplete EDTA-free Protease Inhibitor Cocktail (1 tablet per 10 mL), and lysed by sonication (three cycles of 8 pulses at 60% output). Protein concentrations were then determined using the standard DC Protein Assay (Bio-Rad), normalized to a final concentration of 1.14 mg/mL, and transferred into PCR tubes (22 µL/condition). MG132 (1 µL of 625 µM stock; 25 µM final concentration) was added to MG132 groups, DMSO (1 µL) was added to control groups, and the mixture was incubated at room temperature for 1 h. ATP stock solutions (2.5, 25, 125 mM) were prepared by dissolving powder in molecular biology grade water and adjusting pH to 7.4 with pH strips. Following the MG132 treatment, ATP (1 µL; 0.1, 1, 5 mM final concentrations) was added to ATP-addback groups, molecular biology grade water (1 µL) was added to control groups, and the mixture was incubated at room temperature for 10 min. After these pre-incubation steps, a pan-reactive fluorescent proteasome activity-based probe Me4BodipyFL-Ahx3Leu3VS (1 µL of 25 µM stock; 1 µM final concentration) was added to samples, and the mixture was incubated at room temperature for 1 h. The reaction was quenched with 8.3 µL 4x sample buffer and boiled at 95 °C for 7 min. Samples were then resolved on SDS-PAGE (4–20% Criterion TGX Stain-Free Protein Gel, BioRad) and analyzed using the appropriate fluorescence channel on a ChemiDoc MP Imaging System (Bio-Rad).

Inhibitor treatments

BSJ-04-122 (Millipore Sigma) was used to inhibit MAP2K4/7[31] and was diluted from a 10 mM stock in DMSO to a final concentration of 10 µM. ISRIB (MedChemExpress) was used to inhibit integrated stress response[32] and was diluted from a 200 µM stock in DMSO to a final concentration of 200 nM. Thapsigargin (Millipore Sigma) was used to induce ER stress[33] and was diluted from a 25 µM stock in DMSO to a final concentration of 25 nM. T cells were cultured with BSJ-04-122 or ISRIB from day 2 to day 8, or Thapsigargin from day 7 to day 8.

bioRxiv preprint doi: https://doi.org/10.64898/2026.03.04.709614; this version posted March 6, 2026. The copyright holder for this preprint (which was not certified by peer review) is the author/funder, who has granted bioRxiv a license to display the preprint in perpetuity. It is made available under aCC-BY 4.0 International license.

Statistical Analysis

Statistical analysis of flow cytometry, Western blot, and functional assays was performed using GraphPad Prism 10.3.0. Samples derived from a given blood donor were treated as paired for comparisons by one- and two-way repeated measures ANOVA. Where relevant, Gaussian distribution and sphericity were assumed, and multiple comparisons corrections included. Details of statistical tests are available in figure legends.

Generation of reference protein tables

Lists of reference proteins were created by retrieving data from the Gene Ontology (go-basic.obo, downloaded on June 20, 2023). Protein lists were created by identifying relevant Gene Ontology terms based on text searches:

Reference list name	GO terms used	GO term names
Nucleotide metabolism	GO:0008252, GO:0055086	nucleotidase activity, nucleobase-containing small molecule metabolic process
Cell cycle	GO:0007049, GO:0051301	cell cycle, cell division
Redox-related	GO:0045454, GO:0061692, GO:0098869, GO:0071500, GO:0034599, GO:0034614, GO:0034614, GO:0071461, GO:0042744, GO:0042743, GO:0072593, GO:0006979, GO:0000302, GO:0000302, GO:0051775	cell redox homeostasis, cellular detoxification of hydrogen peroxide, cellular oxidant detoxification, cellular response to nitrosative stress, cellular response to oxidative stress, cellular response to reactive oxygen species, cellular response to reactive oxygen species, cellular response to redox state, hydrogen peroxide catabolic process, hydrogen peroxide metabolic process, reactive oxygen species metabolic process, response to oxidative stress, response to reactive oxygen species, response to reactive oxygen species, response to redox state
Mitochondrial proteins	GO:0005739, GO:0031966, GO:0005759, GO:0098800, GO:0005758, GO:0044233, GO:0005740, GO:0042645, GO:0098799, GO:0005746	mitochondrion, mitochondrial membrane, mitochondrial matrix, inner mitochondrial membrane protein complex, mitochondrial intermembrane space, mitochondria-associated endoplasmic reticulum membrane, mitochondrial envelope, mitochondrial nucleoid, outer mitochondrial membrane protein complex, mitochondrial respirasome
Peroxisomal proteins	GO:0005777, GO:0005778, GO:0005782, GO:1990429	peroxisome, peroxisomal membrane, peroxisomal matrix, peroxisomal importomer complex
Nucleotide binding proteins	GO:0000166, GO:0005525, GO:0002135, GO:0005524	nucleotide binding, GTP binding, CTP binding, ATP binding
Respiratory complex I-IV	GO:0005747, GO:0005749, GO:0005750, GO:0005751	mitochondrial respiratory chain complex I, mitochondrial respiratory chain complex II, succinate dehydrogenase complex (ubiquinone), mitochondrial respiratory chain complex III, mitochondrial respiratory chain complex IV

bioRxiv preprint doi: https://doi.org/10.64898/2026.03.04.709614; this version posted March 6, 2026. The copyright holder for this preprint (which was not certified by peer review) is the author/funder, who has granted bioRxiv a license to display the preprint in perpetuity. It is made available under aCC-BY 4.0 International license.

Respiratory complex V	GO:0045259	proton-transporting ATP synthase complex

Generation of reference metabolite tables

Lists of reference metabolites were created by retrieval of reference metabolism pathways from the Kyoto Encyclopedia of Genes and Genomes (KEGG) followed by manual curation:

Reference list name	KEGG reference pathways
Glycolysis	Glycolysis / Gluconeogenesis
TCA	Citrate cycle (TCA cycle)
Pyrimidine nucleotide	Pyrimidine metabolism
Purine nucleotide	Purine metabolism

AlphaFold3 complex prediction of PUR1, AMP, and GMP.

Complex prediction of PUR1, AMP, and GMP was performed using AlphaFold3 (AF3, version 3.0.0) with all default parameters. The model parameters were obtained with permission from Google DeepMind. For multiple sequence alignments (MSAs), the Jackhammer module from HMMER (version 3.4) was employed. Sequence data for PUR1 were retrieved from UniProt (ID: Q06203) and co-folded with SMILES strings for AMP (C1=NC(=C2C(=N1)N(C=N2)[C@H]3[C@@H]([C@@H]([C@H](O3)COP(=O)(O)O)O)O)N) and GMP (C1=NC2=C(N1[C@H]3[C@@H]([C@@H]([C@H](O3)COP(=O)([O-])[O-])O)O)N=C(NC2=O)N). Five models each were generated from five random seeds (4, 8, 9, 20, 31), and the best-performing structure by AF3 ranking_score (0.76) was used for analysis and plotting in Figure S4E. These structures were conducted on the Rockefeller University high-performance computing cluster with NVIDIA L40 GPUs. The whole predictions take 40.2 minutes to run.

(E) References

1. Tooley, K., Jerby, L., Escobar, G., Krovi, S.H., Mangani, D., Dandekar, G., Cheng, H., Madi, A., Goldschmidt, E., Lambden, C., et al. (2024). Pan-cancer mapping of single CD8(+) T cell profiles reveals a TCF1:CXCR6 axis regulating CD28 co-stimulation and anti-tumor immunity. Cell Rep Med *5*, 101640. 10.1016/j.xcrm.2024.101640.
2. Chu, Y., Dai, E., Li, Y., Han, G., Pei, G., Ingram, D.R., Thakkar, K., Qin, J.J., Dang, M., Le, X., et al. (2023). Pan-cancer T cell atlas links a cellular stress response state to immunotherapy resistance. Nat Med *29*, 1550-1562. 10.1038/s41591-023-02371-y.
3. Vardhana, S.A., Hwee, M.A., Berisa, M., Wells, D.K., Yost, K.E., King, B., Smith, M., Herrera, P.S., Chang, H.Y., Satpathy, A.T., et al. (2020). Impaired mitochondrial oxidative phosphorylation limits the self-renewal of T cells exposed to persistent antigen. Nat Immunol *21*, 1022-1033. 10.1038/s41590-020-0725-2.
4. Matsumoto, T., Kinoshita, T., Kirii, Y., Yokota, K., Hamada, K., and Tada, T. (2010). Crystal structures of MKK4 kinase domain reveal that substrate peptide binds to an allosteric site and induces an auto-inhibition state. Biochem Biophys Res Commun *400*, 369-373. 10.1016/j.bbrc.2010.08.071.
5. Moseng, M.A., Nix, J.C., and Page, R.C. (2019). Biophysical Consequences of EVEN-PLUS Syndrome Mutations for the Function of Mortalin. J Phys Chem B *123*, 3383-3396. 10.1021/acs.jpcb.9b00071.
6. Shin, M., Watson, E.R., Song, A.S., Mindrebo, J.T., Novick, S.J., Griffin, P.R., Wiseman, R.L., and Lander, G.C. (2021). Structures of the human LONP1 protease reveal regulatory steps involved in protease activation. Nat Commun *12*, 3239. 10.1038/s41467-021-23495-0.
7. Guiley, K.Z., Stevenson, J.W., Lou, K., Barkovich, K.J., Kumarasamy, V., Wijeratne, T.U., Bunch, K.L., Tripathi, S., Knudsen, E.S., Witkiewicz, A.K., et al. (2019). p27 allosterically

bioRxiv preprint doi: https://doi.org/10.64898/2026.03.04.709614; this version posted March 6, 2026. The copyright holder for this preprint (which was not certified by peer review) is the author/funder, who has granted bioRxiv a license to display the preprint in perpetuity. It is made available under aCC-BY 4.0 International license.

activates cyclin-dependent kinase 4 and antagonizes palbociclib inhibition. Science *366*. 10.1126/science.aaw2106.

8. Shin, C.S., Meng, S., Garbis, S.D., Moradian, A., Taylor, R.W., Sweredoski, M.J., Lomenick, B., and Chan, D.C. (2021). LONP1 and mtHSP70 cooperate to promote mitochondrial protein folding. Nat Commun *12*, 265. 10.1038/s41467-020-20597-z.
9. Keough, D.T., Hockova, D., Holy, A., Naesens, L.M., Skinner-Adams, T.S., Jersey, J., and Guddat, L.W. (2009). Inhibition of hypoxanthine-guanine phosphoribosyltransferase by acyclic nucleoside phosphonates: a new class of antimalarial therapeutics. J Med Chem *52*, 4391-4399. 10.1021/jm900267n.
10. Yan, Q., Barros, T., Visperas, P.R., Deindl, S., Kadlecek, T.A., Weiss, A., and Kuriyan, J. (2013). Structural basis for activation of ZAP-70 by phosphorylation of the SH2-kinase linker. Mol Cell Biol *33*, 2188-2201. 10.1128/MCB.01637-12.
11. Wong, Y.L., LeBon, L., Basso, A.M., Kohlhaas, K.L., Nikkel, A.L., Robb, H.M., Donnelly-Roberts, D.L., Prakash, J., Swensen, A.M., Rubinstein, N.D., et al. (2019). eIF2B activator prevents neurological defects caused by a chronic integrated stress response. Elife *8*. 10.7554/eLife.42940.
12. Marton, M.J., Vazquez de Aldana, C.R., Qiu, H., Chakraburtty, K., and Hinnebusch, A.G. (1997). Evidence that GCN1 and GCN20, translational regulators of GCN4, function on elongating ribosomes in activation of eIF2alpha kinase GCN2. Mol Cell Biol *17*, 4474-4489. 10.1128/MCB.17.8.4474.
13. Sattlegger, E., and Hinnebusch, A.G. (2000). Separate domains in GCN1 for binding protein kinase GCN2 and ribosomes are required for GCN2 activation in amino acid-starved cells. EMBO J *19*, 6622-6633. 10.1093/emboj/19.23.6622.
14. Krishna, C., DiNatale, R.G., Kuo, F., Srivastava, R.M., Vuong, L., Chowell, D., Gupta, S., Vanderbilt, C., Purohit, T.A., Liu, M., et al. (2021). Single-cell sequencing links multiregional immune landscapes and tissue-resident T cells in ccRCC to tumor topology and therapy efficacy. Cancer Cell *39*, 662-677 e666. 10.1016/j.ccell.2021.03.007.
15. Li, J., Byrne, K.T., Yan, F., Yamazoe, T., Chen, Z., Baslan, T., Richman, L.P., Lin, J.H., Sun, Y.H., Rech, A.J., et al. (2018). Tumor Cell-Intrinsic Factors Underlie Heterogeneity of Immune Cell Infiltration and Response to Immunotherapy. Immunity *49*, 178-193 e177. 10.1016/j.immuni.2018.06.006.
16. Sanjana, N.E., Shalem, O., and Zhang, F. (2014). Improved vectors and genome-wide libraries for CRISPR screening. Nat Methods *11*, 783-784. 10.1038/nmeth.3047.
17. Kim, W.J., Crosse, E.I., De Neef, E., Etxeberria, I., Sabio, E.Y., Wang, E., Bewersdorf, J.P., Lin, K.T., Lu, S.X., Belleville, A., et al. (2025). Mis-splicing-derived neoantigens and cognate TCRs in splicing factor mutant leukemias. Cell *188*, 3422-3440 e3424. 10.1016/j.cell.2025.03.047.
18. Robbins, P.F., Li, Y.F., El-Gamil, M., Zhao, Y., Wargo, J.A., Zheng, Z., Xu, H., Morgan, R.A., Feldman, S.A., Johnson, L.A., et al. (2008). Single and dual amino acid substitutions in TCR CDRs can enhance antigen-specific T cell functions. J Immunol *180*, 6116-6131. 10.4049/jimmunol.180.9.6116.
19. Cohen, C.J., Zhao, Y., Zheng, Z., Rosenberg, S.A., and Morgan, R.A. (2006). Enhanced antitumor activity of murine-human hybrid T-cell receptor (TCR) in human lymphocytes is associated with improved pairing and TCR/CD3 stability. Cancer Res *66*, 8878-8886. 10.1158/0008-5472.CAN-06-1450.
20. Cohen, C.J., Li, Y.F., El-Gamil, M., Robbins, P.F., Rosenberg, S.A., and Morgan, R.A. (2007). Enhanced antitumor activity of T cells engineered to express T-cell receptors with a second disulfide bond. Cancer Res *67*, 3898-3903. 10.1158/0008-5472.CAN-06-3986.
21. Schmidt, R., Steinhart, Z., Layeghi, M., Freimer, J.W., Bueno, R., Nguyen, V.Q., Blaeschke, F., Ye, C.J., and Marson, A. (2022). CRISPR activation and interference screens decode stimulation responses in primary human T cells. Science *375*, eabj4008. 10.1126/science.abj4008.
22. Shifrut, E., Carnevale, J., Tobin, V., Roth, T.L., Woo, J.M., Bui, C.T., Li, P.J., Diolaiti, M.E., Ashworth, A., and Marson, A. (2018). Genome-wide CRISPR Screens in Primary Human T

bioRxiv preprint doi: https://doi.org/10.64898/2026.03.04.709614; this version posted March 6, 2026. The copyright holder for this preprint (which was not certified by peer review) is the author/funder, who has granted bioRxiv a license to display the preprint in perpetuity. It is made available under aCC-BY 4.0 International license.

Cells Reveal Key Regulators of Immune Function. Cell *175*, 1958-1971 e1915. 10.1016/j.cell.2018.10.024.
23. Rio, D.C., Ares, M., Jr., Hannon, G.J., and Nilsen, T.W. (2010). Purification of RNA using TRIzol (TRI reagent). Cold Spring Harb Protoc *2010*, pdb prot5439. 10.1101/pdb.prot5439.
24. Pedregosa, F.V., G.; Gramfort, A.; Michel, V.; Thirion, B.; Grisel, O.; Blondel, M.; Prettenhofer, P.; Weiss, R.; Dubourg, V.; Vanderplas, J.; Passos, A.; Cournapeau, D.; Brucher, M.; Perrot, M.; Duchesnay, E. (2011). Scikit-learn: Machine Learning in Python. Journal of Machine Learning Research. *12*, 2825-2830.
25. Satomi, Y., Hirayama, M., and Kobayashi, H. (2017). One-step lipid extraction for plasma lipidomics analysis by liquid chromatography mass spectrometry. J Chromatogr B Analyt Technol Biomed Life Sci *1063*, 93-100. 10.1016/j.jchromb.2017.08.020.
26. Tsugawa, H., Cajka, T., Kind, T., Ma, Y., Higgins, B., Ikeda, K., Kanazawa, M., VanderGheynst, J., Fiehn, O., and Arita, M. (2015). MS-DIAL: data-independent MS/MS deconvolution for comprehensive metabolome analysis. Nat Methods *12*, 523-526. 10.1038/nmeth.3393.
27. Gu, Z. (2022). Complex heatmap visualization. Imeta *1*, e43. 10.1002/imt2.43.
28. Scott, K.A., Kojima, H., Ropek, N., Warren, C.D., Zhang, T.L., Hogg, S.J., Sanford, H., Webster, C., Zhang, X., Rahman, J., et al. (2025). Covalent targeting of splicing in T cells. Cell Chem Biol *32*, 201-218 e217. 10.1016/j.chembiol.2024.10.010.
29. Hughes, C.S., Moggridge, S., Muller, T., Sorensen, P.H., Morin, G.B., and Krijgsveld, J. (2019). Single-pot, solid-phase-enhanced sample preparation for proteomics experiments. Nat Protoc *14*, 68-85. 10.1038/s41596-018-0082-x.
30. Yang, K., Whitehouse, R.L., Dawson, S.L., Zhang, L., Martin, J.G., Johnson, D.S., Paulo, J.A., Gygi, S.P., and Yu, Q. (2024). Accelerating multiplexed profiling of protein-ligand interactions: High-throughput plate-based reactive cysteine profiling with minimal input. Cell Chem Biol *31*, 565-576 e564. 10.1016/j.chembiol.2023.11.015.
31. Jiang, J., Jiang, B., He, Z., Ficarro, S.B., Che, J., Marto, J.A., Gao, Y., Zhang, T., and Gray, N.S. (2020). Discovery of Covalent MKK4/7 Dual Inhibitor. Cell Chem Biol *27*, 1553-1560 e1558. 10.1016/j.chembiol.2020.08.014.
32. Sidrauski, C., Acosta-Alvear, D., Khoutorsky, A., Vedantham, P., Hearn, B.R., Li, H., Gamache, K., Gallagher, C.M., Ang, K.K., Wilson, C., et al. (2013). Pharmacological brake-release of mRNA translation enhances cognitive memory. Elife *2*, e00498. 10.7554/eLife.00498.
33. Iurlaro, R., and Munoz-Pinedo, C. (2016). Cell death induced by endoplasmic reticulum stress. FEBS J *283*, 2640-2652. 10.1111/febs.13598.

bioRxiv preprint doi: https://doi.org/10.64898/2026.03.04.709614; this version posted March 6, 2026. The copyright holder for this preprint (which was not certified by peer review) is the author/funder, who has granted bioRxiv a license to display the preprint in perpetuity. It is made available under aCC-BY 4.0 International license.

KEY RESOURCES TABLE

REAGENT or RESOURCE	SOURCE	IDENTIFIER
**Western blot antibodies**		
Rabbit monoclonal anti-phospho-MAP2K3 (Ser189)/MAP2K6 (Ser207)	Cell Signaling Technology	Cat#: 9231; RRID: AB_2140799
Rabbit monoclonal anti-MAP2K3	Cell Signaling Technology	Cat#: 8535; RRID: AB_11220233
Rabbit monoclonal anti-phospho-SEK1/MAP2K4 (Ser257)	Cell Signaling Technology	Cat#: 4514; RRID: AB_2140946
Rabbit polyclonal anti-SEK1/MAP2K4	Cell Signaling Technology	Cat#: 9152; RRID: AB_330905
Rabbit monoclonal anti-phospho-p38 MAPK (Thr180/Tyr182)	Cell Signaling Technology	Cat#: 4511; RRID: AB_2139682
Mouse monoclonal anti-p38α MAPK	Cell Signaling Technology	Cat#: 9228; RRID: AB_490886
Rabbit monoclonal anti-phospho-SAPK/JNK (Thr183/Tyr185)	Cell Signaling Technology	Cat#: 4668; RRID: AB_823588
Rabbit polyclonal anti-SAPK/JNK	Cell Signaling Technology	Cat#: 9252; RRID: AB_2250373
Mouse monoclonal anti-GAPDH	Thermo Fisher Scientific	Cat#: MA1-16757; RRID: AB_568547
Rabbit monoclonal anti-Lamin B1	Cell Signaling Technology	Cat#: 12586; RRID: AB_2650517
Rabbit monoclonal anti-Citrate synthase	Cell Signaling Technology	Cat#: 14309; RRID: AB_2665545
Rabbit monoclonal anti-Sodium potassium ATPase	Thermo Fisher Scientific	Cat#: MA5-32184; RRID: AB_2809472
Rabbit monoclonal anti-Grp75/HSPA9	Cell Signaling Technology	Cat#: 3593; RRID: AB_2120328
Rabbit monoclonal anti-PRSS15/LONP1	Cell Signaling Technology	Cat#: 28020; RRID: AB_2798952
Mouse monoclonal anti-TID-1/DNAJA3	Santa Cruz Biotechnology	Cat#: sc-18819; RRID: AB_668803
Mouse monoclonal anti-NDUFA9	Invitrogen	Cat#: 459100; RRID: AB_10376187

bioRxiv preprint doi: https://doi.org/10.64898/2026.03.04.709614; this version posted March 6, 2026. The copyright holder for this preprint (which was not certified by peer review) is the author/funder, who has granted bioRxiv a license to display the preprint in perpetuity. It is made available under aCC-BY 4.0 International license.

REAGENT or RESOURCE	SOURCE	IDENTIFIER
Rabbit polyclonal anti-VDAC	Cell Signaling Technology	Cat#: 4866; RRID: AB_2272627
Rabbit monoclonal anti-ATF4	Cell Signaling Technology	Cat#: 11815; RRID: AB_2616025
Rabbit polyclonal anti-ATF5	Sigma-Aldrich	Cat#: HPA030187; RRID: AB_10601666
Mouse monoclonal anti-CHOP	Cell Signaling Technology	Cat#: 2895; RRID: AB_2089254
Rabbit polyclonal anti-HSF1	Cell Signaling Technology	Cat#: 4356; RRID: AB_2120258
Rabbit monoclonal anti-phospho-eIF2a (Ser51)	Cell Signaling Technology	Cat#: 3398; RRID: AB_2096481
Rabbit monoclonal anti-eIF2a	Cell Signaling Technology	Cat#: 5324; RRID: AB_10692650
Rabbit monoclonal anti-phospho-PERK (Thr982)	ABclonal	Cat#: AP1501; RRID: AB_2942102
Rabbit monoclonal anti-PERK	Cell Signaling Technology	Cat#: 3192; RRID: AB_2095847
Rabbit monoclonal anti-ATF6	Cell Signaling Technology	Cat#: 65880; RRID: AB_2799696
Rabbit polyclonal anti-ATF6	ABclonal	Cat#: A0202; RRID: AB_2757016
Rabbit monoclonal anti-IRE1α	Cell Signaling Technology	Cat#: 3294; RRID: AB_823545
Rabbit monoclonal anti-XBP-1s	Cell Signaling Technology	Cat#: 40435; RRID: AB_2891025
Rabbit monoclonal anti-BiP	Cell Signaling Technology	Cat#: 3177; RRID: AB_2119845
Rabbit monoclonal anti-phospho-Histone H2A.X (Ser139)	Cell Signaling Technology	Cat#: 9718; RRID: AB_2118009
Mouse monoclonal anti-phospho-ATM (Ser1981)	Cell Signaling Technology	Cat#: 4526; RRID: AB_2062663
Mouse monoclonal anti-ATM	Sigma-Aldrich	Cat#: A1106; RRID: AB_796190
Rabbit polyclonal anti-phospho-ATR (Thr1989)	GeneTex	Cat#: GTX128145; RRID: AB_2687562

bioRxiv preprint doi: https://doi.org/10.64898/2026.03.04.709614; this version posted March 6, 2026. The copyright holder for this preprint (which was not certified by peer review) is the author/funder, who has granted bioRxiv a license to display the preprint in perpetuity. It is made available under aCC-BY 4.0 International license.

REAGENT or RESOURCE	SOURCE	IDENTIFIER
Goat polyclonal anti-ATR	Santa Cruz Biotechnology	Cat#: sc-1887; RRID: AB_630893
Rabbit polyclonal anti-p53	EpiCypher	Cat#: 13-2015; RRID:AB_3697301
Mouse monoclonal anti-p53	Santa Cruz Biotechnology	Cat#: sc-126; RRID:AB_628082
Rabbit polyclonal anti-acetyl-p53 (Lys382)	Cell Signaling Technology	Cat#: 2525; RRID: AB_330083
Rabbit monoclonal anti-p21 Waf1/Cip1	Cell Signaling Technology	Cat#: 2947; RRID: AB_823586
Rabbit monoclonal anti-DHCR24	Cell Signaling Technology	Cat#: 2033; RRID: AB_2091448
Goat anti-Mouse IgG (H+L) Cross-Adsorbed Secondary Antibody, HRP	Thermo Fisher Scientific	Cat#: G-21040; RRID: AB_2536527
Goat anti-Rabbit IgG (H+L) Cross-Adsorbed Secondary Antibody, HRP	Thermo Fisher Scientific	Cat#: G-21234; RRID: AB_2536530
**Flow cytometry antibodies**		
Mouse anti-CD4 Brilliant Ultraviolet 395	Invitrogen	Cat#: 363-0047-42; RRID:AB_2925250
Mouse anti-CD8 Brilliant Ultraviolet 496	BD Biosciences	Cat#: 612942; RRID:AB_2870223
Mouse anti-CD38 Brilliant Ultraviolet 737	BD Biosciences	Cat#: 612825; RRID:AB_2870149
Mouse anti-CD39 PE-Fire 810	BioLegend	Cat#: 328245; RRID:AB_2894563
Mouse anti-HLA-DR Brilliant Violet 786	BD Biosciences	Cat#: 564041; RRID:AB_2738559
Mouse anti-PD-1 APC	BioLegend	Cat#: 329908; RRID:AB_940475
Mouse anti-LAG3 Brilliant Violet 421	BioLegend	Cat#: 369314; RRID:AB_2629797
Mouse anti-TIGIT Brilliant Ultraviolet 615	BD Biosciences	Cat#: 570444; RRID:AB_3685741
Mouse anti-CTLA4 Brilliant Violet 711	BioLegend	Cat#: 369632; RRID:AB_2892450

bioRxiv preprint doi: https://doi.org/10.64898/2026.03.04.709614; this version posted March 6, 2026. The copyright holder for this preprint (which was not certified by peer review) is the author/funder, who has granted bioRxiv a license to display the preprint in perpetuity. It is made available under aCC-BY 4.0 International license.

REAGENT or RESOURCE	SOURCE	IDENTIFIER
Mouse anti-T-Bet R718	BD Biosciences	Cat#: 568175; RRID:AB_3684101
Rabbit anti-TCF-1 Alexa Fluor 488	Cell Signaling Technology	Cat#: 6444S; RRID:AB_2797627
Human anti-TOX PE	Miltenyi Biotec	Cat#: 130-120-716; RRID:AB_2801780
Mouse anti-GzmB PerCP-Cy5.5	BioLegend	Cat#: 372212; RRID:AB_2728379
Mouse anti-IFNγ FITC	BioLegend	Cat#: 502506; RRID:AB_315231
Rat anti-IL-2 APC-Fire 750	BioLegend	Cat#: 500352; RRID:AB_2820083
Mouse anti-TNF PE	BioLegend	Cat#: 502909; RRID:AB_315261
Mouse anti-CD4 Brilliant Violet 711	BD Biosciences	Cat#: 563028; RRID:AB_2737961
Mouse anti-CD8 FITC	BioLegend	Cat#: 301050; RRID:AB_2562055
Mouse anti-CD8 Spark PLUS UV395	BioLegend	Cat#: 301079; RRID:AB_3097555
Human TruStain FcX	BioLegend	Cat#: 422302; RRID:AB_2818986
Mouse TruStain FcX	BioLegend	Cat#: 101320
Hamster anti-TCR β chain PerCP/Cyanine5.5	BioLegend	Cat#: 109228 RRID: AB_1575173
Rat anti-CD8a APC	BioLegend	Cat#: 100712 RRID: AB_312751
Rat anti-CD4 Brilliant Violet 711	BioLegend	Cat#: 100550 RRID: AB_2562099
Rat anti-CD44 PE	Invitrogen	Cat#: 12-0441-83 RRID: AB_465665
Rat anti-PD-1 PE/Cyanine7	BioLegend	Cat#: 109110 RRID: AB_572017
Mouse anti-CD3 Brilliant Violet 570	BioLegend	Cat#: 300436 RRID: AB_2562124

bioRxiv preprint doi: https://doi.org/10.64898/2026.03.04.709614; this version posted March 6, 2026. The copyright holder for this preprint (which was not certified by peer review) is the author/funder, who has granted bioRxiv a license to display the preprint in perpetuity. It is made available under aCC-BY 4.0 International license.

REAGENT or RESOURCE	SOURCE	IDENTIFIER
Mouse anti-CD56 PE/Cyanine7	BioLegend	Cat#: 362510<br>RRID: AB_2563927
Rat anti-CD11b PE/Cyanine7	BioLegend	Cat#: 101216<br>RRID: AB_312799
Mouse anti-CD19 PE/Cyanine7	BioLegend	Cat#: 363012<br>RRID: AB_2564203
Mouse anti-CD45 Brilliant Violet 421	BioLegend	Cat#: 368522<br>RRID: AB_2687375
Mouse anti-CD3 PerCP/Cyanine5.5	BioLegend	Cat#: 344808<br>RRID: AB_10640736
Rat anti-CD45 Brilliant Violet 570	BioLegend	Cat#: 103136<br>RRID: AB_2562612
Rat anti-CD11b BUV395	BD Biosciences	Cat#: 563553<br>RRID: AB_2738276
Mouse anti-NK1.1 BUV395	BD Biosciences	Cat#: 564144<br>RRID: AB_2738618
Rat anti-CD45R/B220 BUV395	BD Biosciences	Cat#: 563793<br>RRID: AB_2738427
Hamster anti-mouse TCR β chain BUV805	BD Biosciences	Cat#: 748405<br>RRID: AB_2872824
Rat anti-CD8a BUV615	BD Biosciences	Cat#: 613004<br>RRID: AB_2870272
Rat anti-CD4 PE	BioLegend	Cat#: 100408<br>RRID: AB_312693
Rat anti-CD44 FITC	Invitrogen	Cat#: 11-0441-85<br>RRID: AB_465046
Rat anti-PD-1 PE/Cyanine7	BioLegend	Cat#: 109110<br>RRID: AB_572017
Human anti-CD4 Brilliant Violet 711	BioLegend	Cat#: 300558<br>RRID: AB_2564393
Human anti-CD8 Brilliant Ultraviolet 563	BD Biosciences	Cat#: 612914<br>RRID: AB_2870200
**Flow cytometry dyes**		
Ghost Dye Violet 510 Fixable Viability Dye	Cell Signaling Technology	Cat#: 59863S
Zombie NIR	BioLegend	Cat#: 423106
Ghost Dye Red 780	Cytek Biosciences	Cat#: 13-0865-T100

bioRxiv preprint doi: https://doi.org/10.64898/2026.03.04.709614; this version posted March 6, 2026. The copyright holder for this preprint (which was not certified by peer review) is the author/funder, who has granted bioRxiv a license to display the preprint in perpetuity. It is made available under aCC-BY 4.0 International license.

REAGENT or RESOURCE	SOURCE	IDENTIFIER
FxCycle Violet	Invitrogen	Cat#: F10347
Dihydroethidium (Superoxide indicator)	Invitrogen	Cat#: D11347
MitoSOX Red (Mitochondrial superoxide indicator)	Invitrogen	Cat#: M36008
**Biological samples**		
Human PB Leukopak, Fresh	STEMCELL Technologies	Cat#: 70500.1
Human LRS Cone	STEMCELL Technologies	Cat#: 200-0093
Human buffy coat	New York Blood Center	
**Chemicals, peptides, and recombinant proteins**		
RPMI 1640 Medium, no glutamine	Thermo Fisher Scientific	Cat#: 21870-076
RPMI 1640 Medium with glutamine (Media Preparation Core, MSKCC)	Thermo Fisher Scientific	Cat#: 11875093
Penicillin-Streptomycin (10,000 U/mL)	Thermo Fisher Scientific	Cat#: 15140-122
L-Glutamine (200 mM)	Thermo Fisher Scientific	Cat#: 25030081
MEM Non-Essential Amino Acids Solution (100X)	Thermo Fisher Scientific	Cat#: 11140050
Sodium Pyruvate (100 mM)	Thermo Fisher Scientific	Cat#: 11360070
BenchMark Fetal Bovine Serum (FBS)	GeminiBio	Cat#: 100-106
Dialyzed Fetal Bovine Serum	GeminiBio	Cat#: 100-108
Lymphoprep	STEMCELL Technologies	Cat#: 07861
RBC Lysis Buffer, 10X	Santa Cruz Biotechnology	Cat#: sc-296258
EDTA (0.5 M, pH 8.0)	Thermo Fisher Scientific	Cat#: AM9260G
Monoclonal mouse anti-human CD3 (clone OKT3)	Bio X Cell	Cat#: BE0001-2; RRID: AB_1107632
Ultra-LEAF Purified anti-human CD3 Antibody (clone OKT3)	BioLegend	Cat#: 317349; RRID:AB_2749888
Monoclonal mouse anti-human CD28 (clone 9.3)	Bio X Cell	Cat#: BE0248; RRID: AB_2687729
CD3e Monoclonal Antibody (clone 145-2C11)	Thermo Fisher Scientific	Cat#: 16-0031-38; RRID:AB_2865575
CD28 Monoclonal Antibody (clone 37.51)	Thermo Fisher Scientific	Cat#: 16-0281-85; RRID:AB_468922

bioRxiv preprint doi: https://doi.org/10.64898/2026.03.04.709614; this version posted March 6, 2026. The copyright holder for this preprint (which was not certified by peer review) is the author/funder, who has granted bioRxiv a license to display the preprint in perpetuity. It is made available under aCC-BY 4.0 International license.

REAGENT or RESOURCE	SOURCE	IDENTIFIER
Human Recombinant IL-7	STEMCELL Technologies	Cat#: 78053
Human IL-7 Recombinant Protein	Thermo Fisher Scientific	Cat#: 200-07
Human Recombinant IL-15	STEMCELL Technologies	Cat#: 78031
Human IL-15 Recombinant Protein	Thermo Fisher Scientific	Cat#: 200-15
Mouse IL-2 Recombinant Protein	Thermo Fisher Scientific	Cat#: 212-12
DMEM (Dulbecco's Modified Eagle's Medium)	CORNING	Cat#: 10-013-CV
Opti-MEM Reduced Serum Medium	Thermo Fisher Scientific	Cat#: 11058021
cOmplete, Mini, EDTA-free Protease Inhibitor Cocktail	Sigma-Aldrich	Cat#: 4693159001
PhosSTOP	Sigma-Aldrich	Cat#: 4906845001
Laemmli SDS sample buffer, reducing (4X)	Thermo Fisher Scientific	Cat#: AAJ60015AC
Precision Plus Protein Dual Color Standards	BIO-RAD	Cat#: 1610374
4–20% Criterion TGX Stain-Free Protein Gels, 26 well, 15 µl	BIO-RAD	Cat#: 5678095
4–20% Mini-PROTEAN TGX Precast Protein Gels, 15-well, 15 µl	BIO-RAD	Cat#: 4561096
10x Tris/Glycine/SDS	BIO-RAD	Cat#: 1610732
Amersham Hybond P 0.45 PVDF	Cytiva	Cat#: GE10600029
Extra Thick Blot Filter Paper 8.6 x 13.5 cm	BIO-RAD	Cat#: 1703967
5x TBT Transfer Buffer, 1L	BIO-RAD	Cat#: 10026938
Dry milk powder	Research Products International	Cat#: M17200-500.0
OmniPur BSA, Fraction V, Heat Shock Isolation	Sigma-Aldrich	Cat#: 2960-500GM
Signal Enhancer HIKARI 250	Nacalai USA	Cat#: NU00102
Pierce ECL Western Blotting Substrate	Thermo Fisher Scientific	Cat#: 32106
Adenosine 5'-(tetrahydrogen triphosphate), disodium salt, hydrate	Aaron Chemicals	Cat#: AR00I7HK
Urea	Supelco	Cat#: 108487
Iodoacetamide	Sigma-Aldrich	Cat#: I1149

bioRxiv preprint doi: https://doi.org/10.64898/2026.03.04.709614; this version posted March 6, 2026. The copyright holder for this preprint (which was not certified by peer review) is the author/funder, who has granted bioRxiv a license to display the preprint in perpetuity. It is made available under aCC-BY 4.0 International license.

REAGENT or RESOURCE	SOURCE	IDENTIFIER
Dithiothreitol (DTT)	Fisher Scientific	Cat#: BP172-25
Trypsin, sequencing grade	Promega	Cat#: V5111
Trypsin/Lys-C Mix, Mass Spec Grade	Promega	Cat#: V5073
Pierce™ Streptavidin Agarose	Thermo Fisher Scientific	Cat#: 20353
Acetonitrile, LC/MS grade	Fisher Scientific	Cat#: A955
Hydroxylamine solution	Sigma-Aldrich	Cat#: 467804
EPPS	Alfa Aesar	Cat#: A13714-22
TMT10plex Isobaric Label Reagent Set	Thermo Fisher Scientific	
TMTpro 16plex Label Reagent Set	Thermo Fisher Scientific	Cat#: A44521
Intercept (TBS) Blocking Buffer	LI-COR	Cat#: 927-60001
Revert 700 Total Protein Stain	LI-COR	Cat#: 926-11021
NP-40	Millipore Sigma	Cat#: 492016
Antimycin A	VWR International Inc.	Cat#: 380-075-M010
β-Mercaptoethanol	Thermo Fisher Scientific	Cat#: 21985023
Penicillin-Streptomycin (10,000 U/mL)	Thermo Fisher Scientific	Cat#: 15140163
D-Glucose	Millipore Sigma	Cat#: G7021
L-Glutamine	Thermo Fisher Scientific	Cat#: A2916801
D-Glucose (U-$^{13}C_6$, 99%)	Cambridge Isotope Laboratories, Inc	Cat#: CLM-1396-PK
L-Glutamine ($^{13}C_5$, 99%)	Cambridge Isotope Laboratories, Inc	Cat#: CLM-1822-H-PK
Methanol, Optima LC/MS Grade	Fisher Scientific	Cat#: A456-4
EASYstrainer 40 μM	Greiner Bio-One	Cat#: 542040
EASYstrainer 100 μM	Greiner Bio-One	Cat#: 542000
Ammonium chloride	Millipore Sigma	Cat#: A9434
Potassium Bicarbonate	Fisher Scientific	Cat#: P184-500
EDTA-Na2	Millipore Sigma	Cat#: E5134
MG132	MedchemExpress	Cat#: HY-13259C
Me4BodipyFL-Ahx3Leu3VS	R&D SYSTEMS	Cat#: I-190-050
$MM(PEG)_{24}$	Thermo Fisher Scientific	Cat#: 22713

bioRxiv preprint doi: https://doi.org/10.64898/2026.03.04.709614; this version posted March 6, 2026. The copyright holder for this preprint (which was not certified by peer review) is the author/funder, who has granted bioRxiv a license to display the preprint in perpetuity. It is made available under aCC-BY 4.0 International license.

REAGENT or RESOURCE	SOURCE	IDENTIFIER
Sera-Mag SpeedBead carboxylate-modified [E7] magnetic particles	Cytiva	Cat#: 45152105050250
Sera-Mag Speedbead carboxylate-modified [E3] magnetic particles	Cytiva	Cat#: 65152105050250
Ethanol 200 Proof	Decon Labs	Ca3#: 2716
Pierce High Capacity Streptavidin Agarose	Thermo Fisher Scientific	Cat#: 20359
Lipofectamine 3000	Thermo Fisher Scientific	Cat#: L3000015
ViralBoost	ALSTEM	Cat#: VB100
Whatman Puradisc sterile PES syringe filters	Cytiva	Cat#: 6780-2504
Lentivirus Precipitation Solution	ALSTEM	Cat #: VC100
ImmunoCult Human CD3/CD28/CD2 T Cell Activator	STEMCELL Technologies	Cat#: 10970
BSJ-04-122	Millipore Sigma	Cat#: SML3061
ISRIB	MedChemExpress	Cat#: HY-12495
Thapsigargin	Millipore Sigma	Cat#: T9033
Blasticidin	Invivogen	Cat#: ant-bl-1
Q5 High-Fidelity 2x Master Mix	NEW ENGLAND Biolabs	Cat#: M0492S
MluI-HF	NEW ENGLAND Biolabs	Cat#: R3198S
SbfI-HF	NEW ENGLAND Biolabs	Cat#: R3642S
Liberase TL Research Grad	Millipore Sigma	Cat#: 05401020001
DNase I, grad II, from bovine pancreas	Millipore Sigma	Cat#: 10104159001
Hyaluronidase	Worthington Biochemical Corporation	Cat#: LS002592
HEPES, Liquid 1M Solution	Corning	Cat#: 25060CI
gentleMACS C tubes	Miltenyi Biotec	Cat#: 130-093-237
poly-*L*-lysine solution	Millipore Sigma	Cat#: P4707
XF RPMI medium	Agilent	Cat#: 103576-100
XF glucose solution	Agilent	Cat#: 103577-100
XF pyruvate solution	Agilent	Cat#: 103578-100
**Critical commercial assays**		
DC Protein Assay Kit	BIO-RAD	Cat#: 5000111

bioRxiv preprint doi: https://doi.org/10.64898/2026.03.04.709614; this version posted March 6, 2026. The copyright holder for this preprint (which was not certified by peer review) is the author/funder, who has granted bioRxiv a license to display the preprint in perpetuity. It is made available under aCC-BY 4.0 International license.

REAGENT or RESOURCE	SOURCE	IDENTIFIER
EasySep Human T cell isolation kit	STEMCELL Technologies	Cat#: 17951
Dynabeads Untouched Human T Cells Kit	Thermo Fisher Scientific	Cat#: 11344D
Dynabeads Untouched Mouse T Cells Kit	Thermo Fisher Scientific	Cat#: 11413D
Dynabeads™ FlowComp™ Mouse Pan T (CD90.2) Kit	Thermo Fisher Scientific	Cat#: 11465D
Dynabeads Untouched Mouse CD8 Cells Kit	Thermo Fisher Scientific	Cat#: 11417D
Micro BCA Protein Assay Kit	Thermo Fisher Scientific	Cat#: 23235
Pierce C18 pipette tips	Thermo Fisher Scientific	Cat#: 87784
Sep-Pak Vac C18 cartridges	Waters	Cat#: WAT054955
eBioscience Intracellular Fixation & Permeabilization Buffer Set	Invitrogen	Cat#: 88-8824-00
BD Biosciences Cytofix/Cytoperm kit	BD Biosciences	Cat#: 554714
Click-iT Plus EdU Alexa Fluor 594 Flow Cytometry Assay Kit	Thermo Fisher Scientific	Cat#: C10646
Brilliant Stain Buffer	BD Biosciences	Cat#: 566349
UltraComp eBeads Plus Compensation Beads	Invitrogen	Cat#: 01-3333-42
ONE-Glo EX Luciferase Assay System	Promega	Cat#: E8110
Cell Culture Microplate, 96 Well, PS, F-bottom, Chimney Well, white, Sterile	Greiner	Cat#: 655073
XFe96/XF Pro cell culture microplates	Agilent	Cat#: 130-093-237
TruSeq Stranded mRNA LT Kit	Illumina, Inc	Cat#: RS-122-2102
NovaSeq 6000 S4 Reagent Kit v1.5	Illumina, Inc	Cat#: 20028313
10x Genomics Next GEM Single Cell 3’ Gene Expression Kit, v3.1	10x Genomics	Cat#: 1000268
MAS-Seq Kit	PacBio	Cat#: 102-659-600
Ultrafree Centrifugal Filter, 0.5 mL Sample Volume	Millipore	Cat#: UFC30GV25
Pierce High pH Reversed-Phase Peptide Fractionation Kit	Thermo Fisher Scientific	Cat#: 84868
Q5 Site-Directed Mutagenesis Kit	NEW ENGLAND Biolabs	Cat#: E0554S
Seahorse XF Cell Mito Stress Test	Agilent	Cat#: 103015-100

bioRxiv preprint doi: https://doi.org/10.64898/2026.03.04.709614; this version posted March 6, 2026. The copyright holder for this preprint (which was not certified by peer review) is the author/funder, who has granted bioRxiv a license to display the preprint in perpetuity. It is made available under aCC-BY 4.0 International license.

REAGENT or RESOURCE	SOURCE	IDENTIFIER
Human Tumor Dissociation Kit	Miltenyi Biotec	Cat#: 130-095-929
**Deposited data**		
All RNA-sequencing data have been uploaded to GEO	This paper	GSE: TBD
All raw proteomic data has been uploaded to PRIDE	This paper	PRIDE: TBD
All single-cell RNA-sequencing data have been uploaded to GEO	This paper	GSE: TBD
**Experimental Models: Cell lines**		
Lenti-X 293T Cell Line	TaKaRa	Cat#: 632180
A375	ATCC	Cat#: CRL-1619
SK-MEL-37	Antibody and Bioresource Core Facility at MSKCC and RU	Cat#: CVCL_3878
A375-NLS-mCherry	This paper	N/A
SK-MEL-37-NLS-mCherry	This paper	N/A
**Experimental models: Organisms/strains**		
Mouse: C57BL/6J	Jackson Laboratory	Cat#: 000664; RRID: IMSR_JAX:000664
Lymphocytic choriomeningitis virus (LCMV) Armstrong	Salk Institute for Biological Studies	
Lymphocytic choriomeningitis virus (LCMV) Clone 13	Salk Institute for Biological Studies	
2838c3 KPC	This paper	
**Recombinant DNA**		
Lenti-LucOS	Addgene	Cat#: 22777
pMD2.G	Addgene	Cat#: 12259
psPAX2	Addgene	Cat#: 12260
pZR071	Addgene	Cat#: 180264
pZR071_1G4_WT	This paper	Generated for this paper based on Robbins et al.[1]

bioRxiv preprint doi: https://doi.org/10.64898/2026.03.04.709614; this version posted March 6, 2026. The copyright holder for this preprint (which was not certified by peer review) is the author/funder, who has granted bioRxiv a license to display the preprint in perpetuity. It is made available under aCC-BY 4.0 International license.

REAGENT or RESOURCE	SOURCE	IDENTIFIER
p pZR071_1G4-95LY	This paper	Generated for this paper based on Robbins et al.[1]
pMSGV1_mCherry-NLS_P2A_blastR	This paper	Generated for this paper based on Yi et al.[2]
**Software and algorithms**		
RAW Converter	version 1.1.0.22; 2004 release	fields.scripps.edu/rawconv
Integrated Proteomics Pipeline (IP2) and ProLuCID	Integrated Proteomics Applications, Inc	goldfish.scripps.edu
Prism (v9.3.1)	GraphPad Software Inc	graphpad.com/scientific-software/prism
SpectroFlo	Cytek Biosciences	cytekbio.com/pages/spectro-flo
FlowJo (v10.8.2)	Treestar Inc.	flowjo.com
ImageJ	NIH	imagej.nih.gov/ij
PyMOL (v2.5.5)	Schrödinger	
Trim_galore (v0.6.4)	Martin et al.[3]	github.com/FelixKrueger/TrimGalore
STAR (v2.7.5)	Dobin et al.[4]	github.com/alexdobin/STAR
samtools (v1.9)	Danecek et al.[5]	htslib.org
bamCoverage (part of the Deeptools package; v3.3.1)	Ramirez et al.[6]	deeptools.readthedocs.io/en/develop/index.html
featureCounts (part of the subread package; v1.5.0)	Liao et al.[7]	subread.sourceforge.net
edgeR (v3.32.1)	Robinson et al.[8]	bioconductor.org/packages/release/bioc/html/edgeR.html
DESeq2 (v1.30.1)	Love et al.[9]	bioconductor.org/packages/release/bioc/html/DESeq2.html

bioRxiv preprint doi: https://doi.org/10.64898/2026.03.04.709614; this version posted March 6, 2026. The copyright holder for this preprint (which was not certified by peer review) is the author/funder, who has granted bioRxiv a license to display the preprint in perpetuity. It is made available under aCC-BY 4.0 International license.

REAGENT or RESOURCE	SOURCE	IDENTIFIER
limma voom (v3.46.0)	Law et al.[10]	bioconductor.org/packages/release/bioc/html/limma.html
rMATS (v4.1.1)	Shen et al.[11]	rnaseq-mats.sourceforge.net/rmats4.1.1/index.html
ggplot2 (v3.3.5)	Wikham H.[12]	ggplot2.tidyverse.org
ggrepel (v0.9.1)	Slowikowski et al.	github.com/slowkow/ggrepel
maser (v1.8.0)	Veiga, D.F.T.[13]	bioconductor.org/packages/release/bioc/html/maser.html
Python (v3.12.3)	Python Software Foundation	python.org/doc/versions
Seaborn (v.0.11.2)	Waskom et al., 2021[14]	seaborn.pydata.org
Plotly (v5.11.0)	Plotly Technologies Inc. Collaborative data science. Montréal, QC, 2015. plot.ly	plotly.com/python
Matplotlib (v3.8.4)	Hunter et al., 2007[15]	matplotlib.org
Scikit-learn (v1.4.2)	Pedregosa et al., 2011[16]	scikit-learn.org/stable
Pandas (v2.2.2)	McKinney, 2010[17]	pandas.pydata.org
Numpy (v1.26.4)	Van der Walt et al., 2011[18]	numpy.org
Skera (v1.3.0)	Pacific Biosciences	skera.how/
lima v2.12.0	Pacific Biosciences	lima.how/
Iso-Seq (v4.0.0)	Pacific Biosciences	isoseq.how/
pbmm2 (v1.16.99)	Pacific Biosciences	github.com/PacificBiosciences/pbmm2
IsoQuant (v3.3.1)	Prjibelski et al., 2023[19]	github.com/ablab/IsoQuant
Seurat (v5.1.0)	Hao et al. 2023[20]	satijalab.org/seurat/
Doublet Detection (v4.2)	Gayoso et al. 2020	github.com/JonathanShor/DoubletDetection

bioRxiv preprint doi: https://doi.org/10.64898/2026.03.04.709614; this version posted March 6, 2026. The copyright holder for this preprint (which was not certified by peer review) is the author/funder, who has granted bioRxiv a license to display the preprint in perpetuity. It is made available under aCC-BY 4.0 International license.

REAGENT or RESOURCE	SOURCE	IDENTIFIER
imputeLCMD	Lazar et al. 2015	cran.r-project.org/package=imputeLCMD
GSEAPY	Fang et al. 2022	github.com/zqfang/GSEApy
GOATOOLS	Klopfenstein et al. 2018[21]	github.com/tanghaibao/goatools
MassHunter Profinder software v.10.0	Agilent Technologies, Inc.	www.agilent.com/en/product/software-informatics/mass-spectrometry-software/data-analysis/mass-profiler-professional-software
MS-DIAL software[22]	Tsugawa et al. 2015[22]	systemsomicslab.github.io/compms/msdial/main.html
AlphaFold3 (AF3, version 3.0.0)	Abramson et al. 2024[23]	github.com/google-deepmind/alphafold3
Incucyte 2023A Rev2 GUI	Sartorius	https://downloads.essenbioscience.com/downloads-categories/incucyte-gui
Seahorse Analytics XF software	Agilent	https://www.agilent.com/en/product/cell-analysis/real-time-cell-metabolic-analysis/xf-software/agilent-seahorse-analytics-787485

bioRxiv preprint doi: https://doi.org/10.64898/2026.03.04.709614; this version posted March 6, 2026. The copyright holder for this preprint (which was not certified by peer review) is the author/funder, who has granted bioRxiv a license to display the preprint in perpetuity. It is made available under aCC-BY 4.0 International license.

Key Resource Table References:

1. Robbins, P.F., Li, Y.F., El-Gamil, M., Zhao, Y., Wargo, J.A., Zheng, Z., Xu, H., Morgan, R.A., Feldman, S.A., Johnson, L.A., et al. (2008). Single and dual amino acid substitutions in TCR CDRs can enhance antigen-specific T cell functions. J Immunol *180*, 6116-6131. 10.4049/jimmunol.180.9.6116.
2. Yi, F., Cohen, T., Zimmerman, N., Dundar, F., Zumbo, P., Eltilib, R., Brophy, E.J., Arkin, H., Feucht, J., Gormally, M.V., et al. (2024). CAR-engineered lymphocyte persistence is governed by a FAS ligand/FAS auto-regulatory circuit. bioRxiv. 10.1101/2024.02.26.582108.
3. Martin, M. (2011). Cutadapt removes adapter sequences from high-throughput sequencing reads. EMBnet J. *17*, 10-12. 10.14806/ej.17.1.200.
4. Dobin, A., Davis, C.A., Schlesinger, F., Drenkow, J., Zaleski, C., Jha, S., Batut, P., Chaisson, M., and Gingeras, T.R. (2013). STAR: ultrafast universal RNA-seq aligner. Bioinformatics *29*, 15-21. 10.1093/bioinformatics/bts635.
5. Danecek, P., Bonfield, J.K., Liddle, J., Marshall, J., Ohan, V., Pollard, M.O., Whitwham, A., Keane, T., McCarthy, S.A., Davies, R.M., and Li, H. (2021). Twelve years of SAMtools and BCFtools. Gigascience *10*. 10.1093/gigascience/giab008.
6. Ramirez, F., Ryan, D.P., Gruning, B., Bhardwaj, V., Kilpert, F., Richter, A.S., Heyne, S., Dundar, F., and Manke, T. (2016). deepTools2: a next generation web server for deep-sequencing data analysis. Nucleic Acids Res *44*, W160-165. 10.1093/nar/gkw257.
7. Liao, Y., Smyth, G.K., and Shi, W. (2014). featureCounts: an efficient general purpose program for assigning sequence reads to genomic features. Bioinformatics *30*, 923-930. 10.1093/bioinformatics/btt656.
8. Robinson, M.D., and Oshlack, A. (2010). A scaling normalization method for differential expression analysis of RNA-seq data. Genome Biol *11*, R25. 10.1186/gb-2010-11-3-r25.
9. Love, M.I., Huber, W., and Anders, S. (2014). Moderated estimation of fold change and dispersion for RNA-seq data with DESeq2. Genome Biol *15*, 550. 10.1186/s13059-014-0550-8.
10. Law, C.W., Chen, Y., Shi, W., and Smyth, G.K. (2014). voom: Precision weights unlock linear model analysis tools for RNA-seq read counts. Genome Biol *15*, R29. 10.1186/gb-2014-15-2-r29.
11. Shen, S., Park, J.W., Lu, Z.X., Lin, L., Henry, M.D., Wu, Y.N., Zhou, Q., and Xing, Y. (2014). rMATS: robust and flexible detection of differential alternative splicing from replicate RNA-Seq data. Proc Natl Acad Sci U S A *111*, E5593-5601. 10.1073/pnas.1419161111.
12. Wickham, H. (2016). ggplot2: Elegant Graphics for Data Analysis (Springer-Verlag New York).
13. Veiga, D.F.T. (2023). maser: Mapping Alternative Splicing Events to proteins.
14. Waskom, M.L. (2021). seaborn: statistical data visualization. . Journal of Open Source Software *6*, 3021. 10.21105/joss.03021.
15. Hunter, J.D. (2007). Matplotlib: A 2D graphics environment. . Computing in Science & Engineering *9*, 90-95. 10.1109/MCSE.2007.55.
16. Pedregosa, F.V., G.; Gramfort, A.; Michel, V.; Thirion, B.; Grisel, O.; Blondel, M.; Prettenhofer, P.; Weiss, R.; Dubourg, V.; Vanderplas, J.; Passos, A.; Cournapeau, D.; Brucher, M.; Perrot, M.; Duchesnay, E. (2011). Scikit-learn: Machine Learning in Python. Journal of Machine Learning Research. *12*, 2825-2830.
17. McKinney, W. (2010). pp. 51-56.

bioRxiv preprint doi: https://doi.org/10.64898/2026.03.04.709614; this version posted March 6, 2026. The copyright holder for this preprint (which was not certified by peer review) is the author/funder, who has granted bioRxiv a license to display the preprint in perpetuity. It is made available under aCC-BY 4.0 International license.

18. Van Der Walt, S.C., S. C.; Varoquaux, G. (2011). The NumPy Array: A Structure for Efficient Numerical Computation. . Computing in Science & Engineering *13*, 22-30. 10.1109/MCSE.2011.37.
19. Prjibelski, A.D., Mikheenko, A., Joglekar, A., Smetanin, A., Jarroux, J., Lapidus, A.L., and Tilgner, H.U. (2023). Accurate isoform discovery with IsoQuant using long reads. Nat Biotechnol *41*, 915-918. 10.1038/s41587-022-01565-y.
20. Hao, Y., Stuart, T., Kowalski, M.H., Choudhary, S., Hoffman, P., Hartman, A., Srivastava, A., Molla, G., Madad, S., Fernandez-Granda, C., and Satija, R. (2024). Dictionary learning for integrative, multimodal and scalable single-cell analysis. Nat Biotechnol *42*, 293-304. 10.1038/s41587-023-01767-y.
21. Klopfenstein, D.V., Zhang, L., Pedersen, B.S., Ramirez, F., Warwick Vesztrocy, A., Naldi, A., Mungall, C.J., Yunes, J.M., Botvinnik, O., Weigel, M., et al. (2018). GOATOOLS: A Python library for Gene Ontology analyses. Sci Rep *8*, 10872. 10.1038/s41598-018-28948-z.
22. Tsugawa, H., Cajka, T., Kind, T., Ma, Y., Higgins, B., Ikeda, K., Kanazawa, M., VanderGheynst, J., Fiehn, O., and Arita, M. (2015). MS-DIAL: data-independent MS/MS deconvolution for comprehensive metabolome analysis. Nat Methods *12*, 523-526. 10.1038/nmeth.3393.
23. Abramson, J., Adler, J., Dunger, J., Evans, R., Green, T., Pritzel, A., Ronneberger, O., Willmore, L., Ballard, A.J., Bambrick, J., et al. (2024). Accurate structure prediction of biomolecular interactions with AlphaFold 3. Nature *630*, 493-500. 10.1038/s41586-024-07487-w.
